# Iridium-Catalyzed 1,3-Hydrogen Shift/Chlorination of Allylic Alcohols**

**DOI:** 10.1002/anie.201301013

**Published:** 2013-04-24

**Authors:** Nanna Ahlsten, Antonio Bermejo Gómez, Belén Martín-Matute

**Affiliations:** Department of Organic Chemistry, Stockholm University10691 Stockholm (Sweden)

**Keywords:** allylic alcohols, chlorination, hydrogen transfer, iridium, isomerization

Chlorinated compounds are among the most common and versatile building blocks in organic synthesis. Among these, α-chlorocarbonyl derivatives are of synthetic value owing to the variety of functional groups that can be introduced both at the chlorinated α-carbon atom and at the carbonyl functionality.[1] For instance, they readily undergo substitution/addition reactions[1, 2] and cross-coupling reactions[3] and are useful precursors to heterocycles.[4]

While a number of methods have been reported for the electrophilic halogenation of aldehydes or ketones containing only one enolizable position, the same reaction for unsymmetrical aliphatic ketones is challenging.[1] Here, most methods rely on steric or electronic differentiation for regioselective functionalization of carbonyl compounds (Scheme 1a).[1, 5] However, many ketones lack the bias to enolize with complete regioselectivity, and the enolization cannot always be directed to the desired position. Importantly, the formation of mixtures of halocarbonyl compounds limits both the yield and the overall synthetic utility owing to the challenge of separating constitutional isomers.

**Scheme 1:**
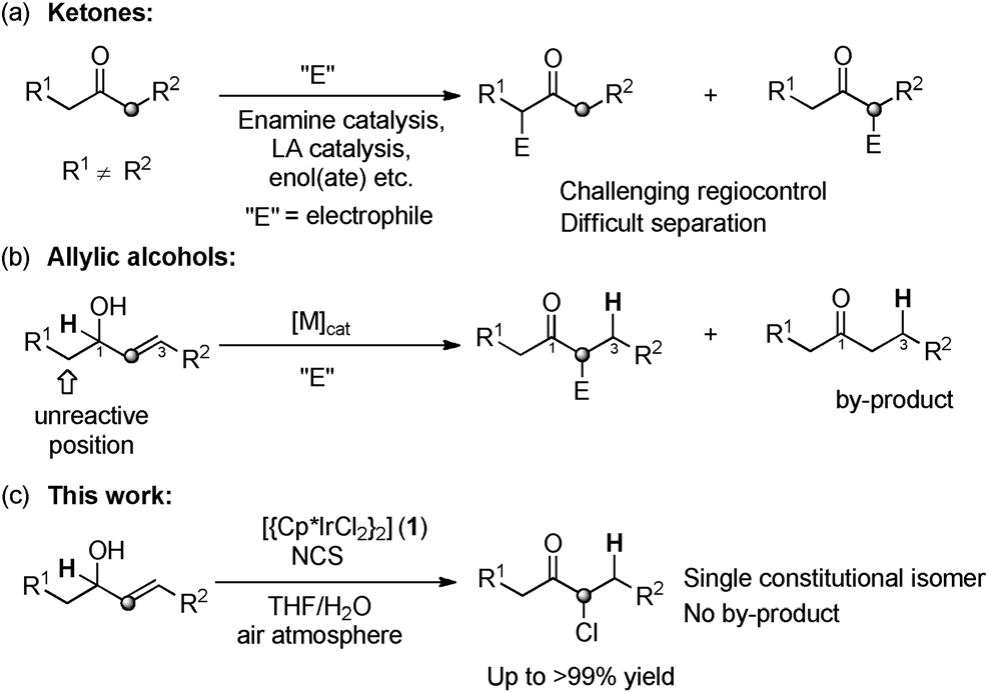
Synthesis of α-functionalized ketones through a) enolization/enamine formation; b) transition-metal-catalyzed isomerization of allylic alcohols. c) This work. LA=lewis acid; Cp*=pentamethyl cyclopentadienyl; NCS=*N*-chlorosuccinimide.

We envisaged that α-chloroketones could be synthesized with complete regiocontrol from allylic alcohols through a 1,3-hydrogen shift/chlorination catalyzed by transition metals. A considerable advantage of using allylic alcohols as enol equivalents[6, 7] is that the new bond (to the electrophile) is formed exclusively at the alkenylic carbon atom of the allylic alcohol [RCH(OH)–**C**H=CHR; Scheme 1b]. This type of transformation has almost exclusively been investigated using carbon electrophiles (e.g. aldehydes or imines).[8, 9a,9b] A drawback with all these procedures has always been the undesired formation of unfunctionalized ketone by-products (Scheme 1b). Recently, we reported the first example of 1,3-hydrogen shift/halogenation for the preparation of α-fluoroketones.[9c,9d] While this represented a success in terms of merging a transition-metal-catalyzed isomerization with an electrophilic halogenation, the formation of nonfluorinated ketones (5–20 %) could not be avoided and led to challenging separations. Herein, we describe the first chlorination of allylic alcohols, which affords single constitutional isomers of α-chloroketones in up to>99 % yield, and for the first time the formation of ketone by-products is completely suppressed (Scheme 1c).

We first investigated the isomerization/chlorination of phenylpent-1-en-3-ol (**2 a**) catalyzed by [{Cp*IrCl_2_}_2_] in the presence of *N*-chlorosuccinimide (NCS). In THF and at room temperature, only traces of the desired monochlorinated carbonyl compound **3 a** were formed together with a complicated mixture of by-products (Table 1, entry 1). However, introducing water as a cosolvent had a strong influence on the reaction outcome, and the yield of **3 a** gradually increased (entries 2, 3). Notably, in THF/H_2_O=1:2, a quantitative yield of **3 a** was obtained in 6 h with only 0.25 mol % of [{Cp*IrCl_2_}_2_] (entry 4). Under these conditions, **3 a** was formed with complete regiocontrol and nonchlorinated ketone **4 a** or enone **5 a** were not detected. Furthermore, the reactions do not require inert conditions. In the absence of THF, a low yield of **3 a** was obtained (entry 5). Chloramine-T or 1,3-dichloro-5,5-dimethylhydantoin afforded poor yields of **3 a** (entries 6–7).

**Table 1:** Isomerization/chlorination of **2 a.[**a] 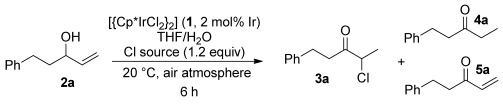

Entry	THF/H_2_O (*v*/*v*)	Cl source	Conv. of2 a[%][b]	3 a/4 a/5 a[%][b]
1	100:0	NCS	58	1:–:–
2[c]	20:1	NCS	90	3:2:1
3	5:1	NCS	85	50:4:–
**4**[d,e]	**1:2**	**NCS**	**>99**	**>99:–:–**
5	0:100	NCS	34	30:–:4
6	1:2	1,3-dichloro-5,5-dimethylhydantoin	>99	8:2:2
7	1:2	chloramine-T	30	2:2:–

[a] **2 a** (0.4 mmol, 0.2 M).

[b] Determined by ^1^H NMR spectroscopy with respect to an internal standard (1,2,4,5-tetrachloro-3-nitrobenzene).

[c] 16 h.

[d] 0.25 mol % of [{Cp^*^IrCl_2_}_2_] (0.5 mol % Ir).

[e] THF/H_2_O (*v*/*v* 1:1) afforded also >99 % yield of **3 a**.

To evaluate the reaction scope, a variety of aliphatic and α-aryl allylic alcohols (**2 a**–**2 o**), including cyclic and functionalized substrates, were subjected to the optimized reaction conditions (Scheme 2). The corresponding α-chloroketones (**3 a**–**3 o**) were obtained as single constitutional isomers in excellent yields. Unfunctionalized ketones (**4**) or side products derived from overchlorination or from chlorination of benzylic positions were not detected for any of the substrates. The enantiopure allylic alcohol **2 k** underwent the chlorination reaction without epimerization of the stereogenic center. Allylic alcohols with trisubstituted or 1,1-disubstituted olefins did not give satisfactory yields (see the Supporting Information). The methodology is suitable for multigram-scale reactions, and the chlorination of **2 e** and **2 o** gave the same yields on a 10 g scale (52–78 mmol) as on a smaller scale (1 mmol).

**Scheme 2:**
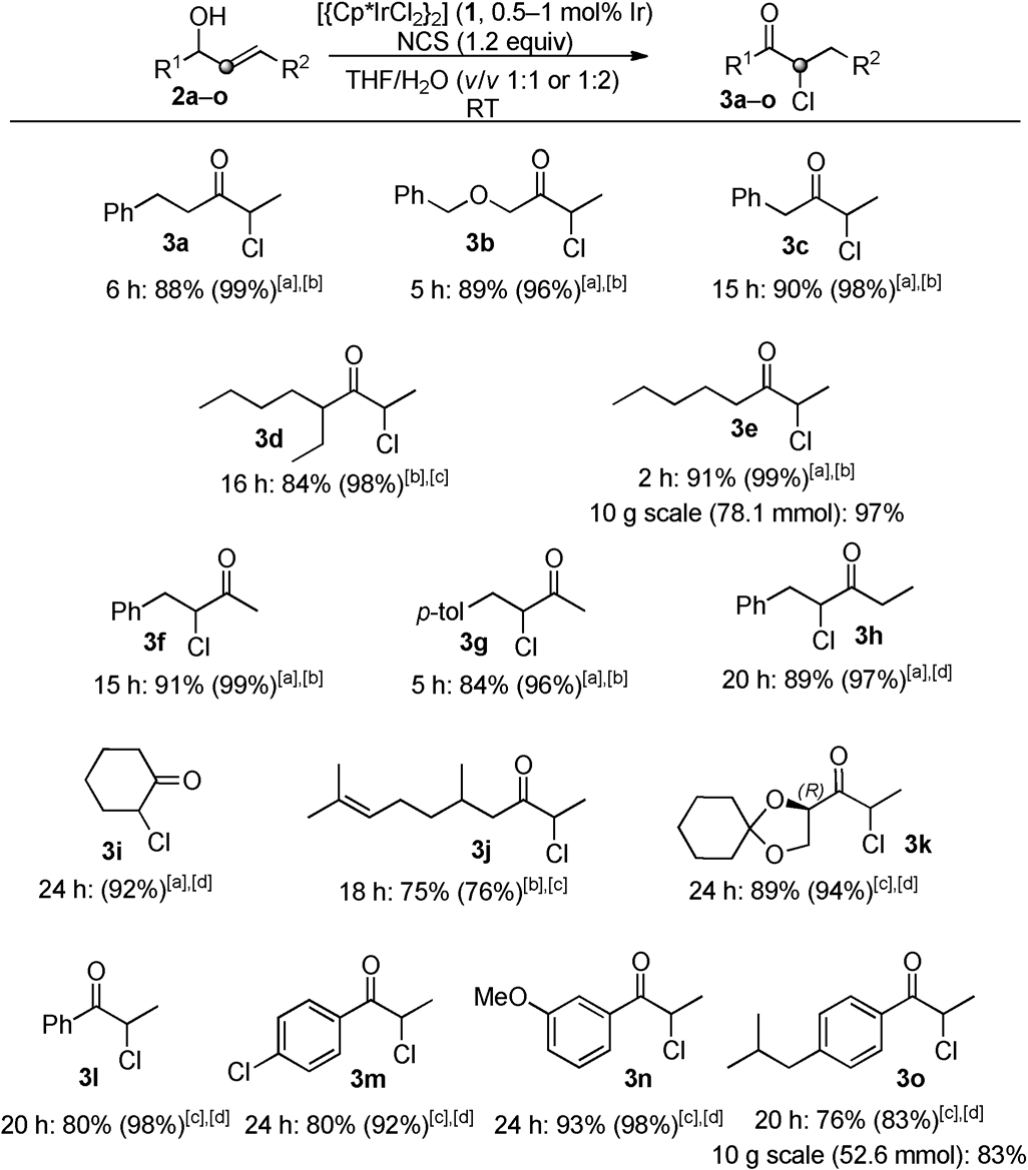
Isomerization/chlorination of *sec*-allylic alcohols. Reactions were performed on a 1 mmol scale. *trans*-2 f, 2 g, and 2 h were used. Yields of isolated products are shown (yields given in parentheses were determined by ^1^H NMR spectroscopy with respect to 1,2,4,5-tetrachloro-3-nitrobenzene as internal standard). [a] THF/H_2_O (*v*/*v* 1:2). [b] 0.25 mol % of [{Cp*IrCl_2_}_2_]. [c] THF/H_2_O (*v*/*v* 1:1). [d] 0.5 mol % of [{Cp*IrCl_2_}_2_].

The corresponding transformation of primary allylic alcohols into α-functionalized aldehydes is extremely rare.[8g, 10] Often, transition metal catalysts fail to promote the tandem processes from these substrates, and side reactions such as decarbonylation[11] and self-condensation[8g, 12] can occur with the aldehyde products. However, with the mild reaction conditions reported herein, various α-chloroaldehydes (**7 a**–**f**) were prepared in good yields from primary allylic alcohols **6 a**–**f** (Scheme 3). In some cases, yields were slightly lower owing to the volatility of the product (**7 a**), formation of enones (**7 b, 7 d**–**e**), or incomplete conversion (**7 f**).

**Scheme 3:**
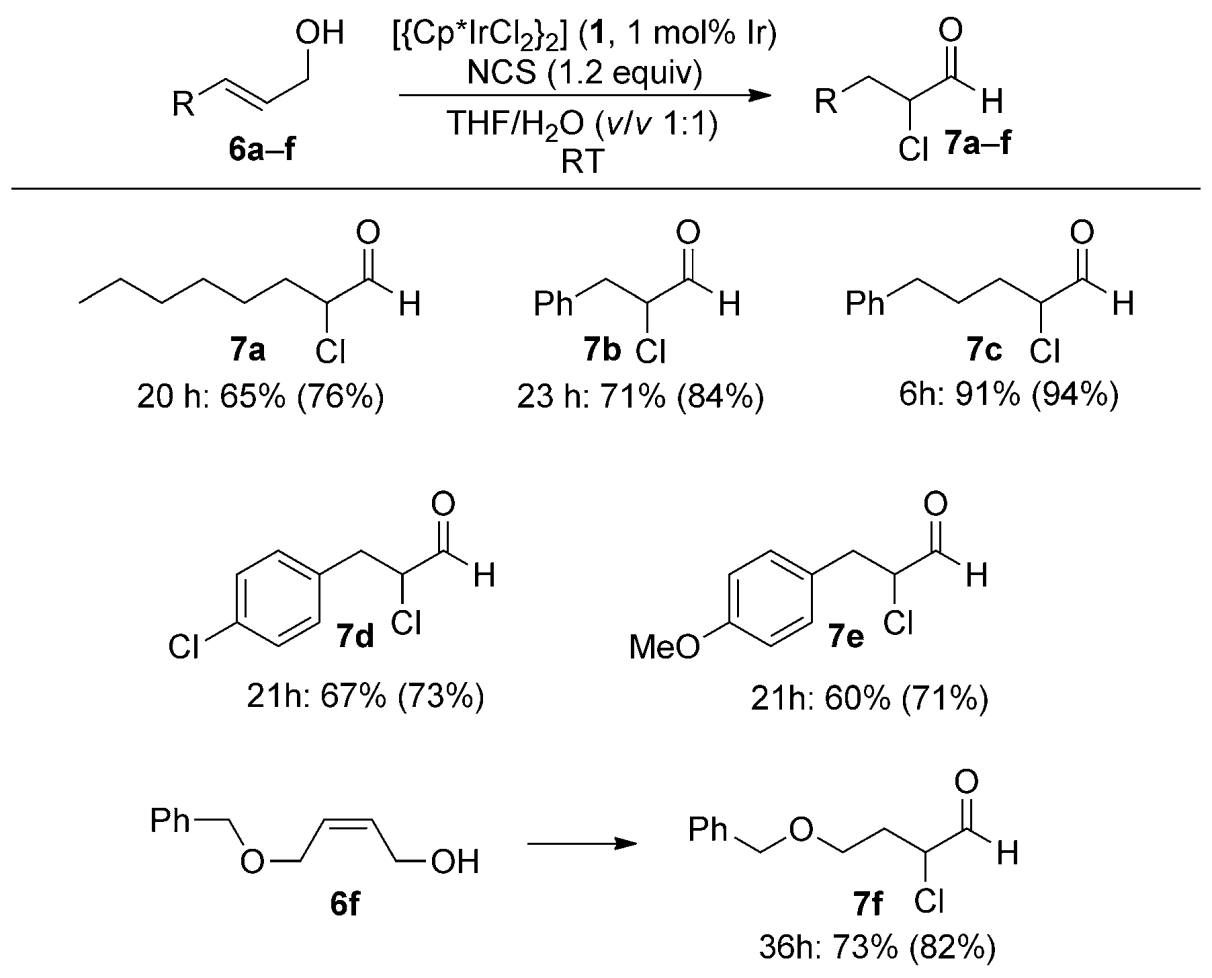
Isomerization/chlorination of primary allylic alcohols. Reactions were performed on a 1 mmol scale. *trans*-6 a–e were used. Yields of isolated products are shown (yields given in parentheses were determined by ^1^H NMR spectroscopy with respect to 1,2,4,5-tetrachloro-3-nitrobenzene as internal standard).

The varied results obtained in different mixtures of THF and H_2_O (Table 1) indicate the need for a highly polar solvent and may be related to catalyst activation through dissociation of a chloride ligand from **1**. A comparison of the catalytic activity of **1** with that of cationic [Cp*Ir(H_2_O)_3_]SO_4_ (**8**)[13] in the chlorination of **2 a** (THF/H_2_O=1:2) resulted in identical reaction profiles. This result suggests that **1** and **8** are equally capable of forming an active catalyst at this THF/H_2_O ratio (Figure 1 and Figure S1 in the Supporting Information). However, by progressively decreasing the amount of water in the solvent, significant differences between **1** and **8** were observed. Thus, while severely diminished yields were obtained with **1** (Figure 1, middle row), the yields with aqua complex **8** were unaffected even at a THF/H_2_O ratio of 20:1 (back row). Furthermore, addition of NaCl to **8** gave results comparable to those obtained with **1** (front vs. middle row). The requirement for an aqueous solvent (Table 1) is therefore likely associated with chloride dissociation from **1**. Still, water cannot be fully excluded from the solvent even with the chloride-free complex **8**; in pure THF **8** afforded **3 a** in only 40 % yield together with 22 % of the nonchlorinated ketone **4 a** (back row).[14]

**Figure 1:**
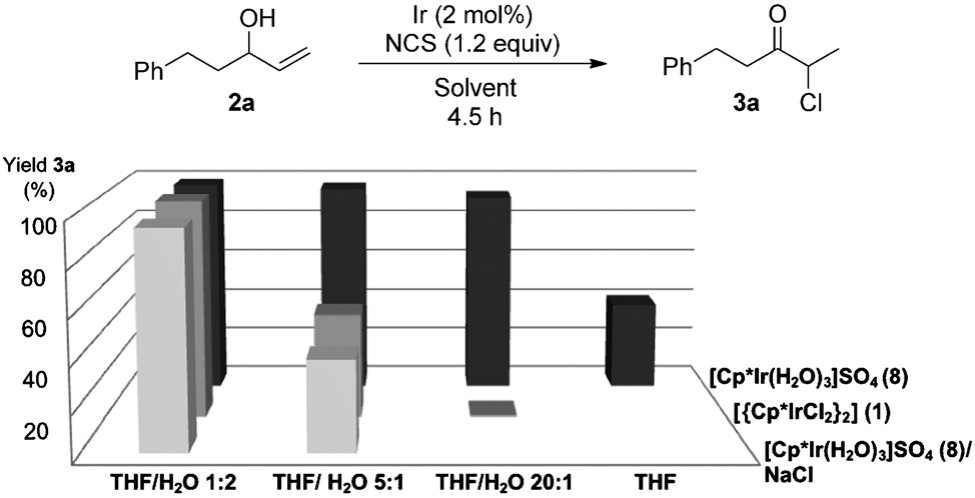
Reaction of 2 a with NCS in THF/H_2_O mixtures after 4.5 h with 8 (back), 1 (middle), and 8/NaCl (5 mol %) (front). The yield with 1 in THF/H_2_O=5:1 is the average of four reactions.

Previous studies support that the formation of α-functionalized carbonyl compounds from allylic alcohols occurs through: 1) a transition-metal-catalyzed 1,3-hydrogen shift forming an enolate or an enol intermediate, and 2) subsequent nucleophilic attack on the electrophile (Scheme 4).[6–9] In a cross-over experiment using deuterium-labeled [D_1_]-**2 f**, we have confirmed that the isomerization follows an intramolecular 1,3-hydrogen shift (Scheme 5 and the Supporting Information).[7b, 8] Although this result is consistent with the intermediacy of enol(ate)s, the details of the mechanism, and in particular of the C–Cl bond formation step, remain to be elucidated.

**Scheme 4:**
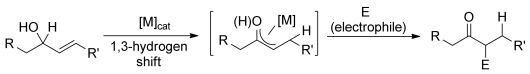
Transformation of allylic alcohols via enol intermediates.

**Scheme 5:**
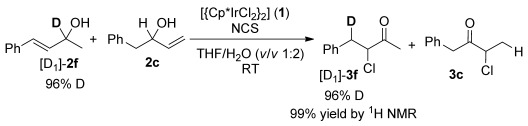
Cross-over and deuterium labeling experiment (see the Supporting Information).

To demonstrate the potential of the present methodology, the procedure was used as a key step in the synthesis of 2-aminothiazoles. These are privileged structures that have found pharmaceutical applications such as in antibiotics[15] and anti-inflammatory drugs.[16] Despite their straightforward synthesis by condensation of thiourea with the corresponding α-chlorocarbonyl compounds, only a few 4,5-disubstituted 2-aminothiazoles have been reported.[17] We reasoned that the substitution pattern of these compounds could easily be varied by using a synthetic route from allylic alcohols that gives access to the appropriate chloroketone precursor. We show here a short synthesis of 2-aminothiazoles (**9**–**12**) from allylic alcohols (**2 f**, **2 c**, **2 o**, and **2 e**, respectively, Scheme 6). The straightforward and high-yielding reactions illustrate the usefulness of this procedure in the preparation of target compounds that rely on α-chlorocarbonyl compounds as intermediates.

**Scheme 6:**
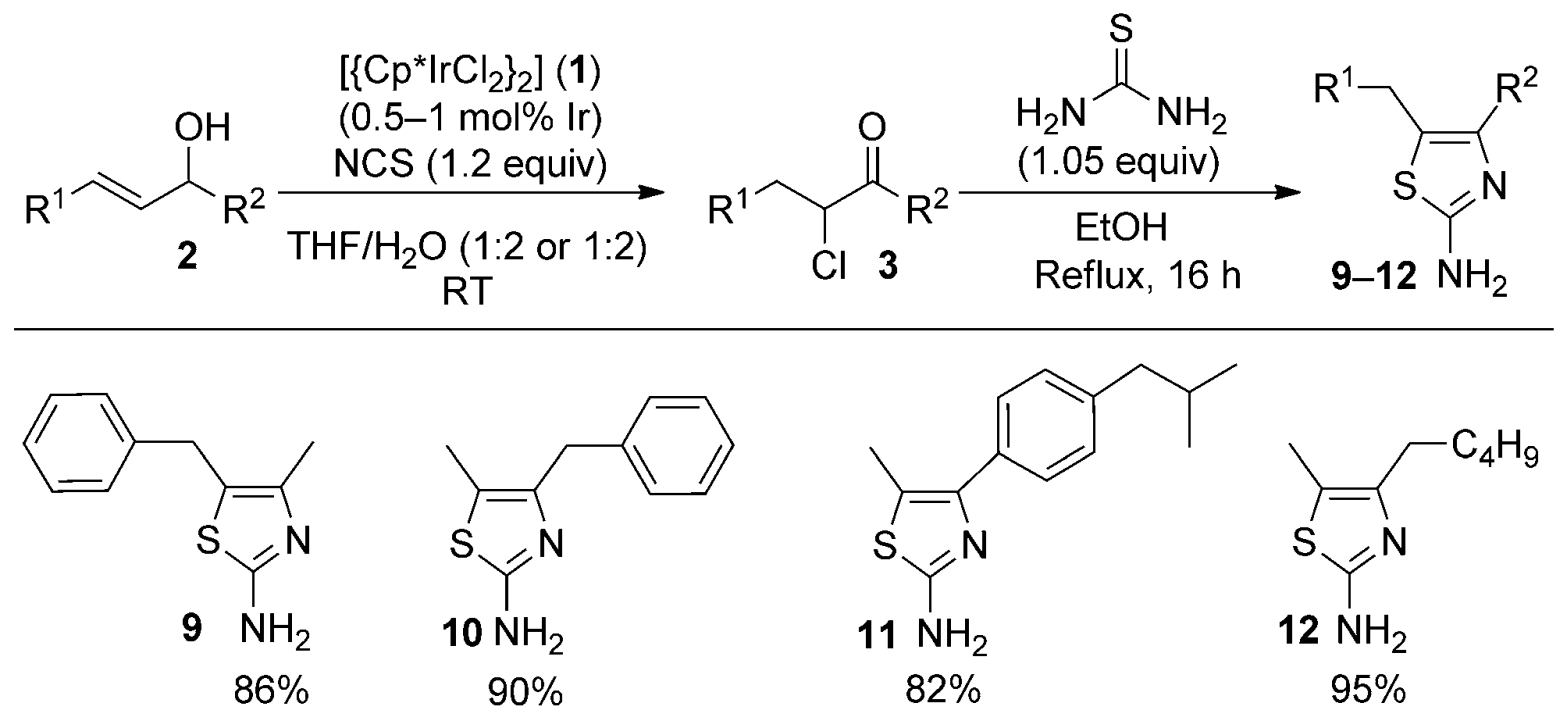
Synthesis of 2-aminothiazoles from allylic alcohols. For details see the Supporting Information. Yields of isolated products over 2 steps are shown.

In conclusion, we report the first synthesis of α-chlorinated carbonyl compounds from secondary and primary allylic alcohols. A wide range of substrates were chlorinated in high yields and as single constitutional isomers. For the first time, the formation of nonfunctionalized ketone by-products has been suppressed. The reactions are air-tolerant, run in water/organic solvent mixtures at room temperature, and require low loadings of iridium. The methodology is operationally very simple and can be scaled up. On-going mechanistic investigations should also contribute to the future development of Ir-catalyzed reactions for the formation of carbon–heteroatom bonds, and will be reported in due course.

## Experimental Section

[{Cp*IrCl_2_}_2_] (**1**, 0.25–0.5 mol %) was dissolved in THF/H_2_O (4.8 mL; 1:1 or 1:2). The allylic alcohol (1 mmol, 1 equiv) and NCS (1.2 equiv) were added, and the reaction was stirred at room temperature until full conversion. The mixture was extracted with Et_2_O (3×1 mL) and the organic layers dried over MgSO_4_ and evaporated. Purification by silica column chromatography (pentane/Et_2_O) afforded the α-chlorinated ketone/aldehyde.

## References

[1] Kimpe NDe, Verhé R (1990). The Chemistry of α-Haloketones, α-Haloaldehydes and α-Haloimines.

[2a] Yasuda M, Tsuchida M, Baba A (1998). Chem. Commun.

[2b] Malkov AV, Stončius S, Kočovský P (2007). Angew. Chem.

[2c] Shibatomi K, Yamamoto H (2008). Angew. Chem.

[2d] Lai P-S, Dubland JA, Sarwar MG, Chudzinski MG, Taylor MS (2011). Tetrahedron.

[3a] Malosh CF, Ready JM (2004). J. Am. Chem. Soc.

[3b] Liu C, He C, Shi W, Chen M, Lei A (2007). Org. Lett.

[3c] Liu C, Deng Y, Wang J, Yang Y, Tang S, Lei A (2011). Angew. Chem.

[4] Erian A, Sherif S, Gaber H (2003). Molecules.

[5a] Marigo M, Jorgensen KA (2006). Chem. Commun.

[5b] Smith AMR, Hii KK (2011). Chem. Rev.

[6a] van der Drift RC, Bouwman E, Drent E (2002). J. Organomet. Chem.

[6b] Uma R, Crévisy C, Grée R (2003). Chem. Rev.

[6c] Mantilli L, Mazet C (2011). Chem. Lett.

[6d] Lorenzo-Luis P, Romerosa A, Serrano-Ruiz M (2012). ACS Catal.

[6e] Bizet V, Pannecoucke X, Renaud J-L, Cahard D (2012). enantiospecific isomerization of allylic alcohols. Angew. Chem.

[7a] Sheppard TD (2011). Synlett.

[7b] Ahlsten N, Bartoszewicz A, Martín-Matute B (2012). Dalton Trans.

[8a] Mizuno A, Kusama H, Iwasawa N (2010). Chem. Eur. J.

[8b] Yang X-F, Wang M, Varma RS, Li C-J (2003). Org. Lett.

[8c] Cuperly D, Petrignet J, Crévisy C, Grée R (2006). Chem. Eur. J.

[8d] Branchadell V, Crévisy C, Grée R (2004). Chem. Eur. J.

[8e] Petrignet J, Prathap I, Chandrasekhar S, Yadav JS, Grée R (2007). Angew. Chem.

[8f] Cao HT, Roisnel T, Valleix A, Grée R (2011). Eur. J. Org. Chem.

[8g] Lin L, Yamamoto K, Matsunaga S, Kanai M (2012). Angew. Chem.

[9a] Bartoszewicz A, Livendahl M, Martín-Matute B (2008). Chem. Eur. J.

[9b] Ahlsten N, Martín-Matute B (2009). Adv. Synth. Catal.

[9c] Ahlsten N, Martín-Matute B (2011). Chem. Commun.

[9d] Ahlsten N, Bartoszewicz A, Agrawal S, Martín-Matute B (2011). Synthesis.

[10] Quintard A, Alexakis A, Mazet C (2011). Angew. Chem.

[11a] Esteruelas MA, Hernández YA, López AM, Oliván M, Rubio L (2008). Organometallics.

[11b] Garralda MA (2009). Dalton Trans.

[12] Batuecas M, Esteruelas MA, García-Yebra C, Oñate E (2010). Organometallics.

[13a] Ogo S, Makihara N, Watanabe Y (1999). Organometallics.

[13b] Nutton A, Bailey PM, Maitlis PM (1981). J. Chem. Soc. Dalton Trans.

[14a] Bellarosa L, Díez J, Gimeno J, Lledós A, Suárez FJ, Ujaque G, Vicent C (2012). Chem. Eur. J.

[14b] Díez J, Gimeno J, Lledós A, Suárezy FJ, Vicent C (2012). ACS Catal.

[15] Tsuji K, Ishikawa H (1994). Bioorg. Med. Chem. Lett.

[16] Haviv F, Ratajczyk JD, DeNet RW, Kerdesky FA, Walters RL, Schmidt SP, Holms JH, Young PR, Carter GW (1988). J. Med. Chem.

[17a] Donohoe TJ, Kabeshov MA, Rathi AH, Smith IED (2012). Org. Biomol. Chem.

[17b] Donohoe TJ, Kabeshov MA, Rathi AH, Smith IED (2010). Synlett.

[17c] Aoyama T, Murata S, Arai I, Araki N, Takido T, Suzuki Y, Kodomari M (2006). Tetrahedron.

