# Supplementary material for: Iridium-Catalyzed 1,3-Hydrogen Shift/Chlorination of Allylic Alcohols
Source: Angew Chem Int Ed Engl. 2013 Apr 24;52(24):6273–6. doi: 10.1002/anie.201301013 (PMC3933732; doi:10.1002/anie.201301013)
Supplement: Supplementary file 1 [file anie0052-6273-sd1.pdf]

Angewandte Chemie
Eine Zeitschrift der Gesellschaft Deutscher Chemiker

Supporting Information

© Wiley-VCH 2013

69451 Weinheim, Germany

Iridium-Catalyzed 1,3-Hydrogen Shift/Chlorination of Allylic Alcohols**

*Nanna Ahlsten, Antonio Bermejo Gómez, and Belén Martín-Matute**

anie_201301013_sm_miscellaneous_information.pdf

Table of contents for Supporting Information

S1 General information

S2 General procedure for the synthesis of allylic alcohols **2a-b, 2c-d, 3j-3o**

S2 General procedure for tandem Ir catalyzed isomerization/C−Cl bond formation

S2 Unsuccessful substrates in tandem Ir catalyzed isomerization/C−Cl bond formation

S2 Multigram scale synthesis of **3c** and **3o**

S3 Figure S1

S4 Experimental procedure for the uptake of data for Figure S1

S4 Deuterium labeling experiment

S5 Characterization data for allylic alcohols **2k** and **2o**

S6 Characterization data for α-chloroketones **3a-3o** and α-chloroaldehydes **7a-7f**

S14 Synthesis and characterization data for 2-aminothiazoles **9-12**

S17 ^{1}H NMR, ^{13}C NMR spectra of α-chloroketone **3f-*d₁***

S18 ^{1}H NMR, ^{13}C NMR spectra of allylic alcohols **2k** and **2o**

S20 ^{1}H NMR, ^{13}C NMR spectra of α-chloroketones **3a-3o** and α-chloroaldehydes **7a-7f**

S41 GC chromatograms of α-chloroketones **rac-3k** and **3k**

S42 ^{1}H NMR, ^{13}C NMR spectra of 2-aminothiazoles **9-12**

S46 References

General Information:

All iridium-catalyzed reactions were carried out in closed glass-vials under an atmosphere of air. Air and moisture sensitive reactions were carried out in oven-dried glassware, under an atmosphere of dry nitrogen. Reagents were used as obtained from commercial suppliers without further purification. THF was used as obtained from supplier (puriss. p. a., stabilized with 2,6-di-tert-butyl-4-methylphenol ~250 mg/l). The undistilled THF used in the reactions tested negative for peroxides (0% by Quantofix peroxide). Flash chromatography was carried out on 60 Å (35-70 µm) silica gel (Acros Kieselgel 60) using pentane, mixtures pentane/EtOAc or pentane/Et_2O as eluent. Analytical TLC was performed on aluminum backed plates (1.5 Å~ 5 cm) pre-coated (0.25 mm) with silica gel (Merck, Silica Gel 60 F254). Compounds were visualized by exposure to UV light or by dipping the plates in a solution of 5% $KMnO_4$ in 95% acidified water (w/v). Melting points were recorded in a metal block and are uncorrected.

^{1}H NMR spectra were recorded at 400 MHz; ^{13}C NMR spectra were recorded at 100 MHz on a Bruker Advance spectrometer. ^{1}H and ^{13}C NMR chemical shifts (*δ*) are reported in

ppm from tetramethylsilane using the residual solvent resonance ($CDCl_3$: δ_H 7.26 and δ_C 77.0). Coupling constants (J) are given in Hz. High resolution mass spectra (HRMS) were recorded on Bruker microTOF ESI-TOF mass spectrometer. NMR yields were calculated using 0.5 equiv. of 1,2,4,5-tetrachloro-3-nitrobenzene as internal standard.

Oct-1-en-3-ol, (*E*)-oct-2-en-1-ol, cinnamyl alcohol and 2-cyclohexen-1-ol were used as obtained from suppliers without further purification. $[Cp^*IrCl_2]_2$ and $[Cp^*Ir(H_2O)_3]SO_4$ were synthesized as described in the literature.[1] Allylic alcohols **2f-g**,[2] **2h**,[3] **6c**,[4] **6d**,[5] **6e**,[6] **6f**[7] were synthesized according to literature procedures.

General procedure for the synthesis of allylic alcohols 2a-b, 3c-d, 3j-30:

Allylic alcohols **2a-b, 3c-d, 3j-3o** were prepared according to a slightly modified literature procedure:[8]

The corresponding aldehyde (1 equiv.) was added dropwise to a solution of vinyl magnesium bromide (1 M in THF, 1.1 equiv.) at 0 °C. The reaction was stirred at this temperature during 1 h, warmed up to room temperature and stirred until consumption of the starting aldehyde (TLC monitoring). The reaction was quenched with NH_4Cl (aq), extracted with EtOAc (3x20 mL) and washed with brine. The combined organic phases were dried over $MgSO_4$ and the solvent was removed under reduced pressure. Purification by column chromatography (EtOAc/Pentane 1:20 – 1:5) afforded the pure allylic alcohols.

General procedure for tandem Ir catalyzed isomerization/C−Cl bonds formation:

$[Cp^*IrCl_2]_2$ (0.25-0.5 mol%) was dissolved in a mixture THF/H_2O (4.8 mL; 1:1 or 1:2). To this mixture was added the desired allylic alcohol (1 mmol, 1 equiv.) and *N*-chlorosuccinimide (160 mg, 1.2 mmol, 1.2 equiv.) and the reaction was stirred at room temperature until consumption of the starting material (TLC monitoring). The mixture was extracted with Et_2O (3x1 mL) and the combined organic layers dried over $MgSO_4$ and concentrated under reduced pressure. The crude mixture was purified by flash silica gel column chromatography (pentane/Et_2O) to give the corresponding α-chlorinated ketone/aldehyde.

Unsuccessful substrates for tandem Ir catalyzed isomerization/C−Cl bonds formation

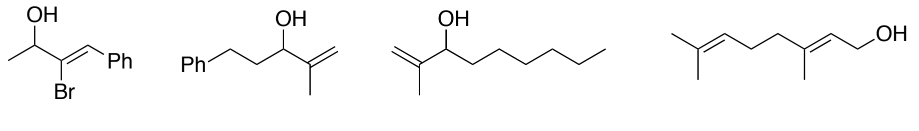

Multigram scale synthesis of 3c:

[Cp^*IrCl_2]$_2$ (156 mg, 0.25 mol%) was dissolved in a mixture THF/H_2O (300 mL; 1:2). 1-Octen-3-ol (10 g, 78 mmol, 1 equiv.) and *N*-chlorosuccinimide (12.5 g, 93.6 mmol, 1.2 equiv.) were added and the reaction mixture was stirred at room temperature for 8 h. The resulting mixture was washed with brine (150 mL) and the aqueous layer extracted with Et_2O (3x100 mL). To the combined organic layers were added 15 g of silica and the mixture was concentrated under vacuum. The supported crude product was purified by flash silica gel column chromatography (using other additional 15 g of silica) and using pentane as eluent to give **3c** as a colorless oil (12.25 g, 97%).

Multigram scale synthesis of 3o

[Cp^*IrCl_2]$_2$ (209 mg, 0.5 mol%) was dissolved in a mixture THF/H_2O (300 mL; 1:1). 1-(4-Isobutylphenyl)prop-2-en-1-ol (10 g, 52.6 mmol, 1 equiv.) and *N*-chlorosuccinimide (8.42 g, 63.1 mmol, 1.2 equiv.) were added and the reaction mixture was stirred at room temperature for 16 h. The corresponding mixture was washed with brine (150 mL) and the aqueous layer extracted with Et_2O (3x100 mL). To the combined organic layers were added 15 g of silica and concentrated. The supported crude mixture was purified by flash silica gel column chromatography (using other additional 15 g of silica) and using pentane as eluent to give **3o** as a colorless oil (9.8 g, 83%).
Anal. Calcd for $C_{13}H_{17}ClO$: C, 69.48; H, 7.62; Cl, 15.78; Found: C, 69.77; H, 7.70; Cl, 15.90.

Figure S1. Reaction profile for isomerization/chlorination of 2a catalyzed by [Cp^*IrCl_2]$_2$ (1) and by [$Cp^*Ir(H_2O)_3$]SO_4 (8)

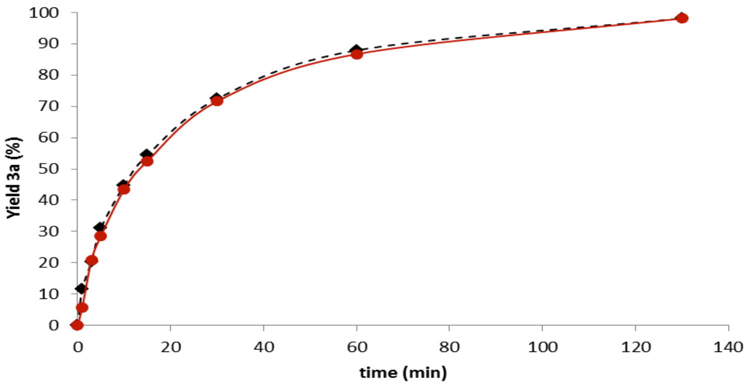

Reaction of **2a** (0.2 mmol) with NCS (0.24 mmol) catalyzed by [Cp^*IrCl_2]$_2$ (**1**) (0.25 mol%) (dashed black) and by [$Cp^*Ir(H_2O)_3$]SO_4 (**8**) (0.5 mol%) (solid red) in THF/H_2O = 1:2.

Experimental procedure for the uptake of data for Figure S1:

Stock solutions of the catalyst percursors were prepared as follows: $[Cp^*IrCl_2]_2$ (0.01 mmol, 8 mg), or $[Cp^*Ir(H_2O)_3]SO_4$ (9.6 mg, 0.02 mmol) were stirred in THF/H_2O (20 mL, v/v 1:2) until fully dissolved (2h for $[Cp^*IrCl_2]_2$ and 10 min for $[Cp^*Ir(H_2O)_3]SO_4$). Alicuots of 1 mL were added to a vial containing **2a** (32.4 mg, 0.2 mmol) and NCS (32 mg, 0.24 mmol), and the mixture was immediately stirred at the maximum speed. The reactions were quenched at the appropriate time (3 min, 5 min, 10 min, 15 min, 30 min, 1h and 2h) by very quickly adding dietyl ether (2 mL) and extracting the resulting dietlyether/THF phase. This phase was dried over $MgSO_4$ and the conversion of **2a** and yield of **3a** was determined by ^{1}H NMR spectroscopy. Control experiments in which the organic phase was allowed to stand for an extended time showed no differences in conversion/yield.

Deuterium labeling experiments:

Deuterium labeled allylic alcohol 2-d-(*E*)-4-phenylbut-3-en-2-ol (**2f-*d₁***) (96% deuterium) (0.5 mmol) was chlorinated according to the general procedure using 0.25 mol% of $[Cp^*IrCl_2]_2$ and THF/H_2O (4.8 mL; 1:2) as solvent (15 h reaction time). After purification by column chromatography (pentane/EtOAc, 50:1) **3f-d_1** was obtained as a colorless oil. Analysis by ^{1}H, ^{13}C and ESI-HRMS showed a deuterium content of 96% in **3f-d_1**.

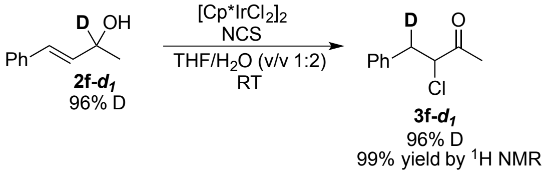

4-d-3-Chloro-4-phenylbutan-2-one (3f-d_1).

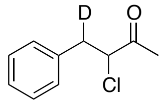

^{1}H NMR ($CDCl_3$, 400 MHz, mixture of 2 diastereomers (1:1)): δ 7.34-7.27 (m, 6H), 7.25-7.21 (m, 4H), 4.40 (d, J_{HH} = 7.9 Hz, 2H), 3.32 (dt, J_{HH} = 6.0 Hz, J_{HD} = 1.86 Hz, 1H), 3.1 (dt, J_{HH} = 7.9 Hz, J_{HD} = 1.9 Hz, 1H), 2.29 (s, 3H). 2.28 (s, 3H); ^{13}C NMR ($CDCl_3$, 100 MHz, mixture of diastereomers): δ 202.6, 136.1, 129.3, 128.6, 127.2, 63.7, 39.5 (t, J_{CD} = 19.8 Hz), 39.4 (t, J_{CD} = 20.3 Hz), 26.8; HRMS (ESI) Exact mass calcd for $C_{10}H_{10}DOClNa$ $[M+Na]^+$: 206.0453, found: 206.0460.

Crossover experiment:

A mixture of deuterium labeled allylic alcohol **2f-*d₁*** (0.25 mmol) and allylic alcohol **2c** (0.25 mmol) were chlorinated according to the General Procedure using 0.25 mol% of $[Cp^*IrCl_2]_2$ and THF/H_2O (4.8 mL; 1:2) as solvent (15 h reaction time). Analysis of the product mixture by ^1H, ^{13}C NMR and ESI-HRMS showed no **4-d-3-Chloro-4-phenylbutan-2-one** (**3c-*d*$_1$**) and a 96% deuterium content of **3f-*d*$_1$**.

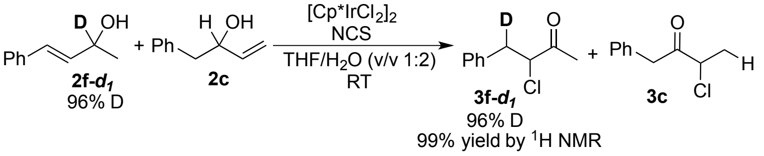

Characterization data for allylic alcohols 2k and 2o:

1-((*R*)-1,4-dioxaspiro[4.5]decan-2-yl)prop-2-en-1-ol (2k)

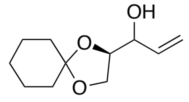

The title compound was prepared according to the general procedure from (*R*)-1,4-dioxaspiro[4.5]decane-2-carbaldehyde.[9] Purification by column chromatography afforded **1k** as a colorless oil (73 % isolated yield as a mixture of 2 diastereomers (dr = 3:2))

$[\alpha]^{25}_D$ +2.2 (*c* 0.75, $CHCl_3$)

^1H NMR ($CDCl_3$, 400 MHz, mixture of 2 diastereomers): δ 5.87-5.76 (m, 2H), 5.41-5.36 (m, 2H), 5.26-5.22 (m, 2H), 4.35-4.30 (m, 1H), 4.14-3.76 (m, 7H), 2.39 (d, J_{HH} = 2.8 Hz, 1H), 2.3 (d, J_{HH} = 3.0 Hz, 1H), 1.68-1.50 (m, 16H), 1.49-1.30 (m, 4H); ^{13}C NMR ($CDCl_3$, 125 MHz, mixture of diastereomers): δ 136.4, 135.8, 118.0, 117.0, 110.6, 110.1, 78.4, 77.8, 74.5, 71.9, 65.7, 64.3, 36.6, 36.3, 34.9 34.8, 25.29, 25.26, 24.2, 24.1, 23.9 (2C); HRMS (ESI) Exact mass calcd for $C_{11}H_{18}O_3Na$ $[M+Na]^+$: 221.1148, found: 221.1147.

1-(4-isobutylphenyl)prop-2-en-1-ol (2o)

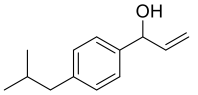

The title compound was prepared according to the general procedure from 4-Isobutylbenzaldehyde. Purification by column chromatography (pentane/EtOAc 10:1) afforded

1p as a colorless oil (95% yield)

^{1}H NMR (400 MHz, $CDCl_3$): δ 7.29 (d, J_{HH} = 8.0 Hz, 2H), 7.15 (d, J_{HH} = 8.0 Hz, 2H), 6.08 (ddd, J_{HH} = 17.1 Hz, J_{HH} = 10.3 Hz, J_{HH} = 6.0 Hz, 1H), (dt, J_{HH} = 17.1 Hz, 1.3 Hz, 1H), 5.23-5.17 (m, 2H), 2.49 (d, J_{HH} = 7.3 Hz, 2H), 2.01 (br s, 1H), 1.88 (m, 1H), 0.92 (d, J_{HH} = 6.5 Hz, 6H); ^{13}C NMR ($CDCl_3$, 125 MHz): δ 141.3, 140.3, 139.9, 129.3 (2C), 126.1 (2C), 114.8, 75.2, 45.1, 30.2, 22.34 (2C).

Characterization data for α-chloroketones 3a-3o and α-chloroaldehydes 7a-7f:

4-Chloro-1-phenylpentan-3-one (3a)

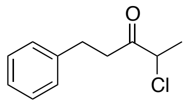

The title compound was prepared according the General Procedure from 5-phenylpent-1-en-3-ol (**2a**) (162 mg) using 0.25 mol% of $[Cp^*IrCl_2]_2$ and THF/H_2O (4.8 mL; 1:2) as solvent (6 h reaction time). Purification by column chromatography (pentane/Et_2O, 30:1) afforded **3a** as a colorless oil (174 mg, 88%).

^{1}H NMR (400 MHz, $CDCl_3$): δ 7.34-7.28 (m, 2H), 7.25-7.22 (m, 3H), 4.32 (q, J_{HH} = 6.9 Hz, 1H), 3.15-3.05 (m, 1H), 3.01-2.94 (m, 3H), 1.59 (d, J_{HH} = 6.9 Hz, 3H); ^{13}C NMR (100 MHz, $CDCl_3$): δ 204.6, 140.6, 128.5, 128.3, 126.2, 58.6, 40.0, 29.8, 20.0; HRMS (ESI) Exact mass calcd for $C_{11}H_{13}OClNa$ $[M+Na]^+$: 219.0547, found: 219.0549.

1-(Benzyloxy)-3-chlorobutan-2-one (3b).

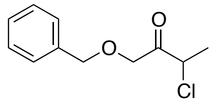

The title compound was prepared according the General Procedure from 1-(benzyloxy)but-3-en-2-ol (**2b**) (178 mg) using 0.25 mol% of $[Cp^*IrCl_2]_2$ and THF/H_2O (4.8 mL; 1:2) as solvent (5 h reaction time) Purification by column chromatography (pentane/Et_2O, 20:1) afforded **3b** as a colorless oil (189 mg, 89%).

^{1}H NMR (400 MHz, $CDCl_3$): *δ* 7.38-7.30 (m, 5H), 4.62 (s, 2H), 4.57 (q, J_{HH} = 6.9 Hz, 1H), 4.43-4.39 (m, 2H), 1.61 (d, J_{HH} = 6.9 Hz, 3H); ^{13}C NMR (100 MHz, $CDCl_3$): δ 202.6, 137.1, 128.7, 128.7, 128.2, 73.7, 72.3, 55.2, 19.8; HRMS (ESI) Exact mass calcd for $C_{11}H_{13}O_2ClNa$ $[M+Na]^+$: 235.0496, found: 235.0508.

3-Chloro-1-phenylbutan-2-one (3c).

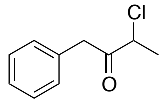

The title compound was prepared according the General Procedure from 1-phenylbut-3-en-2-ol (**2c**) (740 mg, 5 mmol) and *N*-chlorosuccinimide (800 mg, 6 mmol) using 0.25 mol% of $[Cp^*IrCl_2]_2$ and THF/H_2O (24 mL; 1:2) as solvent (15 h reaction time). Purification by column chromatography (pentane/EtOAc, 50:1) afforded **3c** as a colorless oil (822 mg, 90%).

^1H NMR (400 MHz, $CDCl_3$): δ 7.39-7.25 (m, 5H), 4.45 (q, J_{HH} = 6.9 Hz, 1H), 4.06-3.96 (m, 2H), 1.61 (d, J_{HH} = 6.9 Hz, 3H); ^{13}C NMR (100 MHz, $CDCl_3$): δ 202.3, 133.4, 129.5, 128.7, 127.2, 57.4, 45.3, 19.9; HRMS (ESI) Exact mass calcd for $C_{10}H_{11}ClONa$ $[M+Na]^+$: 205.0391, found: 205.0401.

2-Chloro-4-ethyloctan-3-one (3d).

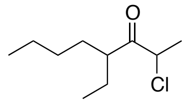

The title compound was prepared according the General Procedure from 4-ethyloct-1-en-3-ol (**2d**) (156 mg) using 0.25 mol% of $[Cp^*IrCl_2]_2$ and THF/H_2O (4.8 mL; 1:1) as solvent (16 h reaction time). Purification by column chromatography (pentane/Et_2O, 30:1) afforded **3d** as a colorless oil (160 mg, 84%).

^1H NMR (400 MHz, $CDCl_3$, mixture of 2 diastereomers): δ 4.40 (q, J_{HH} = 6.8 Hz, 1H), 2.88-2.80 (m, 1H), 1.75-1.37 (m, 4H), 1.58 (d, J_{HH} = 6.8 Hz, 3H), 1.34-1.19 (m, 4H), 0.90-0.85 (m, 6H); ^{13}C NMR (100 MHz, $CDCl_3$, mixture of 2 diastereomers) δ 208.01, 207.97, 57.69, 57.67, 50.0, 49.8, 31.7, 30.6, 29.6, 29.5 25.2, 24.4, 22.79, 22.77, 19.7, 19.6, 13.87, 13.85, 11.9, 11.7; HRMS (ESI) Exact mass calcd for $C_{10}H_{19}ClONa$ $[M+Na]^+$: 213.1017, found: 213.1017.

2-Chlorooctan-3-one (3e).

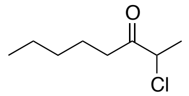

The title compound was prepared according the General Procedure from oct-1-en-3-ol (**2e**) (128 mg) using 0.25 mol% of $[Cp^*IrCl_2]_2$ and THF/H_2O (4.8 mL; 1:2) as solvent (2 h reaction time). Purification by column chromatography (pentane/EtOAc, 100:1) afforded **3e** as a colorless oil (148 mg, 91%).

^1H NMR (400 MHz, $CDCl_3$): δ 4.32 (q, J_{HH} = 6.9 Hz, 1H), 2.74-2.56 (m, 2H), 1.64-1.56

(m, 2H), 1.58 (d, J_{HH} = 6.9 Hz, 3H), 1.35-1.24 (m, 4H), 0.89 (t, J_{HH} = 7.0 Hz, 3H); ^{13}C NMR (100 MHz, $CDCl_3$): δ 205.6, 58.5, 38.2, 31.2, 23.4, 22.4, 20.1, 13.9; HRMS (ESI) Exact mass calcd for $C_8H_{16}OCl$ $[M+H]^+$: 163.0884, found: 163.0888.

3-Chloro-4-phenylbutan-2-one (3f).

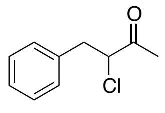

The title compound was prepared according the General Procedure from (*E*)-4-phenylbut-3-en-2-ol (**2f**) (740 mg, 5 mmol) and *N*-chlorosuccinimide (800 mg, 6 mmol) using 0.25 mol% of $[Cp^*IrCl_2]_2$ and THF/H_2O (24 mL; 1:2) as solvent (15 h reaction time). Purification by column chromatography (pentane/EtOAc, 50:1) afforded **3f** as a colorless oil (834 mg, 91%).

^{1}H NMR (400 MHz, $CDCl_3$): δ 7.34-7.21 (m, 5H), 4.41 (dd, J_{HH} = 8.1 Hz, J_{HH} = 6.3 Hz, 1H), 3.34 (dd, J_{HH} = 14.4 Hz, J_{HH} = 6.3 Hz, 1H), 3.08 (dd, J_{HH} = 14.7 Hz, J_{HH} = 8.1 Hz, 1H), 2.29 (s, 3H); ^{13}C NMR (100 MHz, $CDCl_3$): δ 202.6, 136.1, 129.3, 128.6, 127.2, 63.8, 39.8, 26.8; HRMS (ESI) Exact mass calcd for $C_{10}H_{11}ClONa$ $[M+Na]^+$: 205.0391, found: 205.0382.

3-Chloro-4-(*p*-tolyl)butan-2-one (3g).

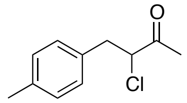

The title compound was prepared according the General Procedure from (*E*)-4-(*p*-tolyl)but-3-en-2-ol (**2g**) (162 mg) using 0.25 mol% of $[Cp^*IrCl_2]_2$ and THF/H_2O (4.8 mL; 1:2) as solvent (5 h reaction time). Purification by column chromatography (pentane/Et_2O 30:1) afforded **3g** as a colorless oil (165.2 mg, 84%).

^{1}H NMR (400 MHz, $CDCl_3$): δ 7.14-7.09 (m, 4H), 4.38 (dd, J_{HH} = 8.0 Hz, J_{HH} = 6.3 Hz, 1H), 3.29 (dd, J_{HH} = 14.3 Hz, J_{HH} = 6.3 Hz, 1H), 3.04 (dd, J_{HH} = 14.3 Hz, J_{HH} = 8.0 Hz, 1H), 2.33 (s, 3H), 2.28 (s, 3H); ^{13}C NMR (100 MHz, $CDCl_3$): δ 202.9, 137.0, 133.2, 129.4, 129.3, 64.1, 39.6, 26.9, 21.2; HRMS (ESI) Exact mass calcd for $C_{11}H_{13}OClNa$ $[M+Na]^+$: 219.0547, found: 219.0552.

2-Chloro-1-phenylpentan-3-one (3h).

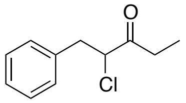

The title compound was prepared according the General Procedure from (*E*)-1-phenylpent-1-en-3-ol (**2h**) (162 mg) using 0.5 mol% of $[Cp^*IrCl_2]_2$ and THF/H_2O (4.8 mL; 1:2) as solvent (20 h reaction time) Purification by column chromatography (pentane/EtOAc, 50:1) afforded **3h** as a colorless oil (175 mg, 89%).

^{1}H NMR (400 MHz, $CDCl_3$): δ 7.33-7.20 (m, 5H), 4.43 (dd, J_{HH} = 7.9 Hz, J_{HH} = 6.5 Hz, 1H), 3.34 (dd, J_{HH} = 14.2 Hz, J_{HH} = 6.5 Hz, 1H), 3.09 (dd, J_{HH} = 14.2 Hz, J_{HH} = 7.9 Hz, 1H), 2.72-2.62 (m, 1H), 2.57-2.47 (m, 1H), 1.05 (t, J_{HH} = 7.26 Hz, 3H); ^{13}C NMR (100 MHz, $CDCl_3$): δ 205.5, 136.3, 129.4, 128.6, 127.1, 62.9, 39.9, 33.1, 7.6; HRMS (ESI) Exact mass calcd for $C_{11}H_{13}ClONa$ $[M+Na]^+$: 219.0547, found: 219.0552.

2-Chlorocyclohexanone (3i).

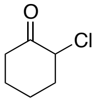

The title compound was prepared according the General Procedure from cyclohexen-1-ol (**2i**) (49 mg, 0.5 mmol) and *N*-chlorosuccinimide (80 mg, 0.6 mmol) using 0.5 mol% of $[Cp^*IrCl_2]_2$ and THF/H_2O (4.8 mL; 1:2) as solvent (24 h reaction time). The crude of **3i** was obtained in 92% yield as determined by ^{1}H NMR against an internal standard.

2-Chloro-5,9-dimethyldec-8-en-3-one (3j).

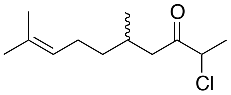

The title compound was prepared according the General Procedure from 5,9-dimethyldeca-1,8-dien-3-ol (**2j**) (182 mg) using 0.25 mol% of $[Cp^*IrCl_2]_2$ and THF/H_2O (4.8 mL; 1:1) as solvent. After 18 h and purification by column chromatography (pentane/EtOAC, 80:1) afforded **3j** as a pale yellow oil (162.5 mg, 75%).

^{1}H NMR (400 MHz, $CDCl_3$, mixture of 2 diastereomers): δ 5.10-5.07 (m, 1H), 4.33-4.27 (m, 1H), 2.70-2.44 (m, 2H), 2.10-1.92 (m, 3H), 1.68 (m, 3H), 1.60-1.58 (m, 6H), 1.37-1.18 (m, 2H), 0.93-0.90 (m, 3H); ^{13}C NMR (100 MHz, $CDCl_3$, mixture of 2 diastereomers): δ 205.1, 205.0, 131.6, 131.6, 124.2, 124.2, 58.8, 58.5, 45.6, 36.9, 36.8, 28.7, 28.6, 25.7, 25.5, 25.5, 20.1, 20.0, 19.7, 19.6, 17.7; HRMS (ESI) Exact mass calcd for $C_{12}H_{21}OClNa$ $[M+Na]^+$: 239.1173,

found: 239.1171.

2-Chloro-1-((*R*)-1,4-dioxaspiro[4.5]decan-2-yl)propan-1-one (3k).

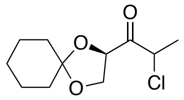

The title compound was prepared according the General Procedure from 1-(1,4-dioxaspiro[4.5]decan-2-yl)prop-2-en-1-ol (**2k**) (198 mg) using 0.5 mol% of $[Cp^*IrCl_2]_2$ and THF/H_2O (4.8 mL; 1:1) as solvent. After 24 h and purification by column chromatography (pentane/Et_2O, 10:1) afforded **3k** as a colorless oil in 99% ee (176.7 mg, 89%).

$[\alpha]^{25}_D$ +107.7 (*c* 0.94, $CHCl_3$)

^1H NMR ($CDCl_3$, 400 MHz, mixture of 2 diastereomers (≈ 2:1)): δ 4.89-4.82 (m, 3H), 4.73-4.70 (dd, J_{HH} = 7.8 Hz, J_{HH} = 5.8 Hz, 1H), 4.30-4.26 (m, 1H), 4.21 (dd, J_{HH} = 8.8 Hz, J_{HH} = 7.4 Hz, 1H), 4.14 (dd, J_{HH} = 8.8 Hz, J_{HH} = 5.0 Hz, 1H), 4.08 (dd, J_{HH} = 8.8 Hz, J_{HH} = 5.8 Hz, 1H), 1.80-1.52 (m, 22H), 1.50-1.35 (m, 4H); ^{13}C NMR ($CDCl_3$, 125 MHz, mixture of diastereomers): δ 204.3, 203.6, 112.3, 111.8, 78.2, 77.5, 67.0, 66.1, 55.1, 53.5, 35.8, 35.6, 34.6, 34.5, 25.1 (2C), 24.1 (2C), 23.91, 23.89, 20.5, 19.1; HRMS (ESI) Exact mass calcd for $C_{11}H_{17}O_3ClNa$ $[M+Na]^+$: 255.0758, found: 255.0747. GC (CP-Chirasil-Dex CD, 100 °C to 180 °C (3 °C/min)): t_R 19.3 and 20.1 min (minor, two diastereomers) and 19.8 and 20.7 min (major, two diastereomers).

2-Chloro-1-phenylpropan-1-one (3l)

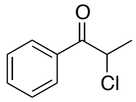

The title compound was prepared according the General Procedure from 1-phenylprop-2-en-1-ol (**2l**) (134 mg) using 0.5 mol% of $[Cp^*IrCl_2]_2$ and THF/H_2O (4.8 mL; 1:1) as solvent (20 h reaction time) Purification by column chromatography (pentane/Et_2O, 30:1) afforded **3l** as a colorless oil (134.9 mg, 80%).

^1H NMR (400 MHz, $CDCl_3$): δ 8.03-8.01 (m, 2H), 7.63-7.58 8m, 1H), 7.52-7.48 (m, 2H), 5.25 (q, J_{HH} = 6.7 Hz, 1H), 1.75 (d, J_{HH} = 6.7 Hz, 3H); ^{13}C NMR (100 MHz, $CDCl_3$): δ 193.8, 134.3, 133.9, 129.1 (2C), 128.9 (2C), 52.9, 20.1; HRMS (ESI) Exact mass calcd for C_9H_9OClNa $[M+Na]^+$: 191.0234, found: 191.0243.

2-Chloro-1-(4-chlorophenyl)propan-1-one (3m)

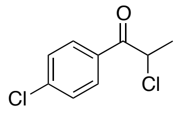

The title compound was prepared according the General Procedure from 1-(4-chlorophenyl)prop-2-en-1-ol (**2m**) (134.2 mg) using 0.5 mol% of $[Cp^*IrCl_2]_2$ and THF/H_2O (4.8 mL; 1:1) as solvent. After 24 h and purification by column chromatography (pentane/Et_2O, 30:1) afforded **3m** as a white solid (162.4 mg, 80%). m.p. 56-58 °C.

^{1}H NMR (400 MHz, $CDCl_3$) δ 8.0-7.96 (m, 2H), 7.50-7.46 (m, 2H), 5.19 (q, J_{HH} = 6.7 Hz, 1H), 1.75 (d, J_{HH} = 6.7 Hz, 3H); ^{13}C NMR (100 MHz, $CDCl_3$) δ 192.4, 140.2, 132.4, 130.4 (2C), 129.1 (2C), 52.6, 19.8; HRMS (ESI) Exact mass calcd for $C_9H_8OCl_2Na$ $[M+Na]^+$: 224.9844, found: 224.9837.

2-Chloro-1-(3-methoxyphenyl)propan-1-one (3n)

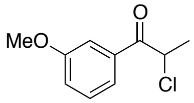

The title compound was prepared according the General Procedure from 1-(3-methoxyphenyl)prop-2-en-1-ol (**2n**) (164 mg) using 0.5 mol% of $[Cp^*IrCl_2]_2$ and THF/H_2O (4.8 mL; 1:1) as solvent (24 h reaction time). Purification by column chromatography (pentane/Et_2O, 30:1) afforded **3n** as a pale yellow solid (184.7 mg, 93%). m.p. 68-69 °C.

^{1}H NMR (400 MHz, $CDCl_3$): δ 7.60-7.58 (m, 1H), 7.54-7.53 (m, 1H), 7.40 (t, J_{HH} = 8.0 Hz, 1H), 7.16-7.13 (m, 1H), 5.23 (q, J_{HH} = 6.7 Hz, 1H), 3.86 (s, 3H), 1.74 (d, J_{HH} = 6.7 Hz, 3H); ^{13}C NMR (100 MHz, $CDCl_3$): δ 193.6, 160.1, 135.6, 129.9, 121.6, 120.4, 113.5, 55.6, 53.0, 20.2; HRMS (ESI) Exact mass calcd for $C_{10}H_{11}O_2ClNa$ $[M+Na]^+$: 221.0340, found: 221.0350.

2-Chloro-1-(4-isobutylphenyl)propan-1-one (3o)

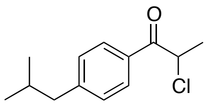

The title compound was prepared according the General Procedure from 1-(4-isobutylphenyl)prop-2-en-1-ol (**2o**) (190.3 mg) using 0.5 mol% of $[Cp^*IrCl_2]_2$ and THF/H_2O (4.8 mL; 1:1) as solvent (16 h reaction time) Purification by column chromatography (pentane/Et_2O, 30:1) afforded **30** as a white solid (171 mg, 76%). m.p. 55-56 °C.

^{1}H NMR (400 MHz, $CDCl_3$) δ 7.96-7.93 (m, 2H), 7.29-7.25 (m, 2H), 5.25 (q, J_{HH} = 6.8

Hz, 1H), 2.56 (d, J_{HH} = 7.3 Hz, 2H), 1.99-1.87 (m, 1H), 1.75 (d, J_{HH} = 6.6 Hz, 3H), 0.93 (d, J_{HH} = 6.8, 6H); ^{13}C NMR (100 MHz, $CDCl_3$) δ 193.3, 148.4, 131.8, 129.4 (2C), 129.0 (2C), 52.8, 45.4, 30.1, 22.3, 20.0; HRMS (ESI) Exact mass calcd for $C_{13}H_{17}ClONa$ $[M+Na]^+$: 247.0860, found: 247.0864; Anal. Calcd for $C_{13}H_{17}ClO$: C, 69.48; H, 7.62; Cl, 15.78; Found: C, 69.69; H, 7.89; Cl, 15.74.

2-Chlorooctanal (7a)

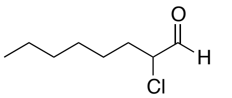

The title compound was prepared according the General Procedure from (*E*)-oct-2-en-1-ol (**6a**) (163 mg) using 0.5 mol% of $[Cp^*IrCl_2]_2$ and THF/H_2O (4.8 mL; 1:1) as solvent. After 20 h and purification by column chromatography (pentane/Et_2O, 30:1) afforded **7a** as a colorless oil (105.7 mg, 65%).

^{1}H NMR (400 MHz, $CDCl_3$): δ 9.48 (d, J_{HH} = 2.5 Hz, 1H), 4.15 (ddd, J_{HH} = 8.1 Hz, J_{HH} = 5.4 Hz, J_{HH} = 2.5 Hz, 1H), 2.03-1.93 (m, 1H), 1.87-1.77 (m, 1H), 1.56-1.24 (m, 8H), 0.88 (t, J_{HH} = 6.8 Hz, 3H); ^{13}C NMR (100 MHz, $CDCl_3$): δ 195.5, 64.1, 32.2, 31.6, 28.7, 25.6, 22.6, 14.1; HRMS (ESI) Exact mass calcd for $C_8H_{16}OCl$ $[M+H]^+$: 163.0884, found: 163.0888.

2-Chloro-3-phenylpropanal (7b)

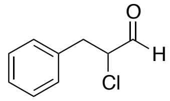

The title compound was prepared according the General Procedure from cinnamyl alcohol (**6b**) (134 mg) using 0.5 mol% of $[Cp^*IrCl_2]_2$ and THF/H_2O (4.8 mL; 1:1) as solvent. After 23 h and purification by column chromatography (pentane/EtOAC, 3:1) afforded **7b** as a white solid (120 mg, 71%). m.p. 85-87 °C.

^{1}H NMR (400 MHz, $CDCl_3$): δ 9.57 (d, J_{HH} = 2.1 Hz, 1H), 7.38-7.25 (m, 5H), 4.41 (ddd, J_{HH} = 8.1 Hz, J_{HH} = 5.7 Hz, J_{HH} = 2.1 Hz, 1H), 3.41 (dd, J_{HH} = 14.4 Hz, J_{HH} = 5.7 Hz, 1H), 3.11 (dd, J_{HH} = 14.4 Hz, J_{HH} = 8.1 Hz, 1H); ^{13}C NMR (100 MHz, $CDCl_3$): δ 194.4, 135.4, 129.4, 128.7, 127.4, 63.9, 38.3; HRMS (ESI) Exact mass calcd for C_9H_9OClNa $[M+Na]^+$: 191.0234, found: 191.0242.

2-Chloro-5-phenylpentanal (7c)

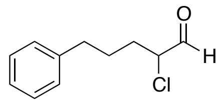

The title compound was prepared according the General Procedure from (*E*)-5-phenylpent-2-en-1-ol (**6c**) (162 mg) using 0.25 mol% of $[Cp^*IrCl_2]_2$ and THF/H_2O (4.8 mL; 1:1) as solvent. After 5.5 h and purification by column chromatography (pentane/EtOAc, 10:1) afforded **7c** as a colorless oil (179 mg, 91%).

^{1}H NMR (400 MHz, $CDCl_3$): δ 9.50 (d, J_{HH} = 2.32 Hz, 1H), 7.34-7.30 (m, 2H), 7.24-7.19 (m, 3H), 4.19 (ddd, J_{HH} = 7.6 Hz, J_{HH} = 5.2 Hz, J_{HH} = 2.3 Hz, 1H), 2.71-2.68 (m, 2H), 2.06-2.01 (m, 1H), 1.95-1.77 (m, 3H); ^{13}C NMR (100 MHz, $CDCl_3$): δ 195.1, 141.2, 128.5, 128.3, 126.1, 63.8, 35.1, 31.5, 27.2; HRMS (ESI) Exact mass calcd for $C_{11}H_{13}OClNa$ $[M+Na]^+$: 219.0547, found: 219.0542.

2-Chloro-3-(4-chlorophenyl)propanal (7d)

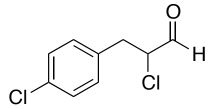

The title compound was prepared according the General Procedure from (*E*)-3-(4-chlorophenyl)prop-2-en-1-ol (**6d**) (168.5 mg) using 0.5 mol% of $[Cp^*IrCl_2]_2$ and THF/H_2O (4.8 mL; 1:1) as solvent. After 21 h and purification by column chromatography (pentane/EtOAc, 4:1) afforded **7d** as a light yellow solid (135.4 mg, 67%). m.p. 65-67 °C.

^{1}H NMR (400 MHz, $CDCl_3$): δ 9.56 (d, J_{HH} = 1.9 Hz, 1H), 7.33 (d, J_{HH} = 8.4 Hz, 2H), 7.19 (d, J_{HH} = 8.4 Hz, 2H), 4.37 (ddd, J_{HH} = 7.4 Hz, J_{HH} = 5.4 Hz, J_{HH} = 1.9 Hz, 1H), 3.37 (dd, J_{HH} = 14.6 Hz, J_{HH} = 5.4 Hz, 1H), 3.07 (dd, J_{HH} = 14.6 Hz, J_{HH} = 7.4 Hz, 1H); ^{13}C NMR (100 MHz, $CDCl_3$): δ 194.2, 133.9, 133.4, 130.8, 128.8, 63.7, 37.5; HRMS (ESI) Exact mass calcd for $C_9H_9OCl_2$ $[M+H]^+$: 203.0025, found: 203.0030.

2-Chloro-3-(4-methoxyphenyl)propanal (7e)

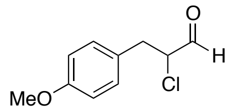

The title compound was prepared according the General Procedure from (*E*)-3-(4-methoxyphenyl)prop-2-en-1-ol (**6e**) (164 mg) using 0.5 mol% of $[Cp^*IrCl_2]_2$ and THF/H_2O (4.8 mL; 1:1) as solvent. After 21 h and purification by column chromatography (pentane/EtOAc, 3:1)

afforded **7e** as a white solid (118 mg, 60%). m.p. 84-85 °C.

^{1}H NMR (400 MHz, $CDCl_3$): δ 9.52 (d, J_{HH} = 2.3 Hz, 1H), 7.15 (d, J_{HH} = 8.6 Hz, 2H), 6.86 (d, J_{HH} = 8.6 Hz, 2H), 4.34 (ddd, J_{HH} = 8.1 Hz, J_{HH} = 5.6 Hz, J_{HH} = 2.3 Hz, 1H), 3.80 (s, 3H), 3.30 (dd, J_{HH} = 14.8 Hz, J_{HH} = 5.8 Hz, 1H), 3.05 (dd, J_{HH} = 14.8 Hz, J_{HH} = 8.1 Hz, 1H); ^{13}C NMR (100 MHz, $CDCl_3$): δ 194.6, 158.9, 130.5, 127.2, 114.1, 64.1, 55.2, 37.6; HRMS (ESI) Exact mass calcd for $C_{10}H_{12}O_2Cl$ $[M+H]^+$: 199.0520, found: 199.0515.

4-(Benzyloxy)-2-chlorobutanal (7f)

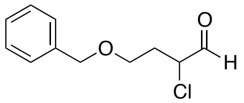

The title compound was prepared according the General Procedure from (*Z*)-4-(benzyloxy)but-2-en-1-ol (**6f**) (178 mg) using 0.5 mol% of $[Cp^*IrCl_2]_2$ and THF/H_2O (4.8 mL; 1:1) as solvent. After 36 h and purification by column chromatography (pentane/EtOAC, 5:1) afforded **7f** as a colorless oil (155 mg, 73%).

^{1}H NMR (400 MHz, $CDCl_3$): δ 9.55 (d, J_{HH} = 1.9 Hz, 1H), 7.40-7.30 (m, 5H), 4.53 (s, 2H), 4.47 (ddd, J_{HH} = 7.3 Hz, J_{HH} = 5.3 Hz, J_{HH} = 1.9 Hz, 1H), 3.75-3.64 (m, 2H), 2.40-2.32 (m, 1H), 2.19-2.11 (m, 1H); ^{13}C NMR (100 MHz, $CDCl_3$): δ 194.9, 137.8, 128.4, 127.8, 127.7, 73.2, 65.2, 61.4, 32.9; HRMS (ESI) Exact mass calcd for $C_{11}H_{13}ClO_2Na$ $[M+Na]^+$: 235.0496, found: 235.0499.

<u>Synthesis of 2-aminothiazoles 9-12 from α-chloroketones 3f, 3e, 3o and 3c:</u>

5-Benzyl-4-methyl-2-aminothiazole (9)

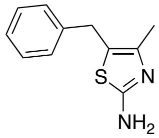

3-Chloro-4-phenylbutan-2-one (**3f**) (365 mg, 2 mmol) and thiourea (160 mg, 2.10 mmol, 1.05 equiv.) were dissolved in EtOH (2 mL) and heated in a pressure tube at 110 °C overnight. The crude mixture was neutralized with $NaHCO_3$ (sat.), extracted with CH_2Cl_2 (x2) and the organic combined organic layers were dried over $MgSO_4$, concentrated under reduced pressure and the resulting solid was washed with pentane (x2) afforded **9** as a palid solid (404 mg, 99%). m.p. 113-115 °C.

^{1}H NMR (400 MHz, $CDCl_3$): δ 7.34-7.20 (m, 5H), 4.74 (bs, 2H), 3.93 (s, 2H), 2.22 (s, 3H); ^{13}C NMR (100 MHz, $CDCl_3$): δ 164.8, 143.5, 140.3, 128.5, 128.1, 126.4, 119.9, 32.1, 14.7;

HRMS (ESI) Exact mass calcd for $C_{11}H_{13}N_2S$ $[M+H]^+$: 205.0794, found: 205.0800; Anal. Calcd for $C_{11}H_{12}N_2S$: C, 64.67; H, 5.92; N, 13.71; S, 15.70; Found: C, 64.68; H, 5.97; N, 13.60; S, 15.86.

4-Benzyl-5-methyl-2-aminothiazole (10)

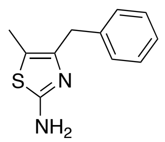

3-Chloro-1-phenylbutan-2-one (**3e**) (365 mg, 2 mmol) and thiourea (160 mg, 2.10 mmol, 1.05 equiv.) were dissolved in EtOH (2 mL) and heated in a pressure tube at 110 °C overnight. The crude mixture was neutralized with $NaHCO_3$ (sat.), extracted with CH_2Cl_2 (x2) and the organic combined organic layers were dried over $MgSO_4$, concentrated under reduced pressure and the resulting solid was washed with pentane (x2) afforded **10** as a white solid (388 mg, 95%). m.p. 76-77 °C.

^{1}H NMR (400 MHz, $CDCl_3$): δ 7.31-7.20 (m, 5H), 4.75 (bs, 2H), 3.84 (s, 2H), 2.27 (s, 3H); ^{13}C NMR (100 MHz, $CDCl_3$): δ 164.3, 145.5, 139.8, 128.5, 128.4, 126.0, 116.6, 34.7, 11.1; HRMS (ESI) Exact mass calcd for $C_{11}H_{13}N_2S$ $[M+H]^+$: 205.0794, found: 205.0798; Anal. Calcd for $C_{11}H_{12}N_2S$: C, 64.67; H, 5.92; N, 13.71; S, 15.70; Found: C, 64.84; H, 5.98; N, 13.66; S, 15.94.

4-(4′-Isobutylphenyl)-5-methyl-2-aminothiazole (11)

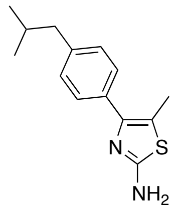

2-Chloro-1-(4-isobutylphenyl)propan-1-one (**3o**) (449 mg, 2 mmol) and thiourea (160 mg, 2.1 mmol, 1.05 equiv.) were dissolved in EtOH (2 mL) and heated in a pressure tube at 110 °C overnight. The crude mixture was neutralized with $NaHCO_3$ (sat.), extracted with CH_2Cl_2 (x2) and the organic combined organic layers were dried over $MgSO_4$, concentrated under reduced pressure and the resulting red oil was dissolved in pentane and evaporated (x3) until appear a yellow solid. The solid was washed with pentane (x2) afforded **11** as a light yellow solid (486 mg, 99%). m.p. 98-99 °C.

^{1}H NMR (400 MHz, $CDCl_3$): δ 7.46 (d, J_{HH} = 8.3 Hz, 2H), 7.17 (d, J_{HH} = 8.3 Hz, 2H),

4.93 (bs, 2H), 2.49 (d, J_{HH} = 7.2 Hz, 2H), 2.39 (s, 3H), 1.88 (h, J_{HH} = 6.7 Hz, 1H), 0.91 (d, J_{HH} = 6.6 Hz, 6H), ^{13}C NMR (100 MHz, $CDCl_3$): δ 163.7, 146.2, 140.7, 132.6, 128.9, 128.0, 116.9, 45.2, 30.2, 22.3, 12.4; HRMS (ESI) Exact mass calcd for $C_{14}H_{19}N_2S$ $[M+H]^+$: 247.1263, found: 247.1269.

5-Methyl-4-pentyl-2-aminothiazole (12)

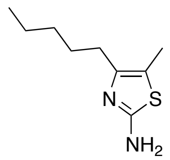

2-Chlorooctan-3-one (**3c**) (325 mg, 2 mmol) and thiourea (160 mg, 2.1 mmol, 1.05 equiv.) were dissolved in EtOH (2 mL) and heated in a pressure tube at 110 °C overnight. The crude mixture was neutralized with $NaHCO_3$ (sat.), extracted with CH_2Cl_2 (x2) and the organic combined organic layers were dried over $MgSO_4$, concentrated under reduced pressure afforded **12** as a yellow oil (359 mg, 98%).

^{1}H NMR (400 MHz, $CDCl_3$): δ 4.72 (bs, 2H), 2.42 (t, J_{HH} = 7.5 Hz, 2H), 2.18 (s, 3H), 1.62-1.55 (m, 2H), 1.36-1.25 (m, 4H), 0.90 (t, J_{HH} = 6.9 Hz, 3H); ^{13}C NMR (100 MHz, $CDCl_3$): δ 163.8, 147.6, 115.2, 31.6, 29.1, 28.7, 22.5, 14.0, 10.9; HRMS (ESI) Exact mass calcd for $C_9H_{17}N_2S$ $[M+H]^+$: 185.1107, found: 185.1115.

4-d-3-Chloro-4-phenylbutan-2-one (3f-d_1).

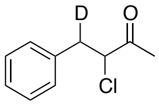

^{1}H NMR (400 MHz, $CDCl_3$)

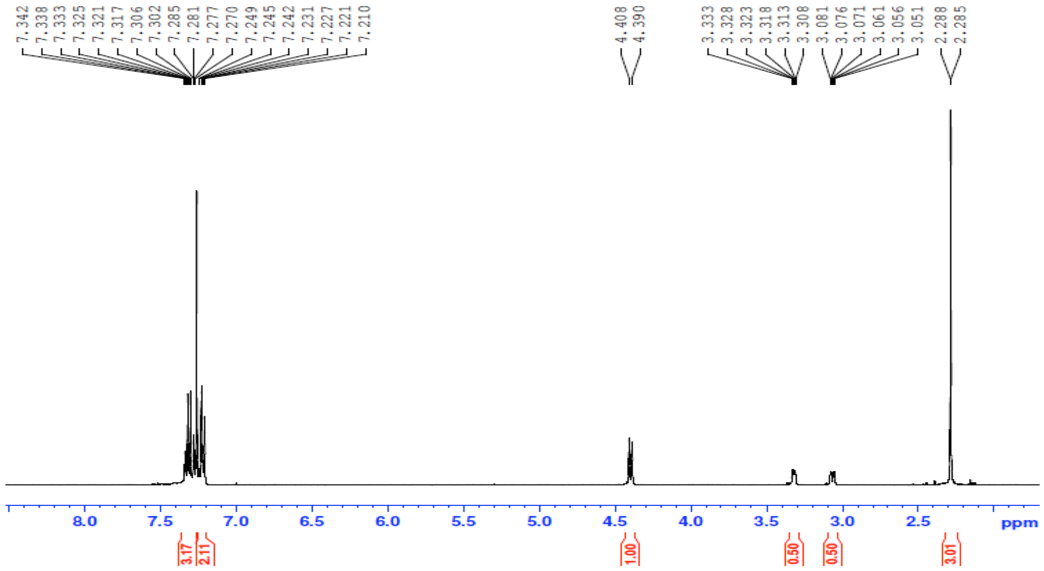

^{13}C NMR (100 MHz, $CDCl_3$)

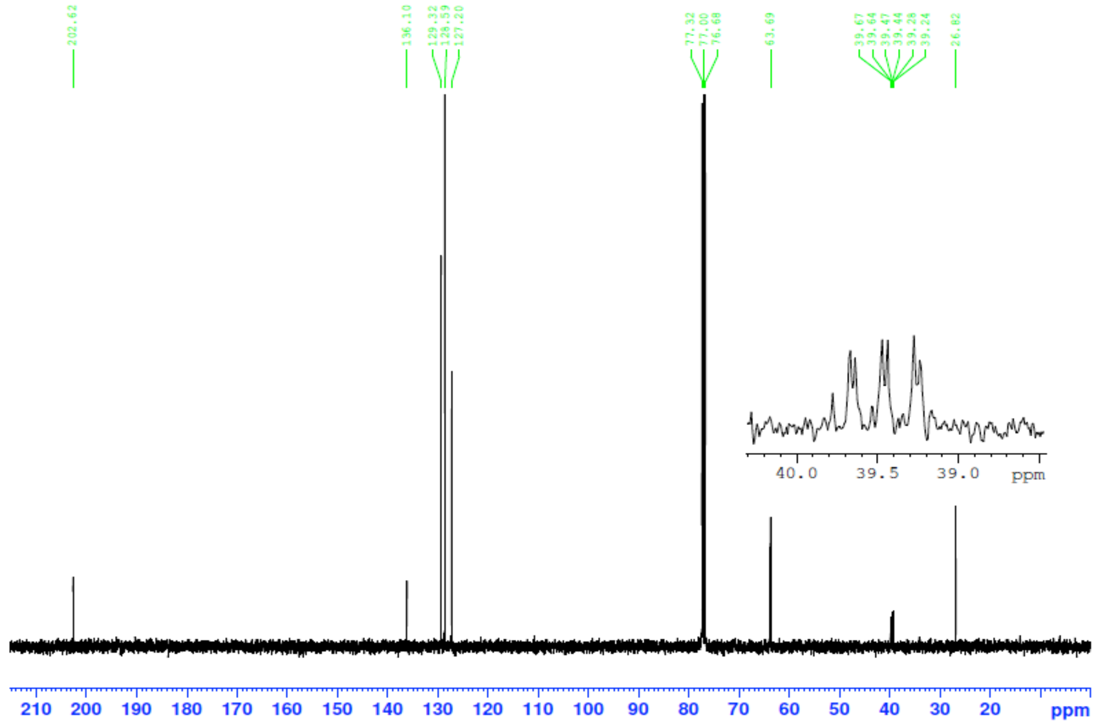

1-((*R*)-1,4-dioxaspiro[4.5]decan-2-yl)prop-2-en-1-ol (2k)

^{1}H NMR (400 MHz, $CDCl_3$)

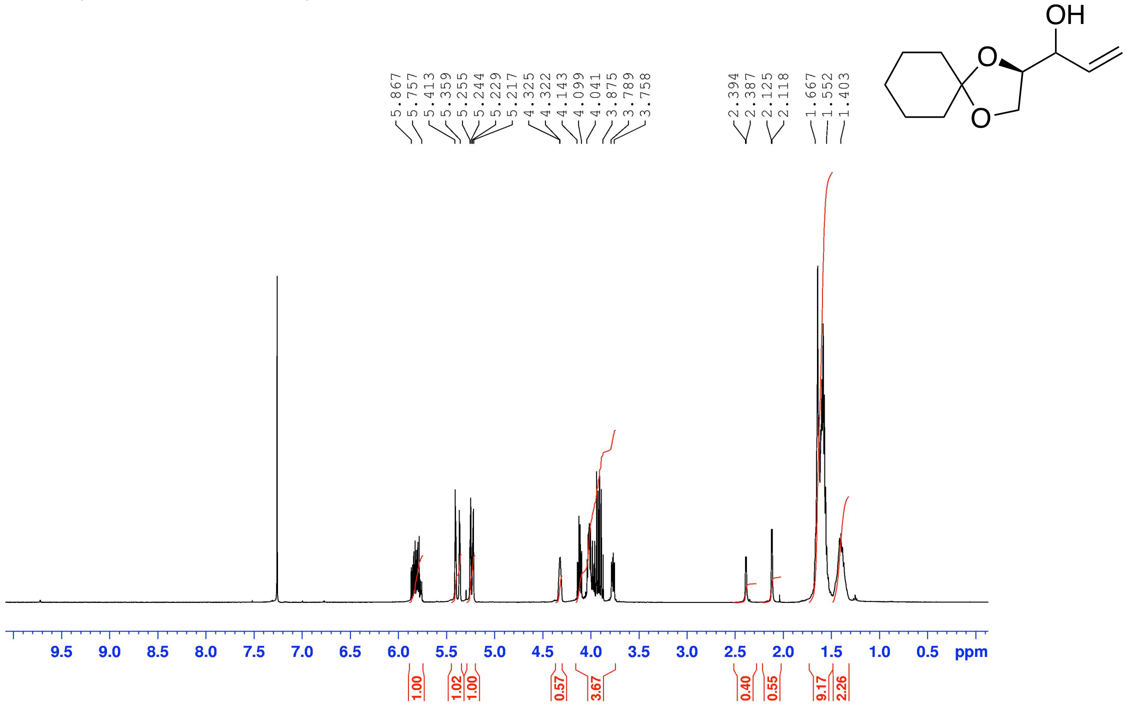

^{13}C NMR (100 MHz, $CDCl_3$)

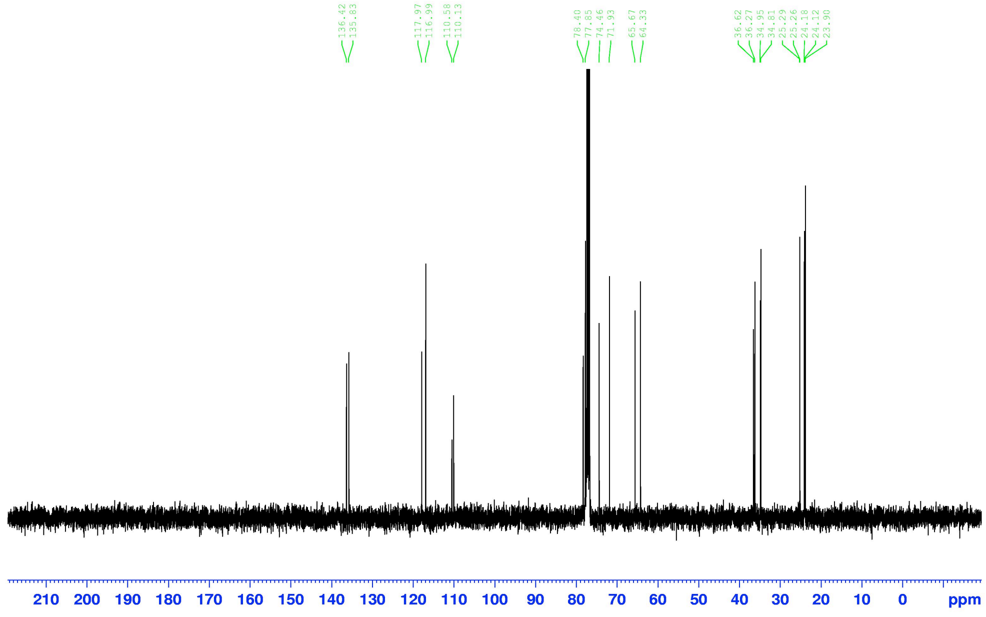

1-(4-isobutylphenyl)prop-2-en-1-ol (2o)

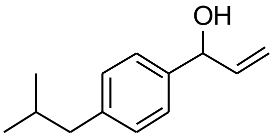

^{1}H NMR (400 MHz, $CDCl_3$)

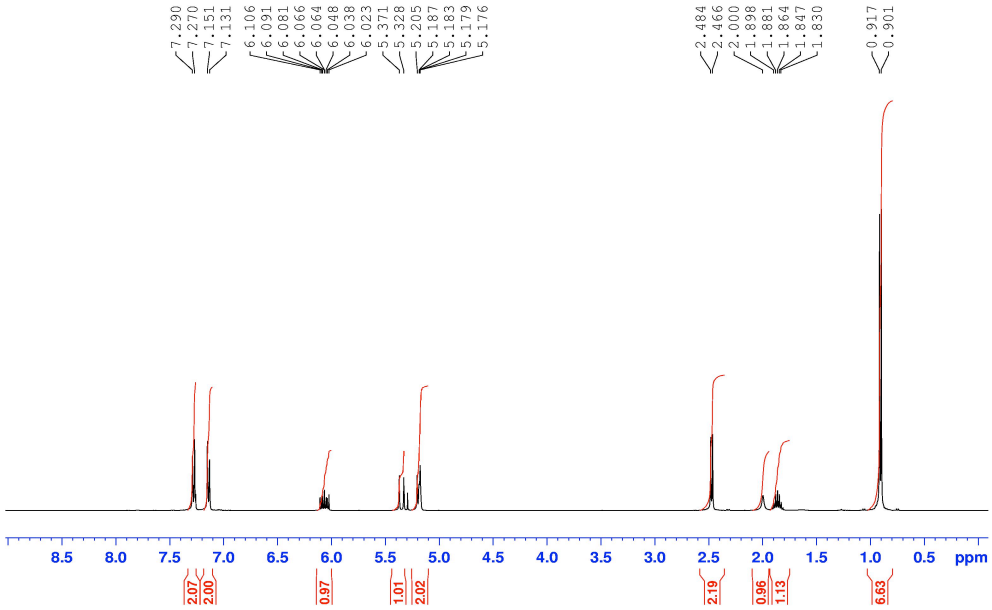

^{13}C NMR (100 MHz, $CDCl_3$)

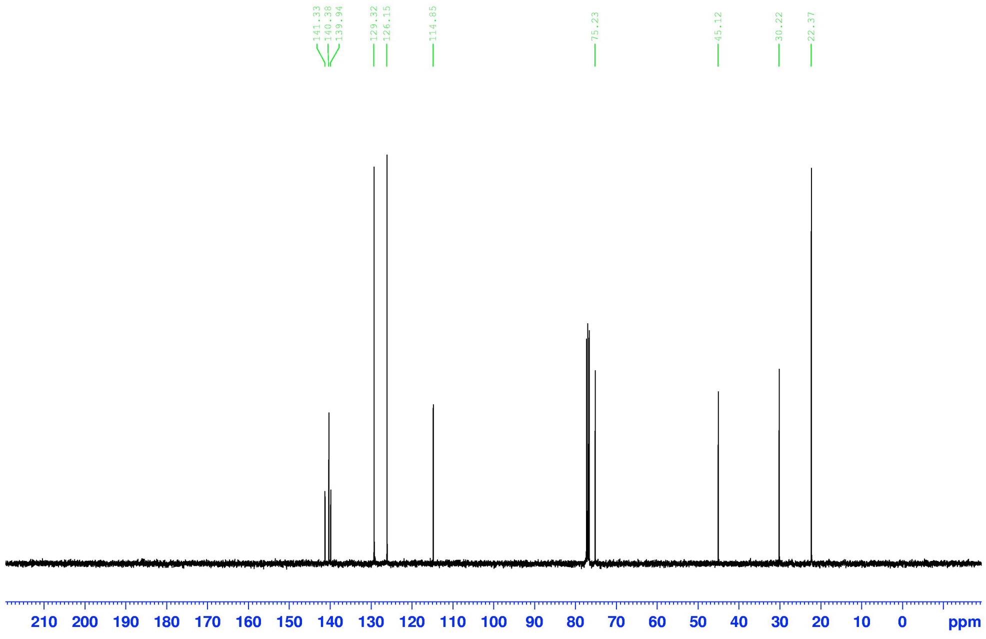

4-Chloro-1-phenylpentan-3-one (3a)

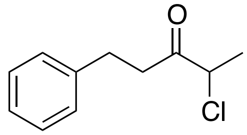

^{1}H NMR (400 MHz, $CDCl_3$)

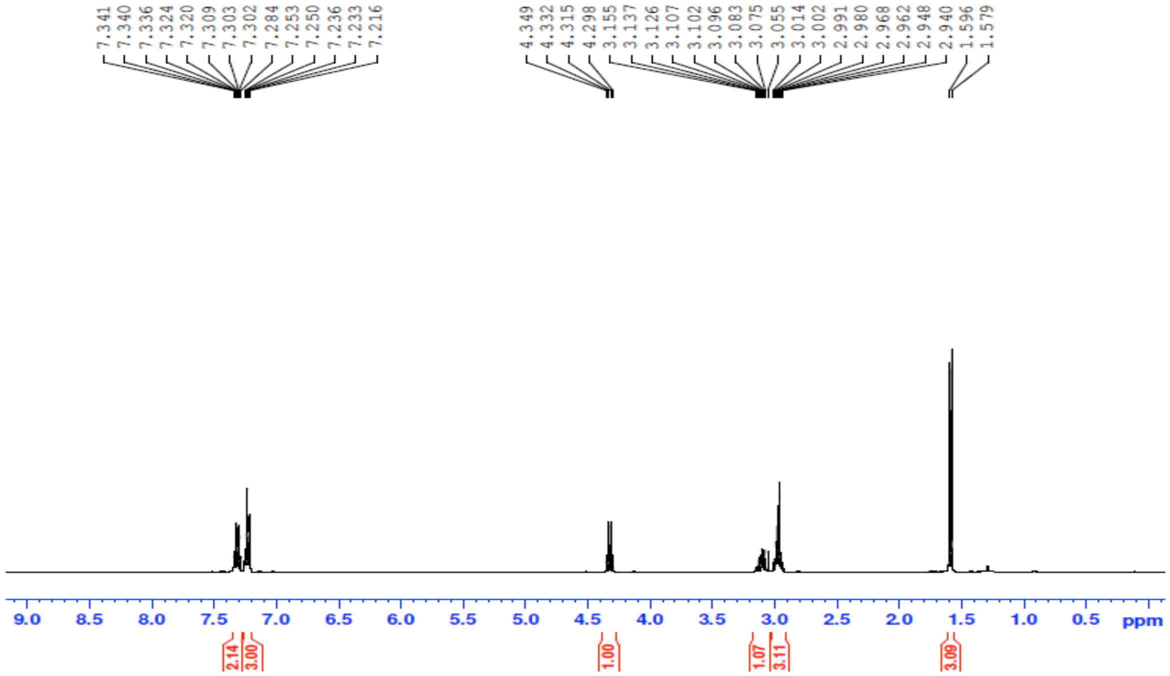

^{13}C NMR (100 MHz, $CDCl_3$)

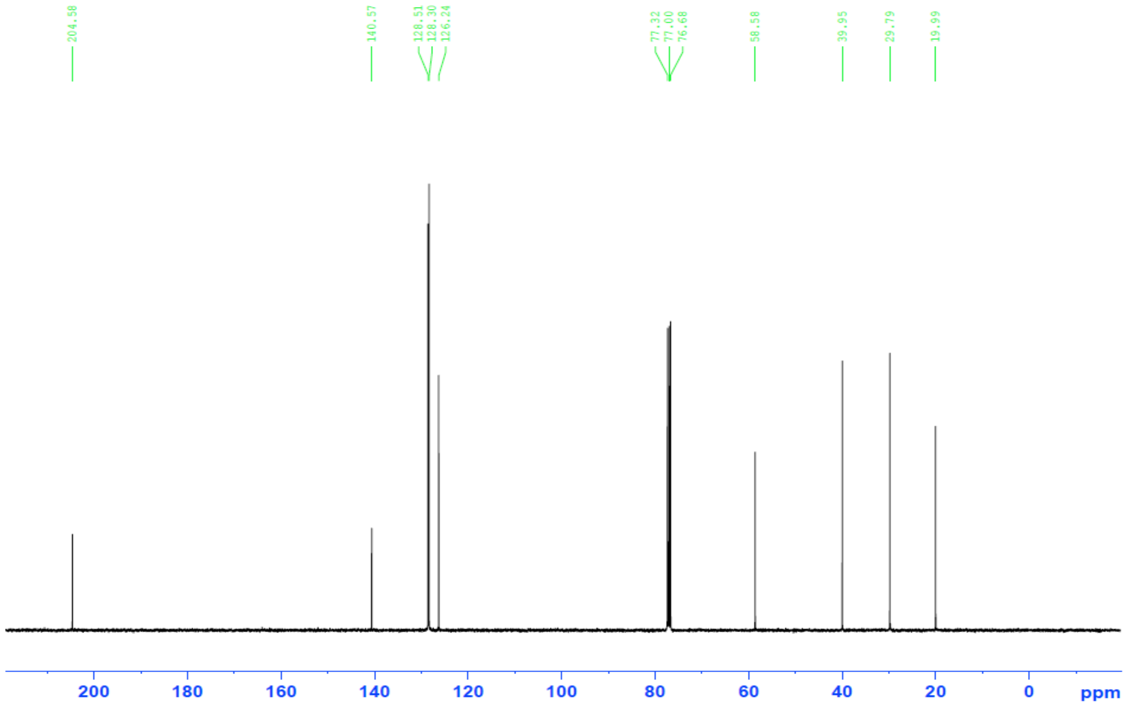

1-(Benzyloxy)-3-chlorobutan-2-one (3b)

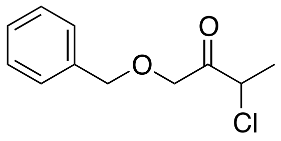

^{1}H NMR (400 MHz, $CDCl_3$)

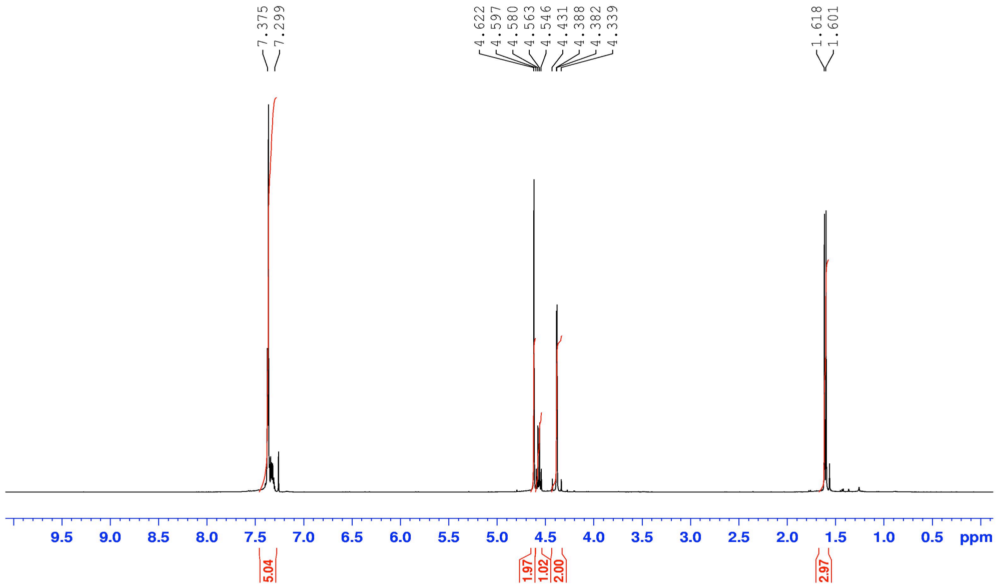

^{13}C NMR (100 MHz, $CDCl_3$)

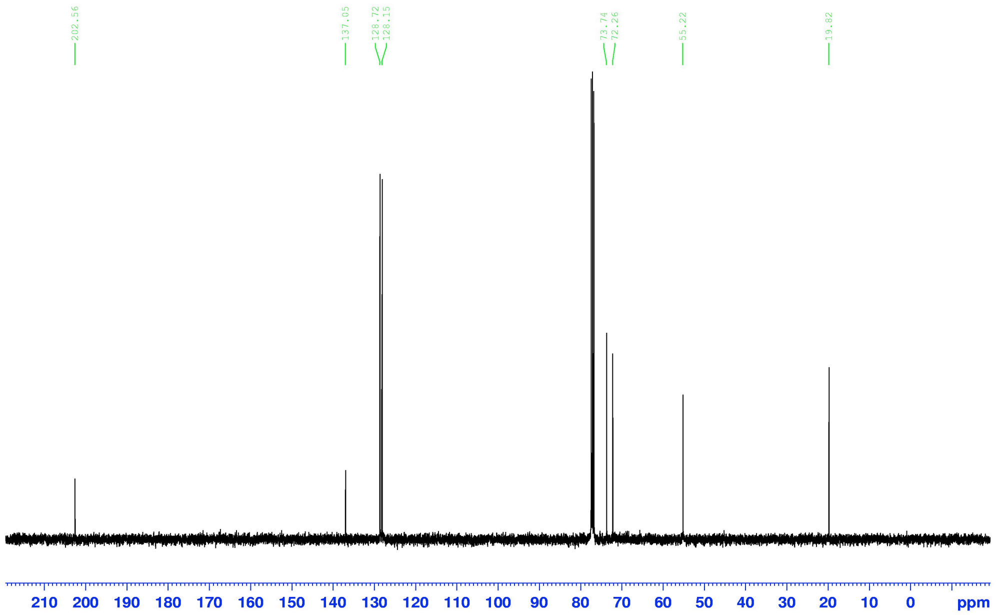

3-Chloro-1-phenylbutan-2-one (3c)

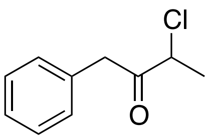

^{1}H NMR (400 MHz, $CDCl_3$)

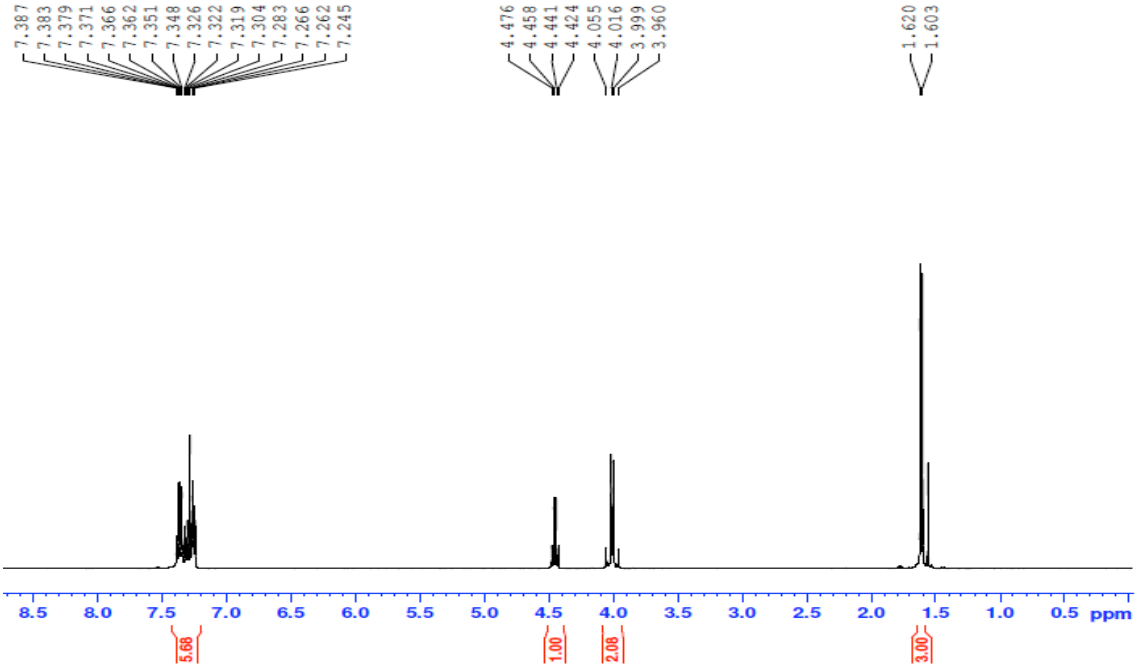

^{13}C NMR (100 MHz, $CDCl_3$)

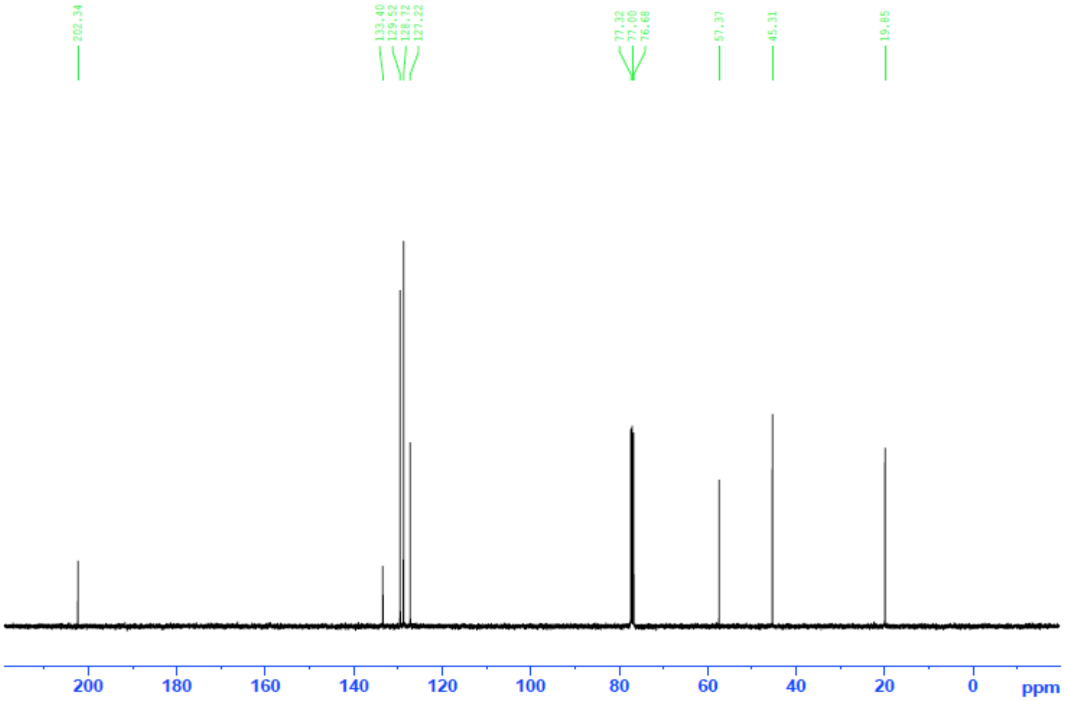

2-Chloro-4-ethyloctan-3-one (3d)

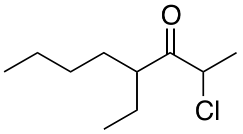

^{1}H NMR (400 MHz, $CDCl_3$)

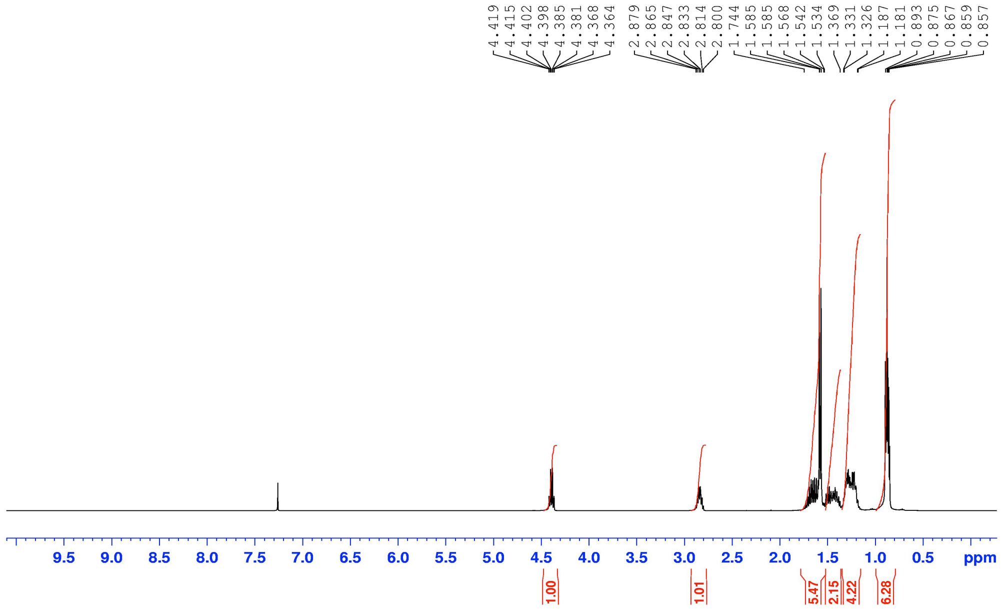

^{13}C NMR (100 MHz, $CDCl_3$)

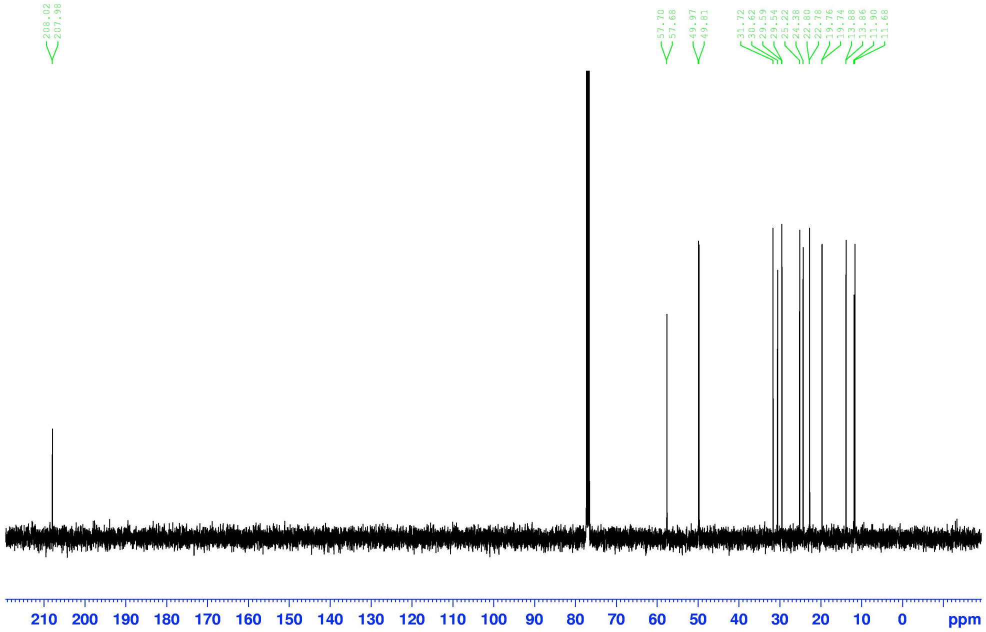

2-Chlorooctan-3-one (3e)

^{1}H NMR (400 MHz, $CDCl_3$)

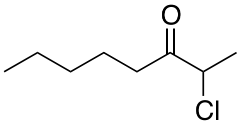

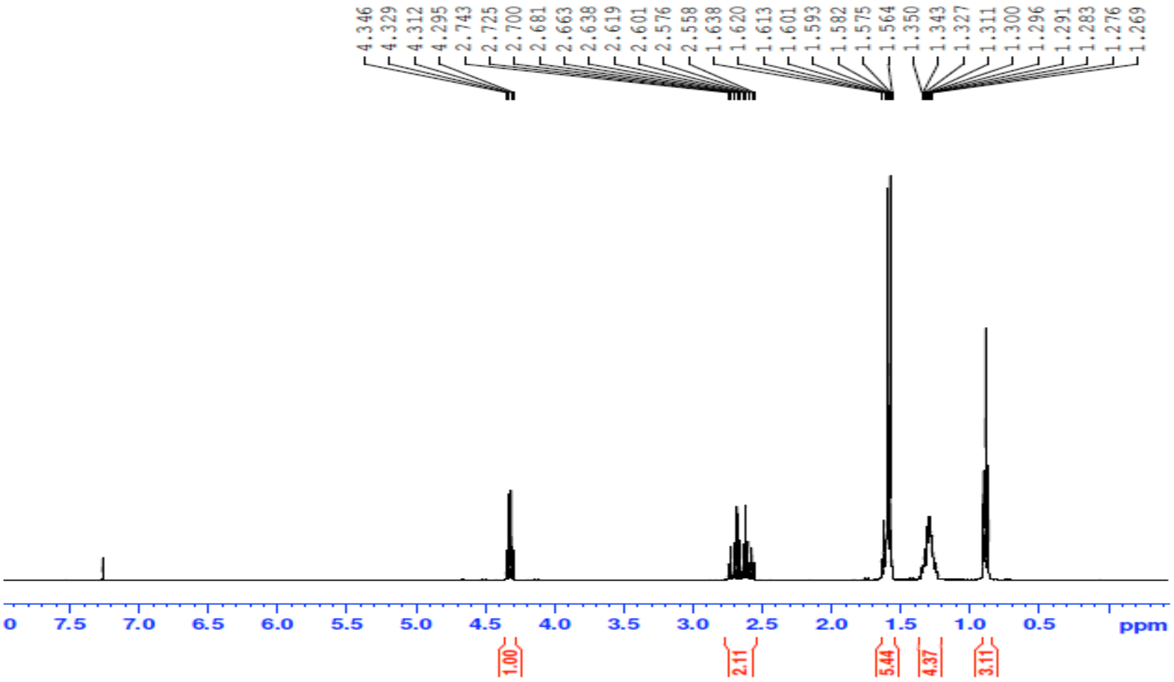

^{13}C NMR (100 MHz, $CDCl_3$)

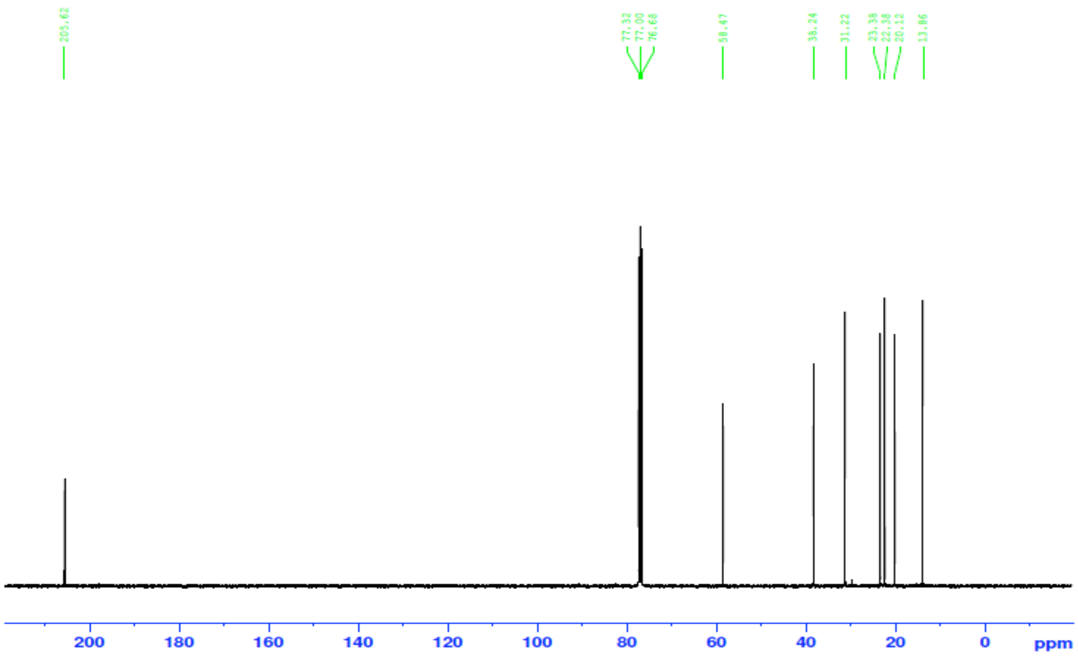

3-Chloro-4-phenylbutan-2-one (3f)

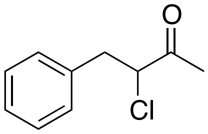

^{1}H NMR (400 MHz, $CDCl_3$)

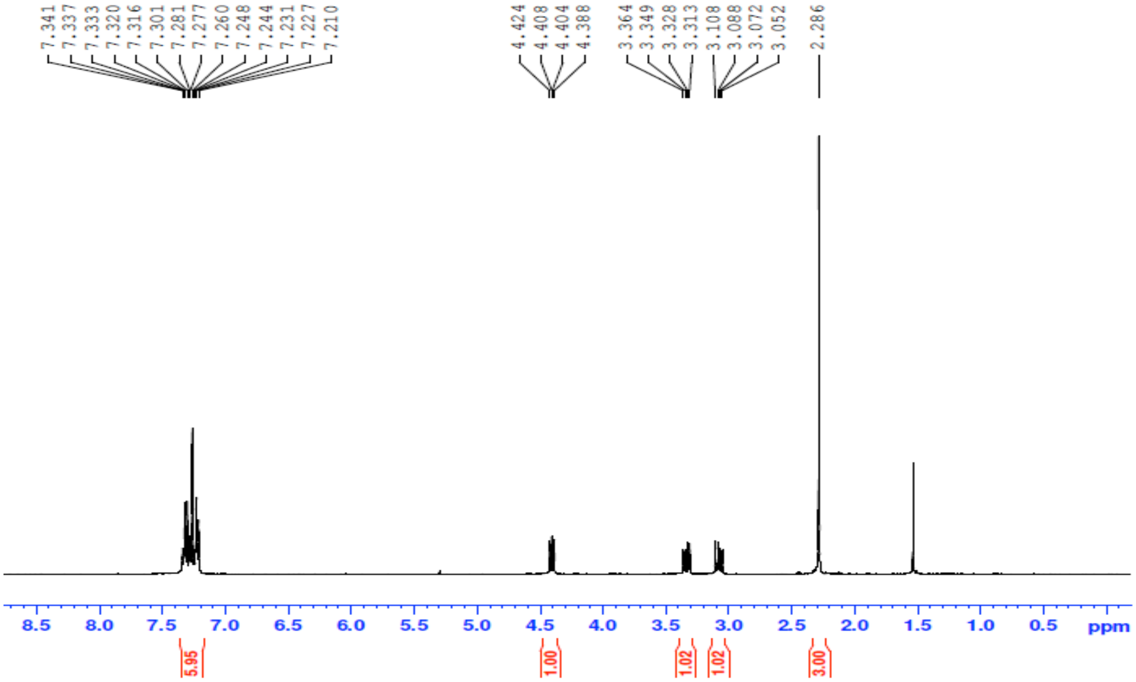

^{13}C NMR (100 MHz, $CDCl_3$)

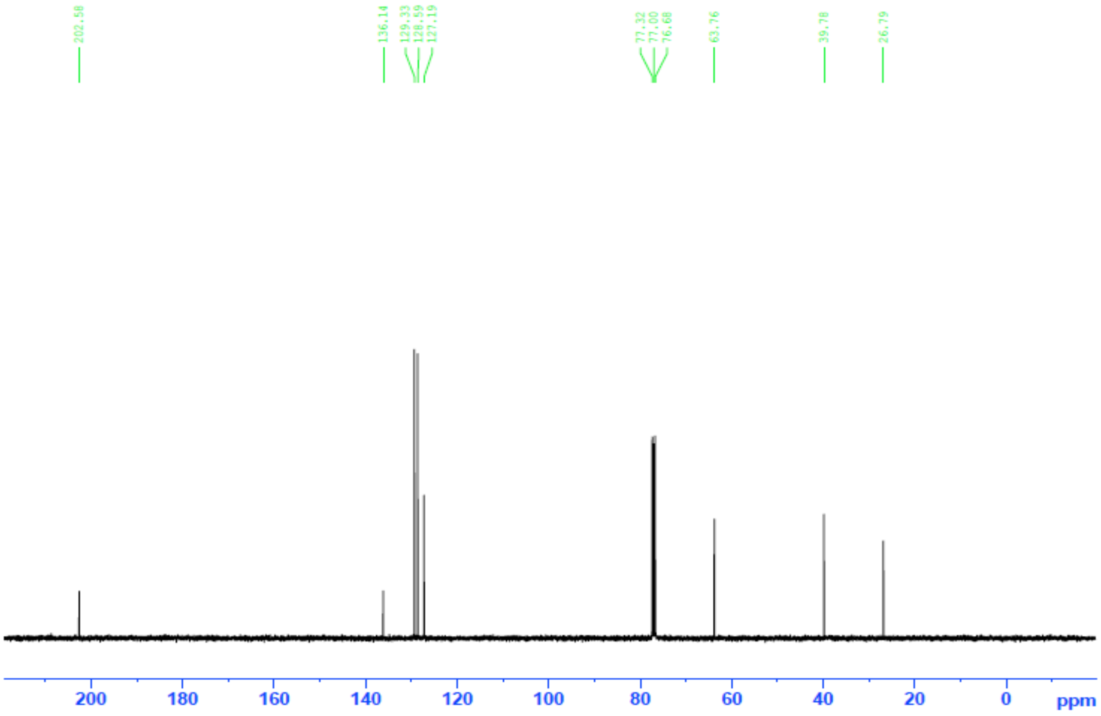

3-Chloro-4-(*p*-tolyl)butan-2-one (3g)

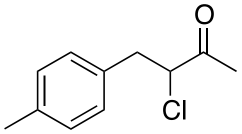

^{1}H NMR (400 MHz, $CDCl_3$)

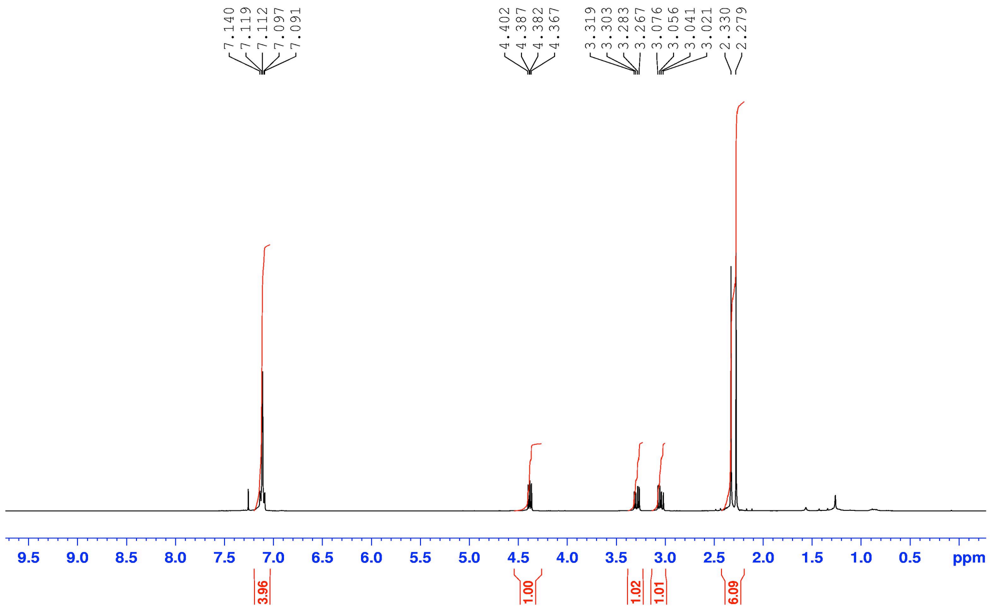

^{13}C NMR (100 MHz, $CDCl_3$)

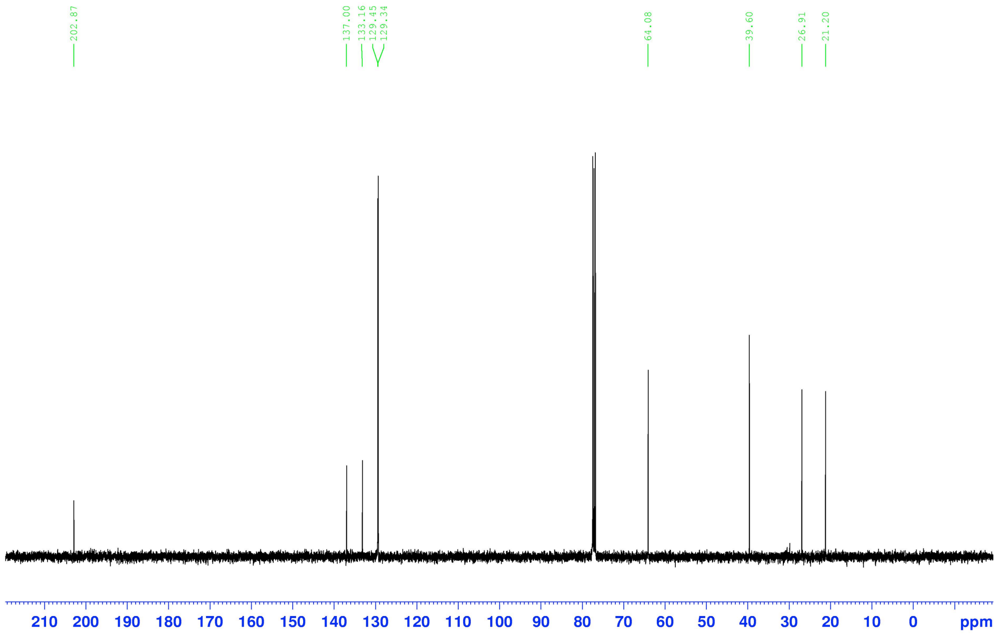

2-Chloro-1-phenylpentan-3-one (3h)

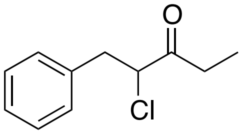

^{1}H NMR (400 MHz, $CDCl_3$)

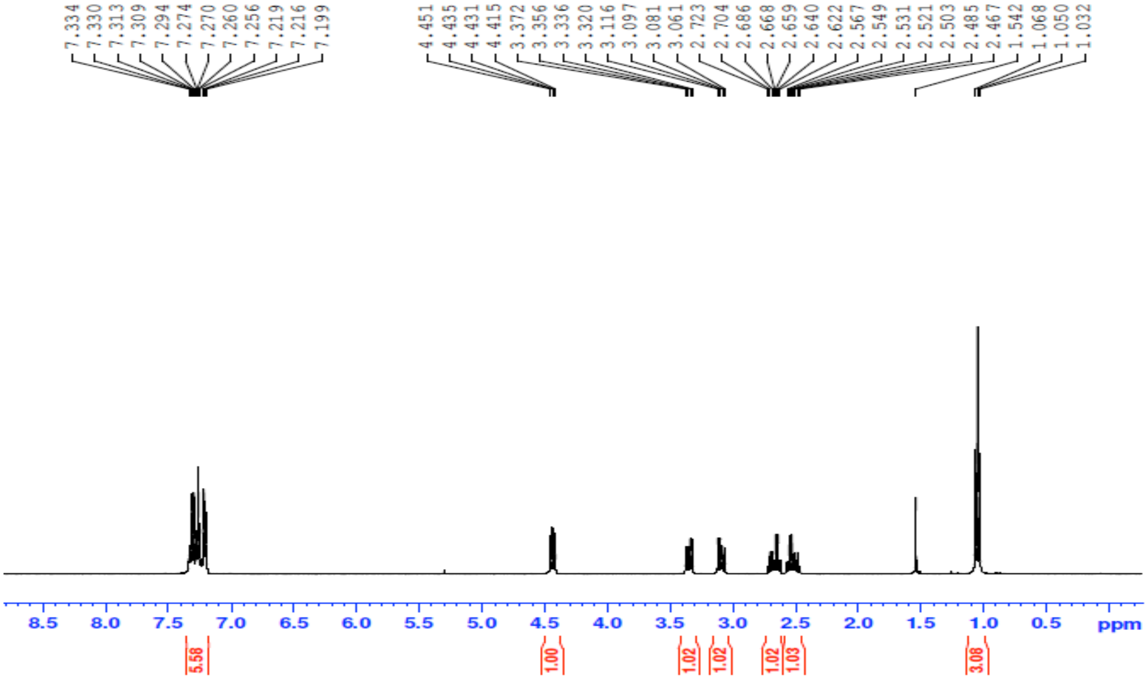

^{13}C NMR (100 MHz, $CDCl_3$)

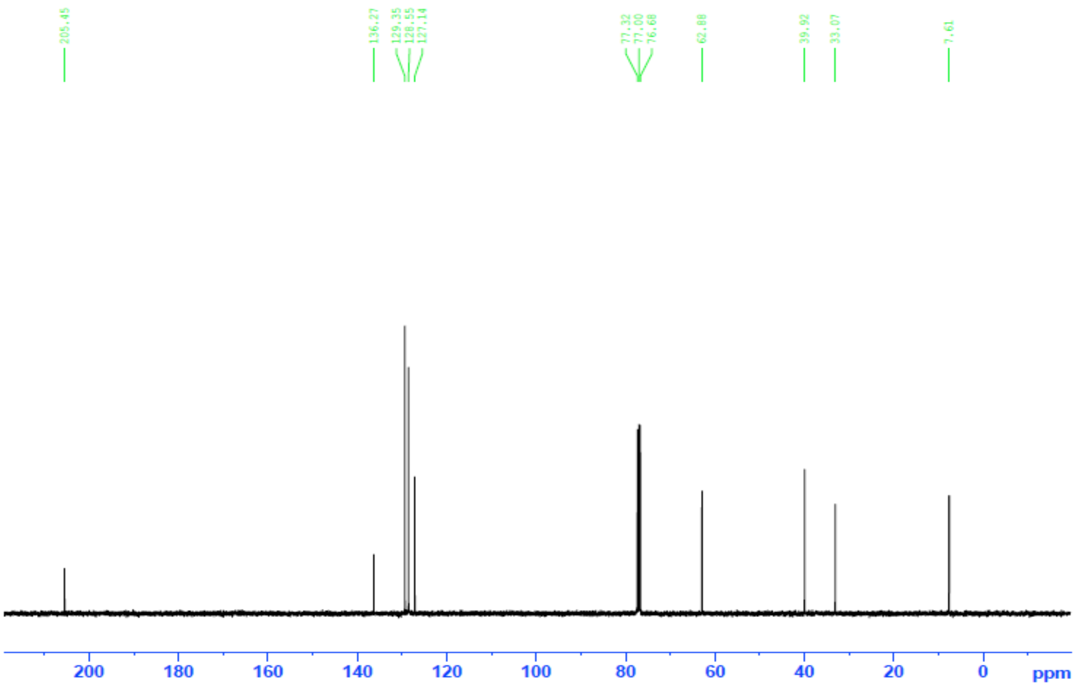

2-Chlorocyclohexanone (3i)

^{1}H NMR (400 MHz, $CDCl_3$). Crude spectra of **3i** against 0.5 mmol of internal standard. The final product was not isolted due to the high volatility.

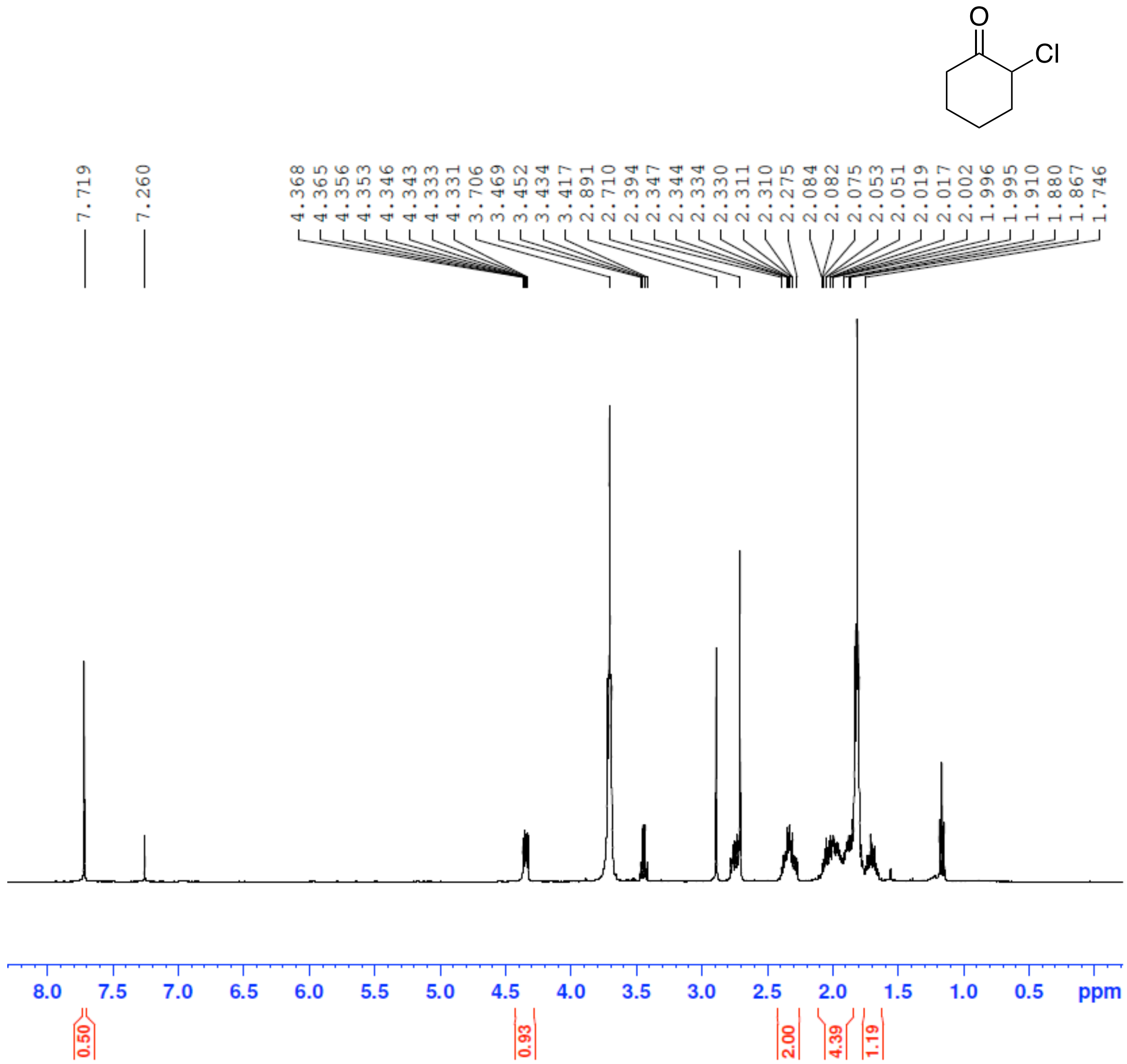

2-Chloro-5,9-dimethyldec-8-en-3-one (3j)

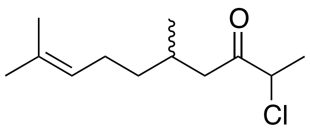

^{1}H NMR (400 MHz, $CDCl_3$)

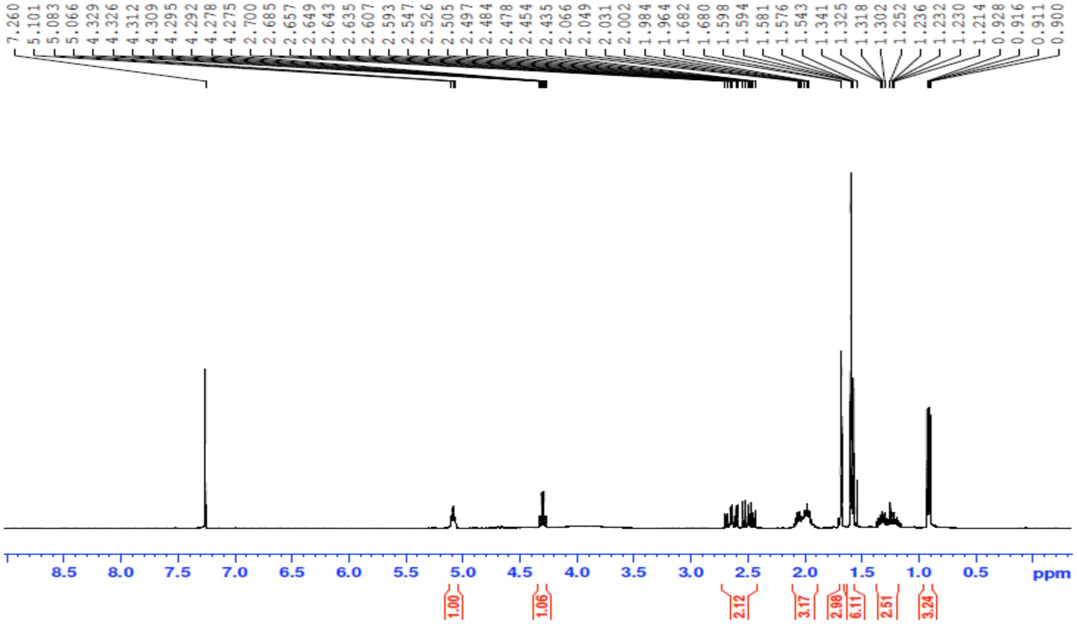

^{13}C NMR (100 MHz, $CDCl_3$)

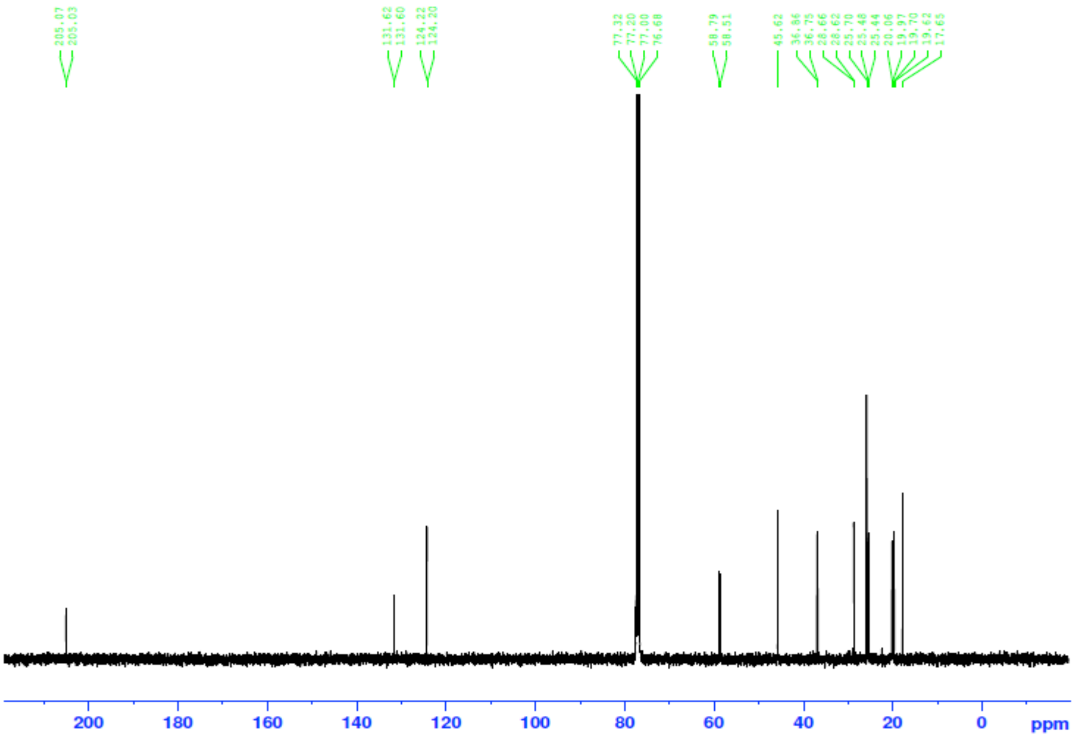

2-Chloro-1-((*R*)-1,4-dioxaspiro[4.5]decan-2-yl)propan-1-one (3k)

^{1}H NMR (400 MHz, $CDCl_3$)

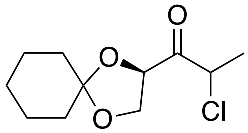

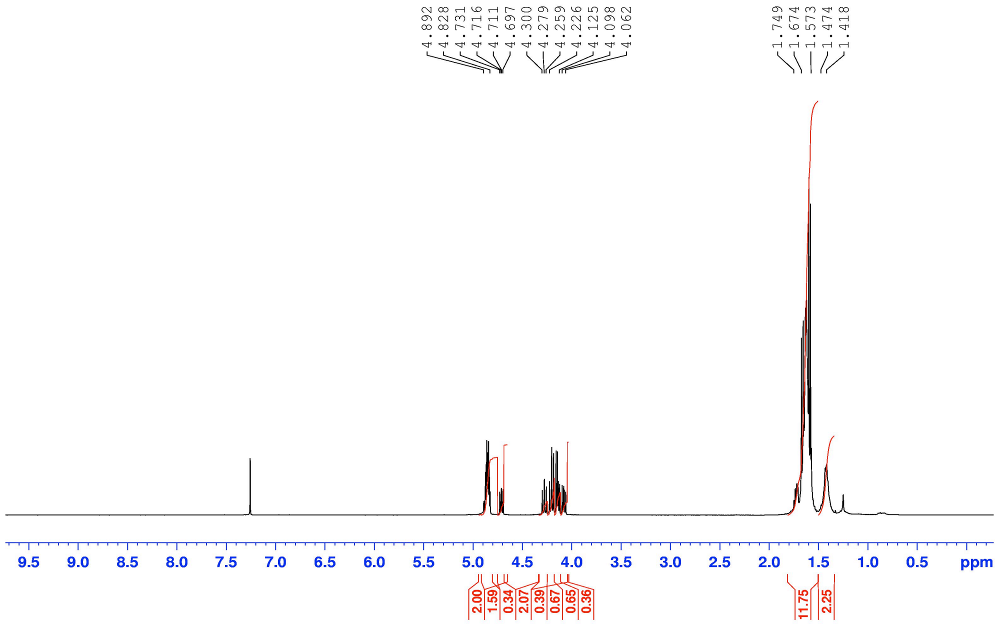

^{13}C NMR (100 MHz, $CDCl_3$)

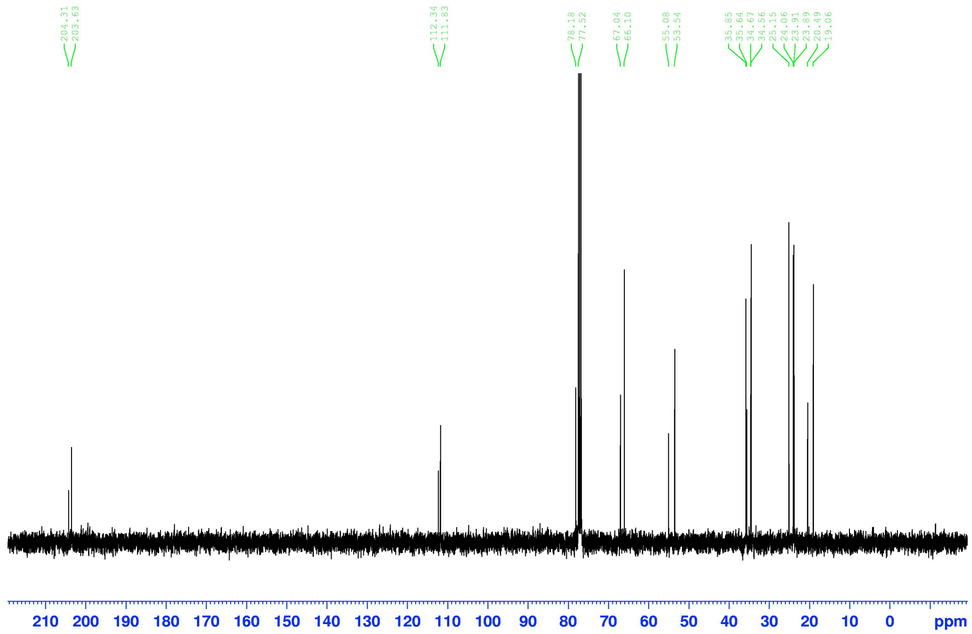

2-Chloro-1-phenylpropan-1-one (3l)

^{1}H NMR (400 MHz, $CDCl_3$)

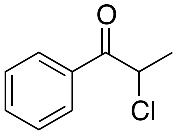

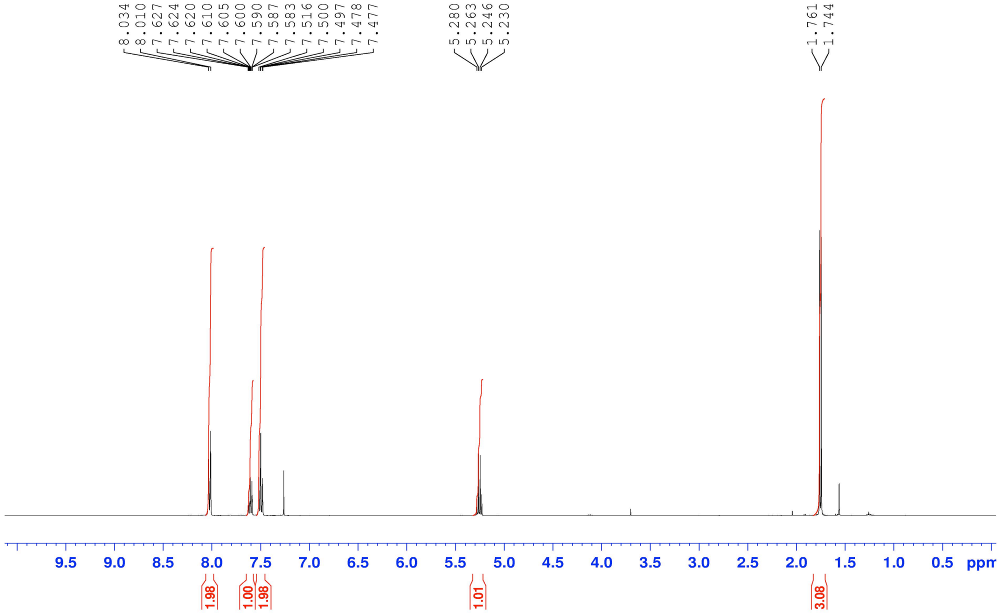

^{13}C NMR (100 MHz, $CDCl_3$)

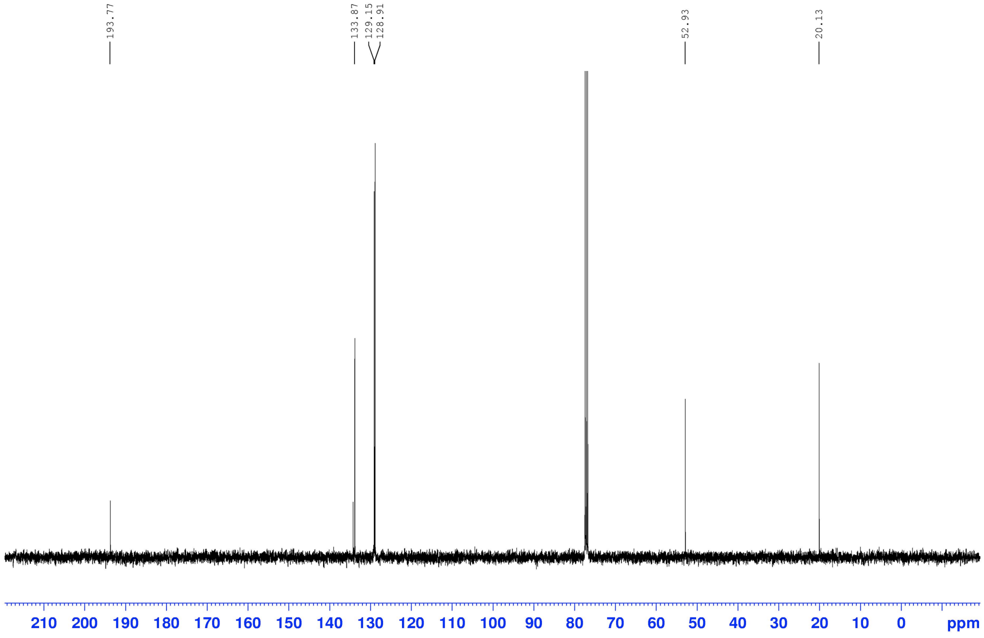

2-Chloro-1-(4-chlorophenyl)propan-1-one (3m)

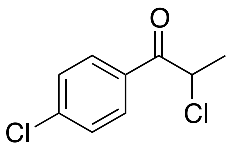

^{1}H NMR (400 MHz, $CDCl_3$)

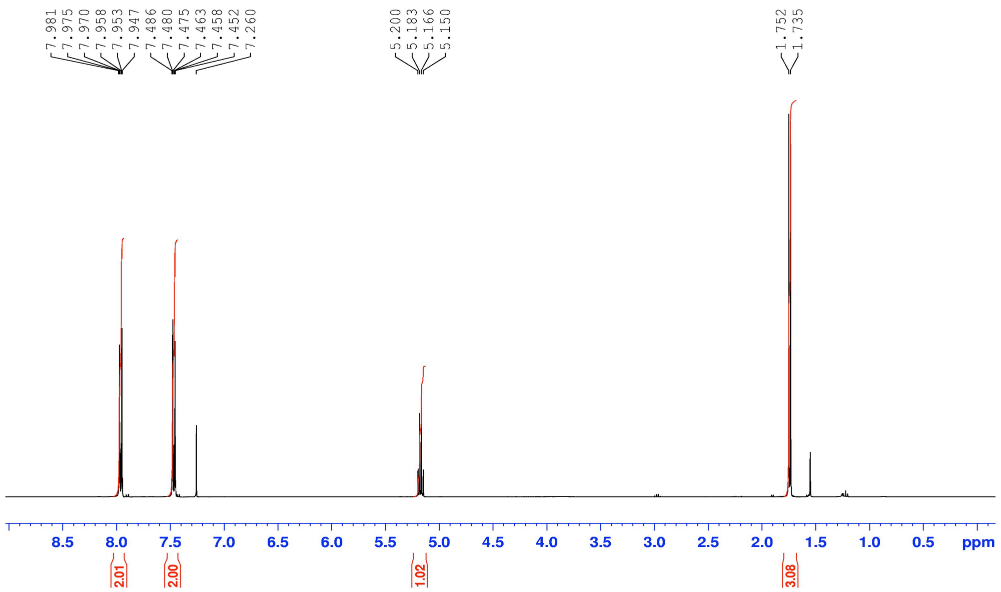

^{13}C NMR (100 MHz, $CDCl_3$)

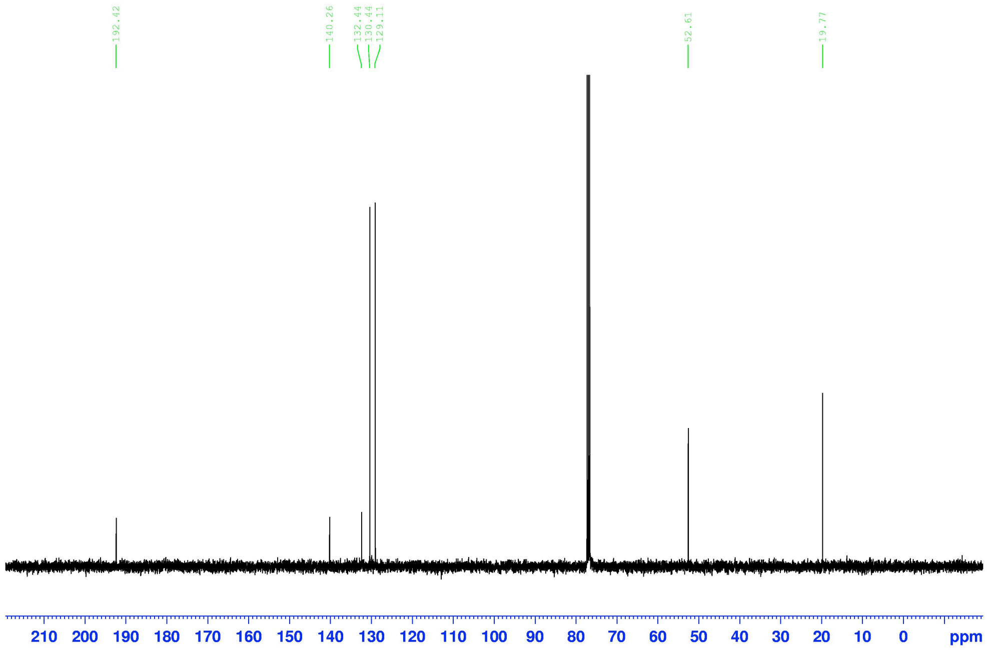

2-Chloro-1-(3-methoxyphenyl)propan-1-one (3n)

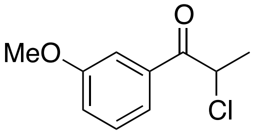

^{1}H NMR (400 MHz, CDCl$_3$)

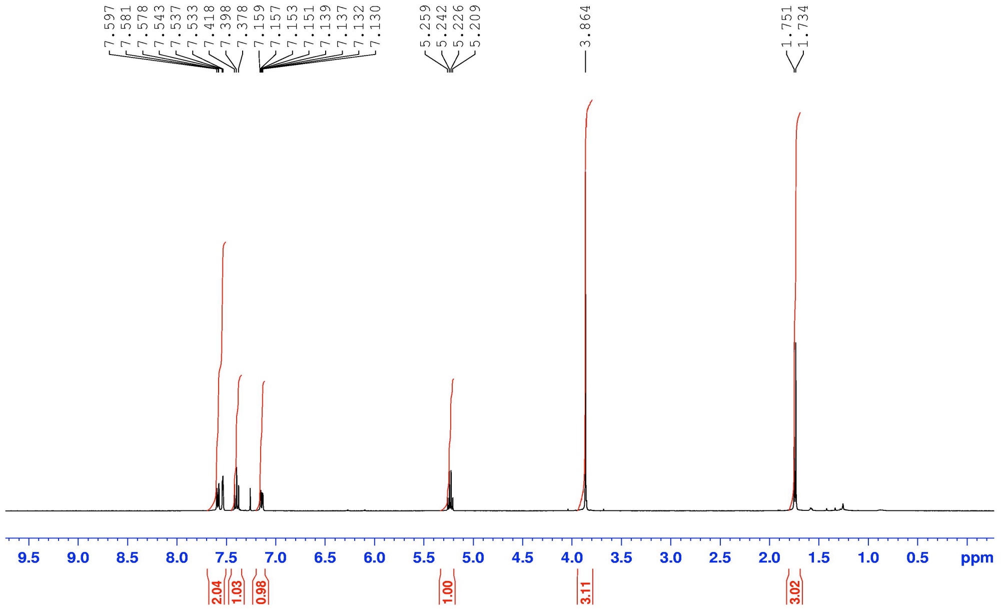

^{13}C NMR (100 MHz, CDCl$_3$)

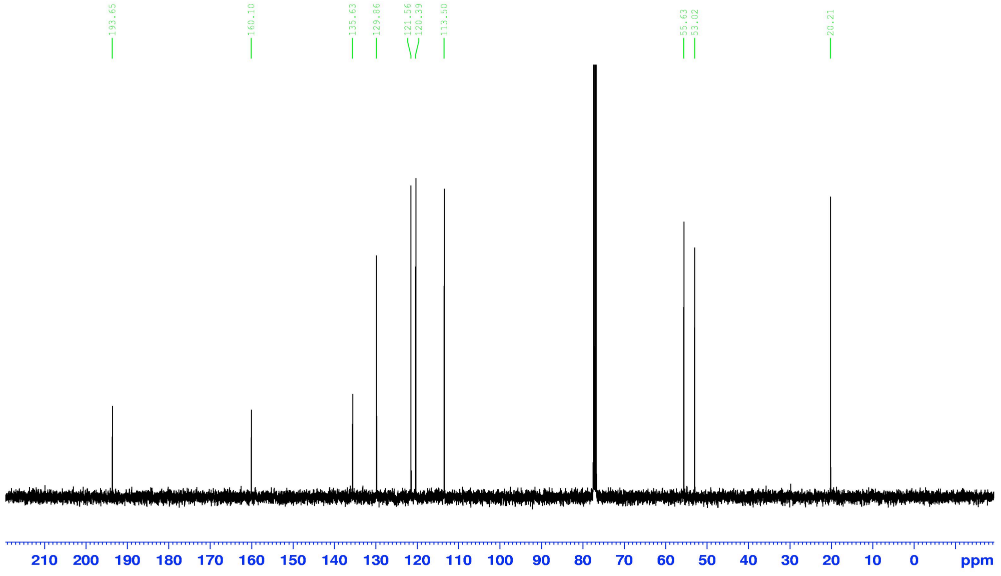

2-Chloro-1-(4-isobutylphenyl)propan-1-one (3o)

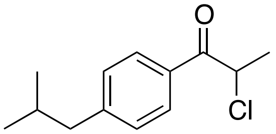

^{1}H NMR (400 MHz, $CDCl_3$)

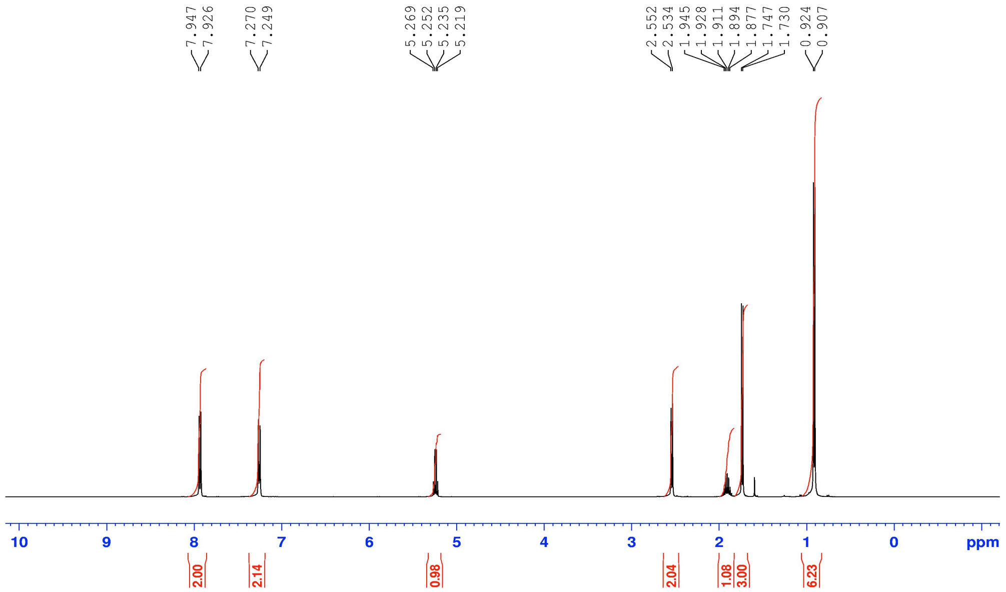

^{13}C NMR (100 MHz, $CDCl_3$)

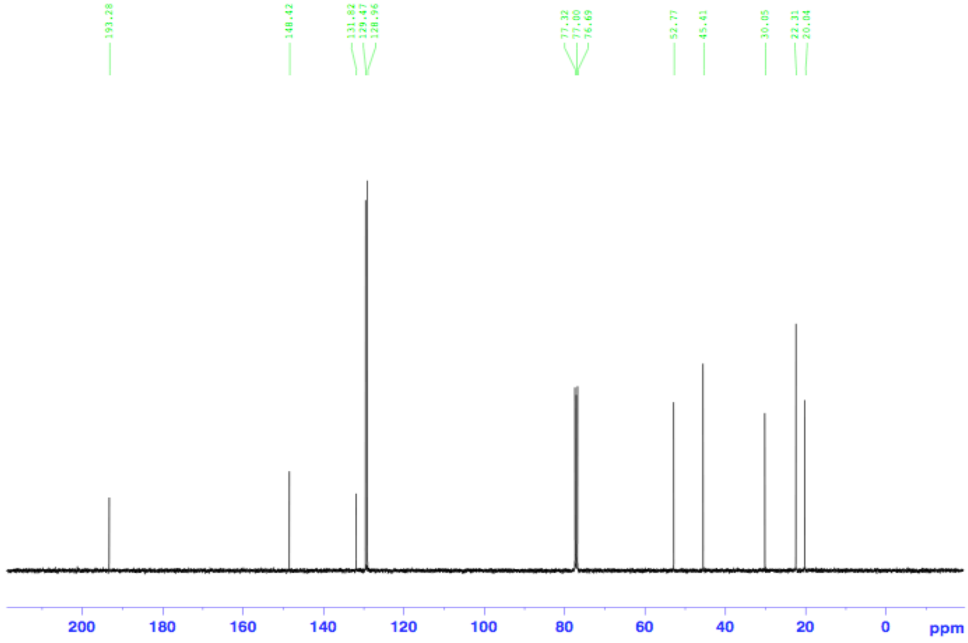

2-Chlorooctanal (7a)

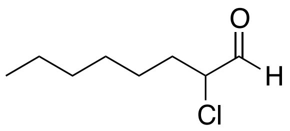

^{1}H NMR (400 MHz, $CDCl_3$)

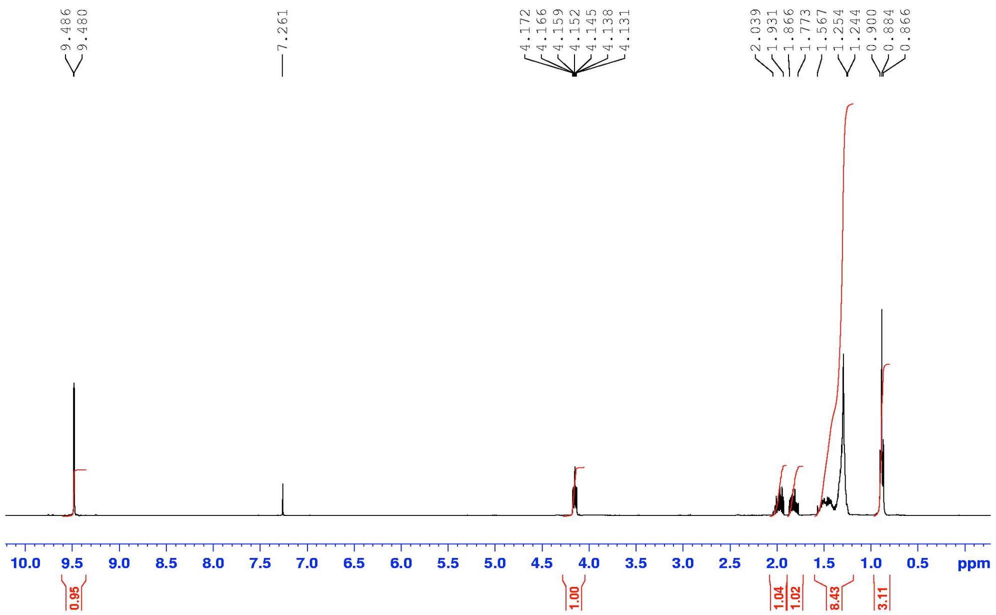

^{13}C NMR (100 MHz, $CDCl_3$)

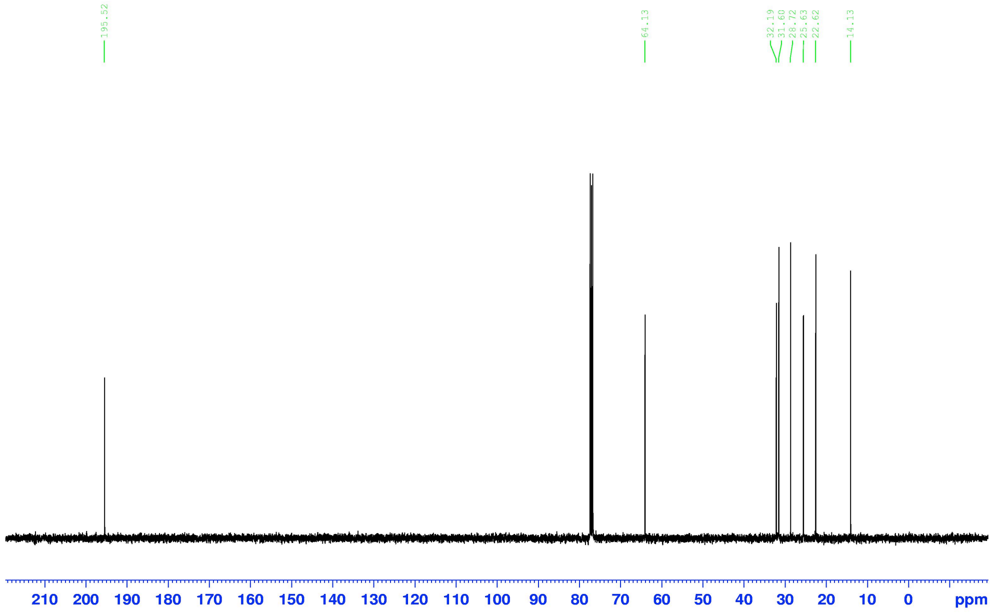

2-Chloro-3-phenylpropanal (7b)

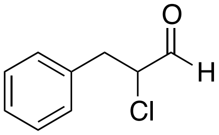

^{1}H NMR (400 MHz, $CDCl_3$)

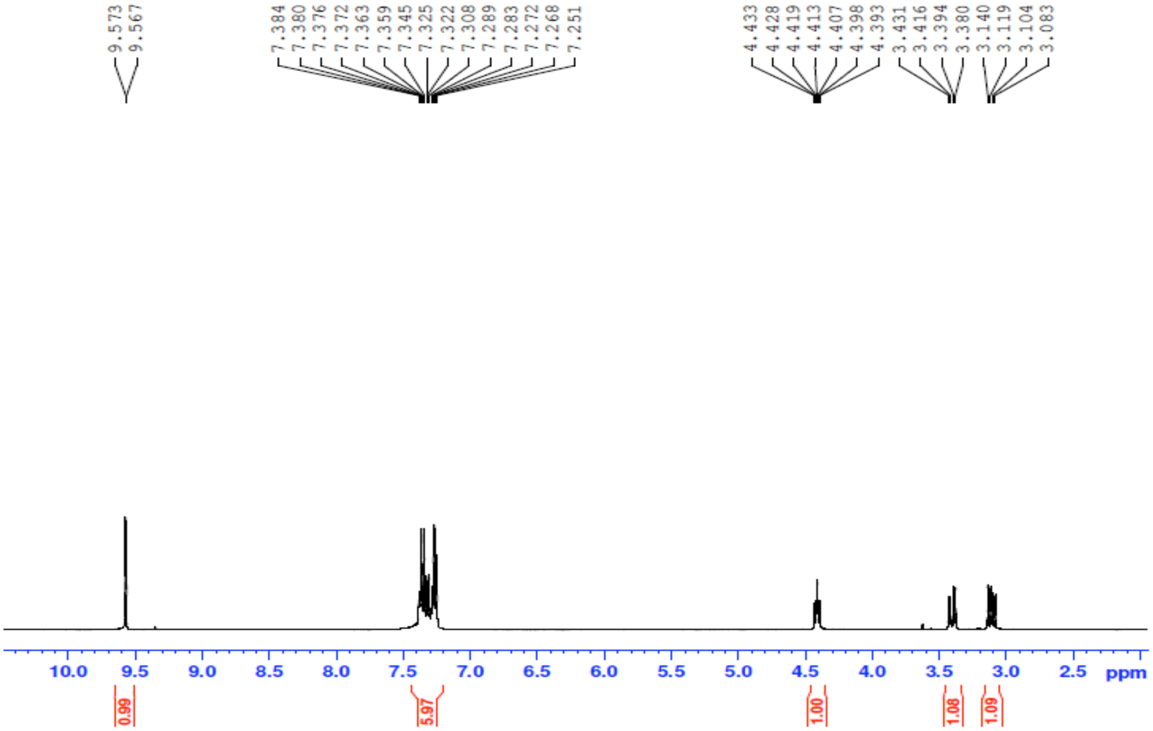

^{13}C NMR (100 MHz, $CDCl_3$)

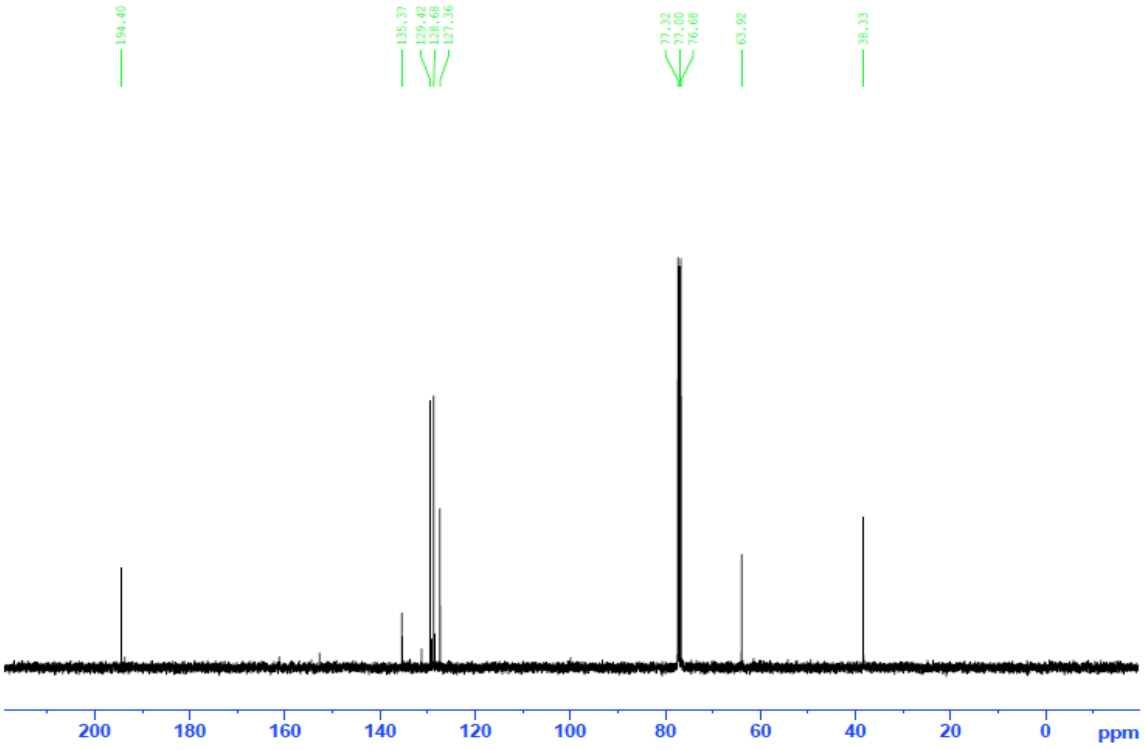

2-Chloro-5-phenylpentanal (7c)

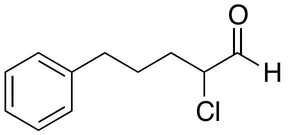

^{1}H NMR (400 MHz, $CDCl_3$)

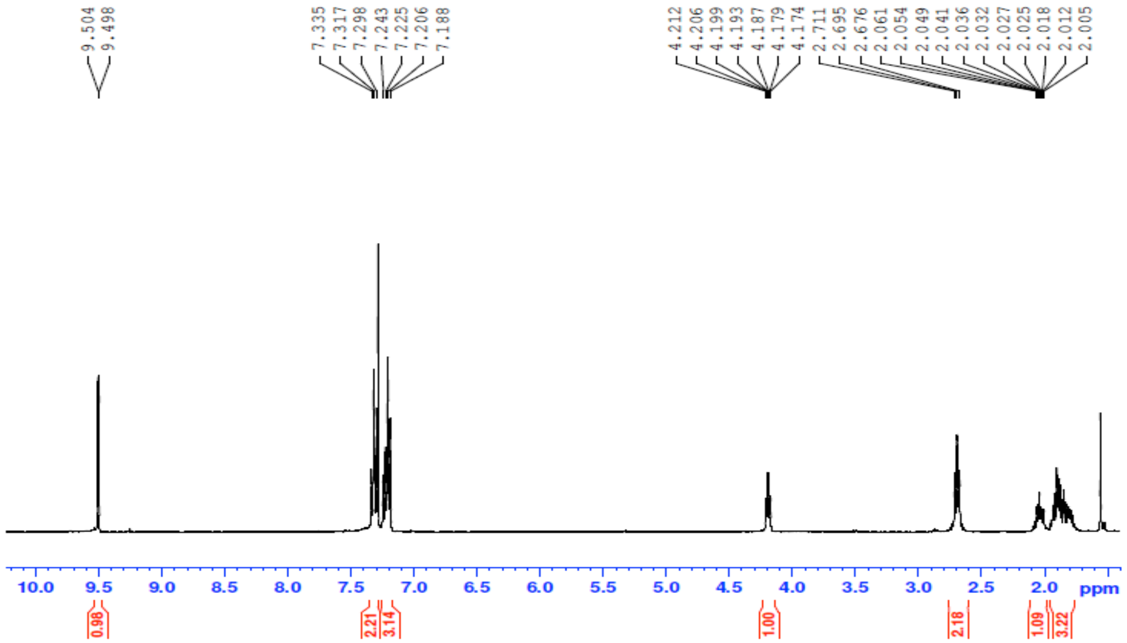

^{13}C NMR (100 MHz, $CDCl_3$)

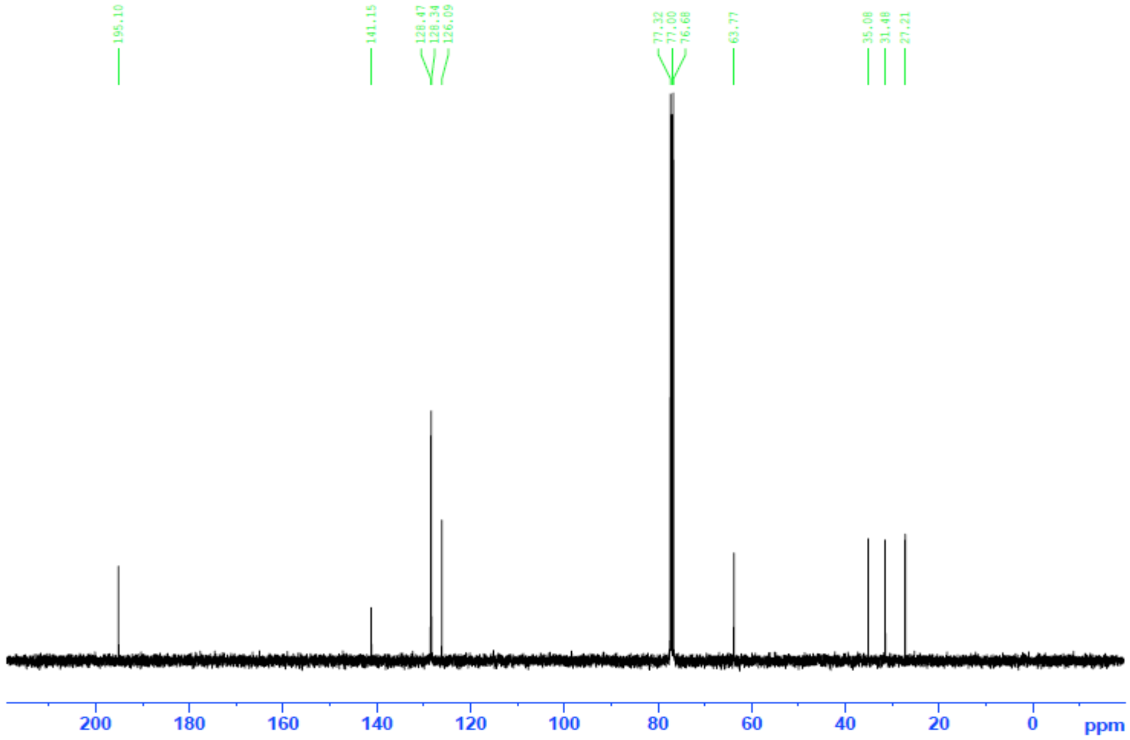

2-Chloro-3-(4-chlorophenyl)propanal (7d)

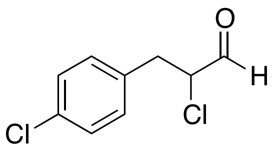

^{1}H NMR (400 MHz, $CDCl_3$)

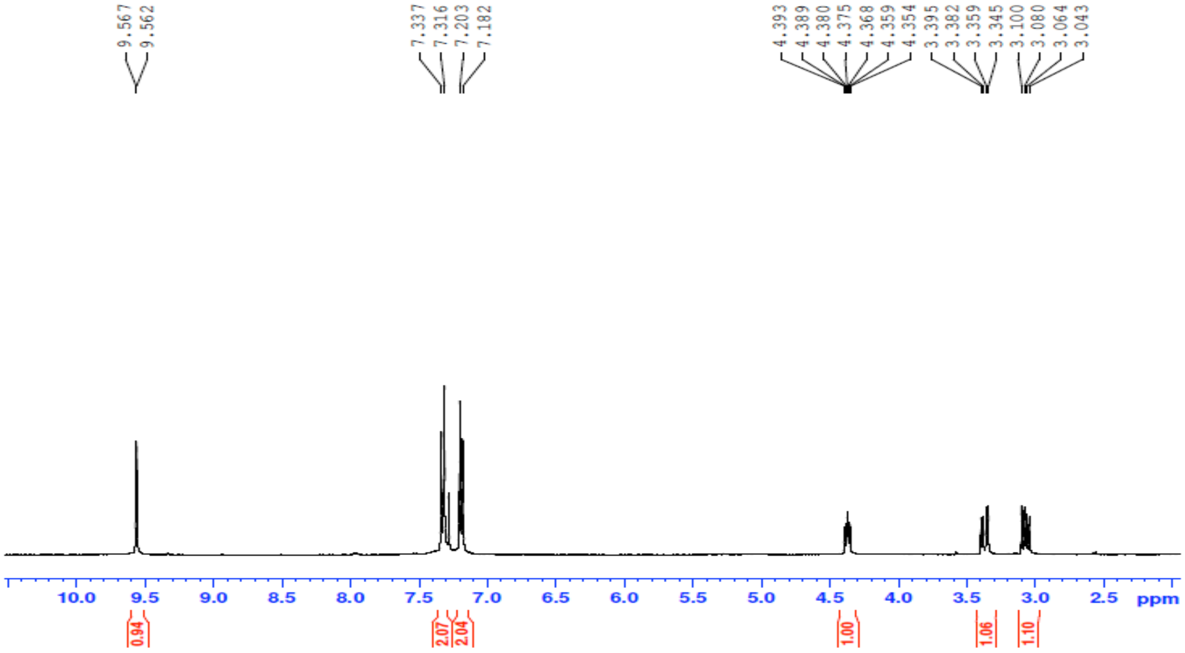

^{13}C NMR (100 MHz, $CDCl_3$)

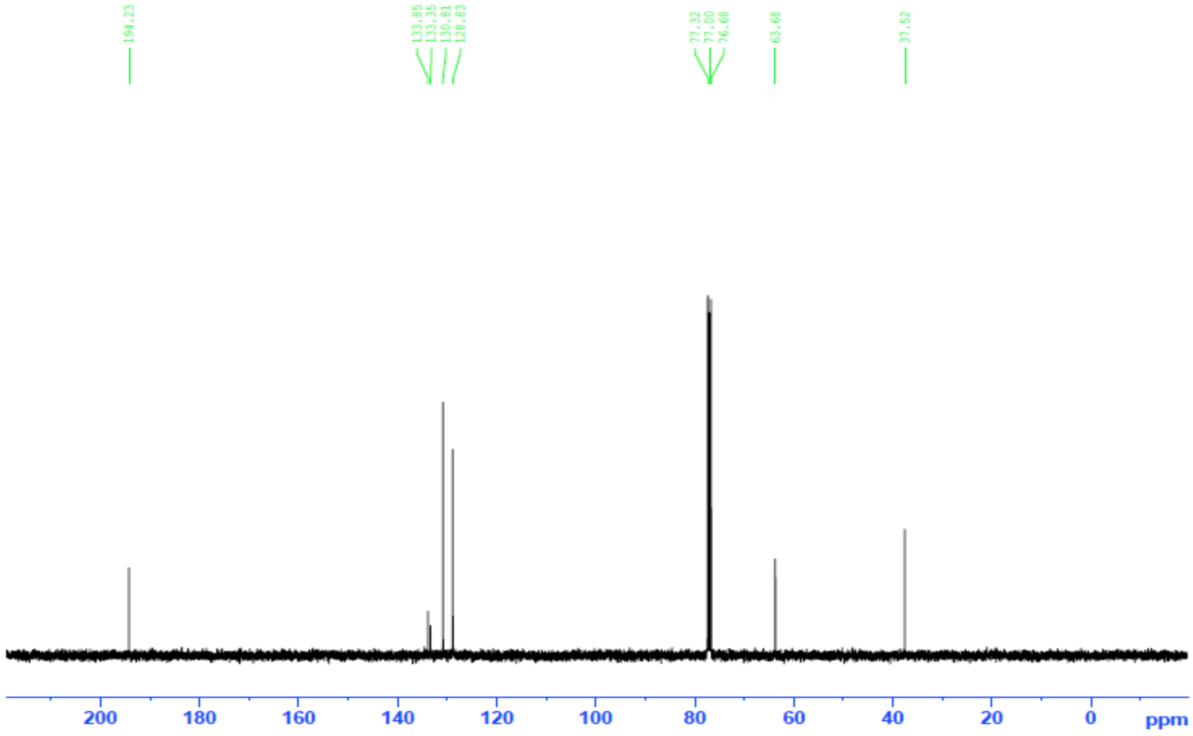

2-Chloro-3-(4-methoxyphenyl)propanal (7e)

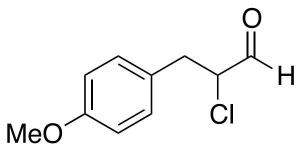

^{1}H NMR (400 MHz, $CDCl_3$)

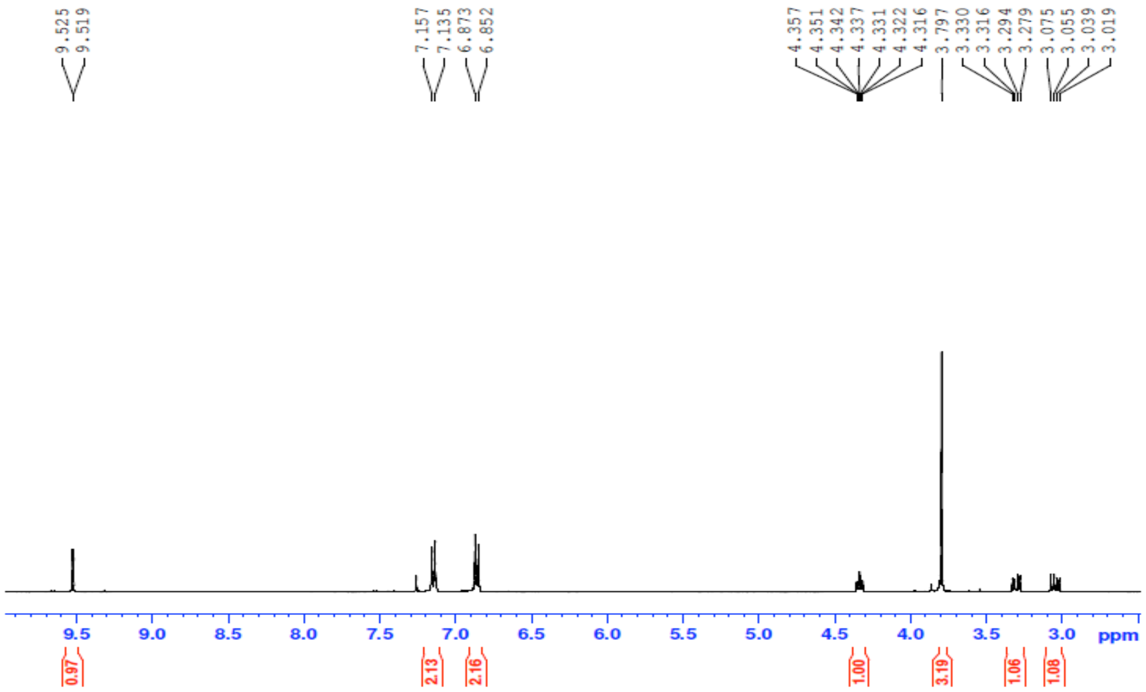

^{13}C NMR (100 MHz, $CDCl_3$)

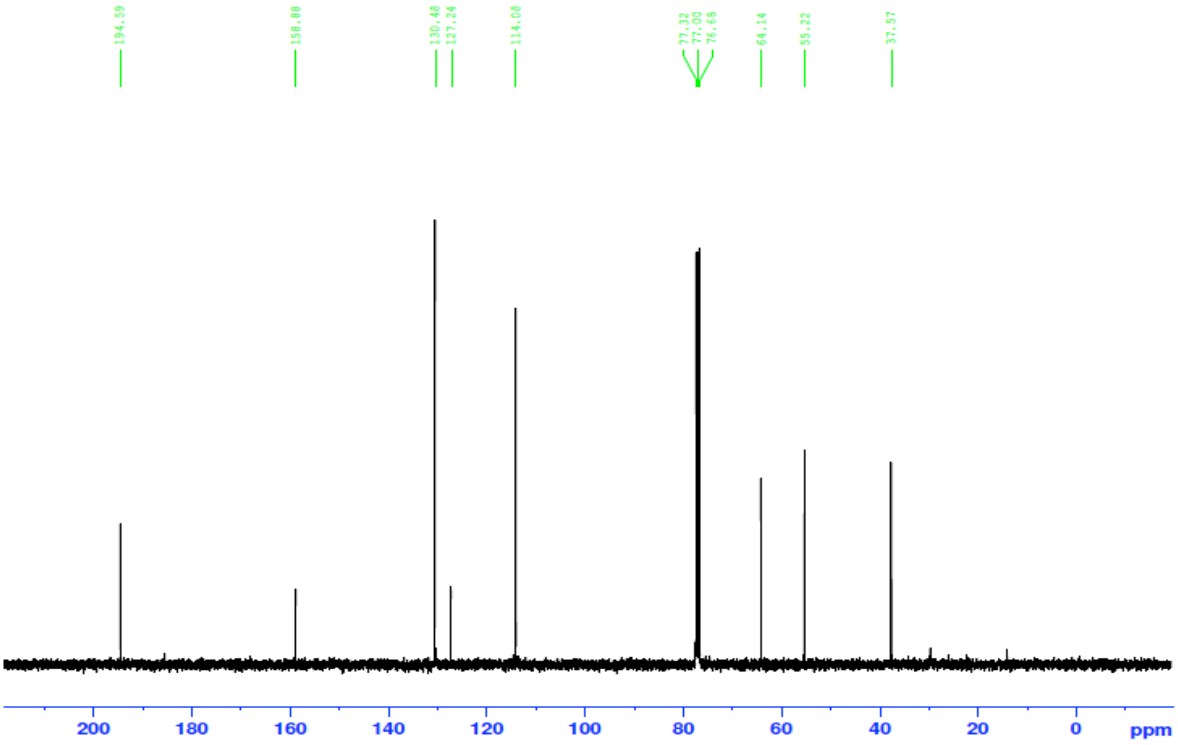

4-(Benzyloxy)-2-chlorobutanal (7f)

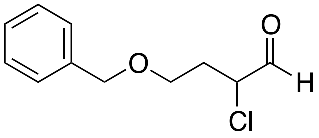

^{1}H NMR (400 MHz, $CDCl_3$)

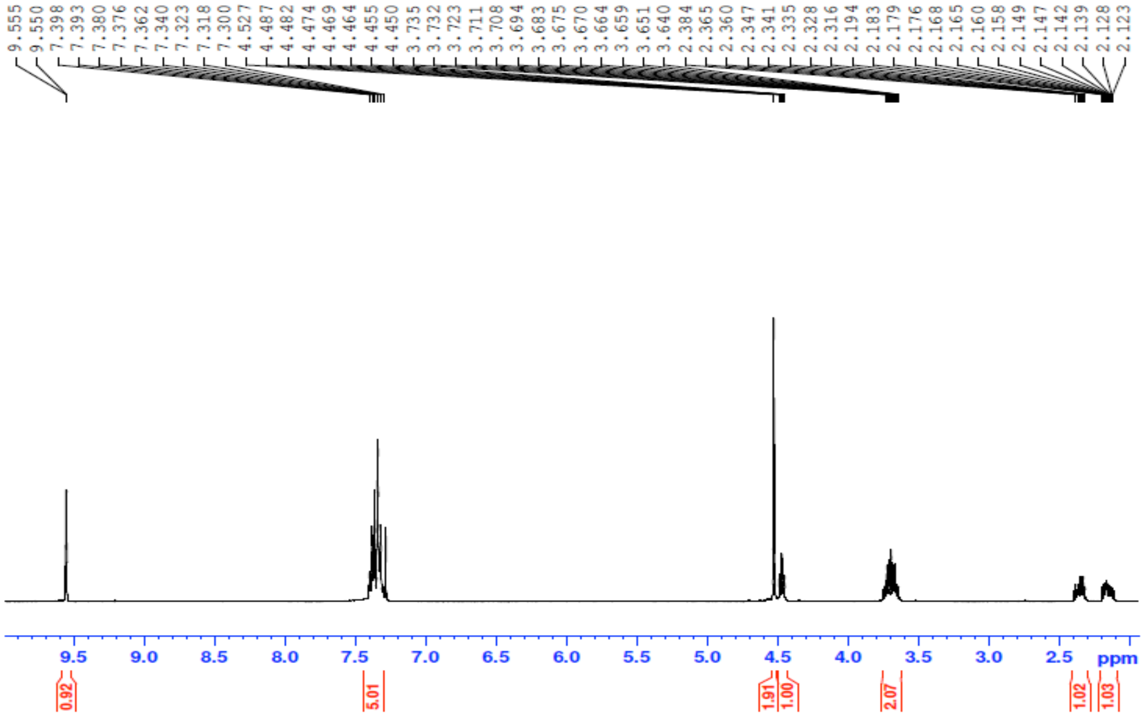

^{13}C NMR (100 MHz, $CDCl_3$)

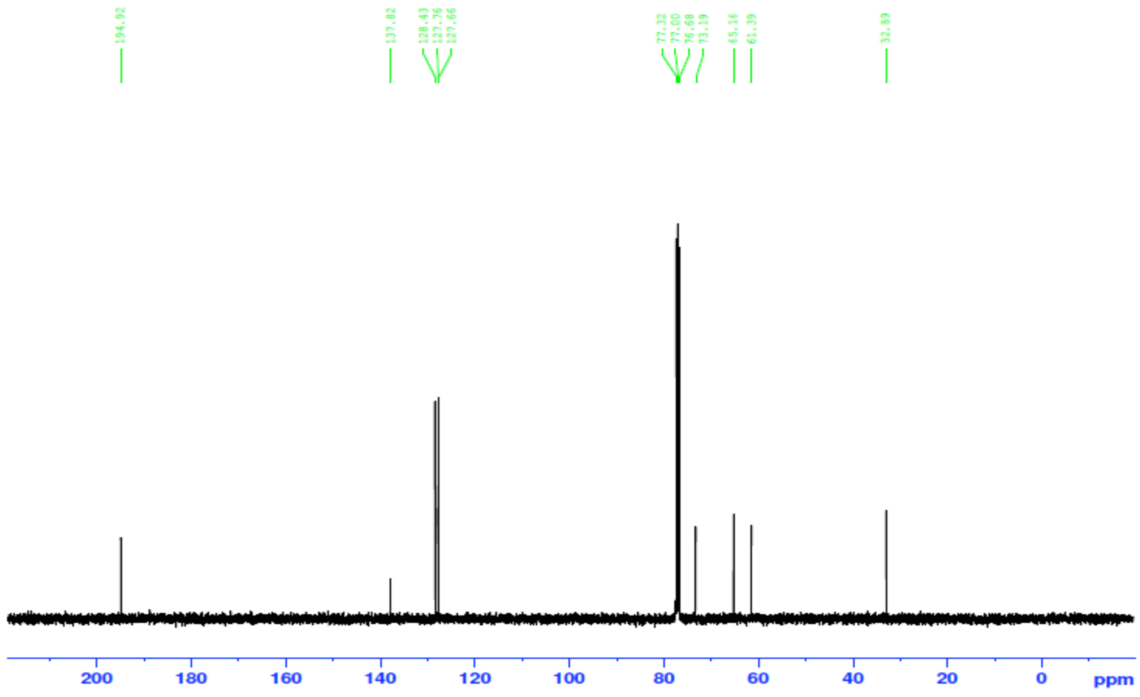

2-Chloro-1-(1,4-dioxaspiro[4.5]decan-2-yl)propan-1-one (rac-3k)

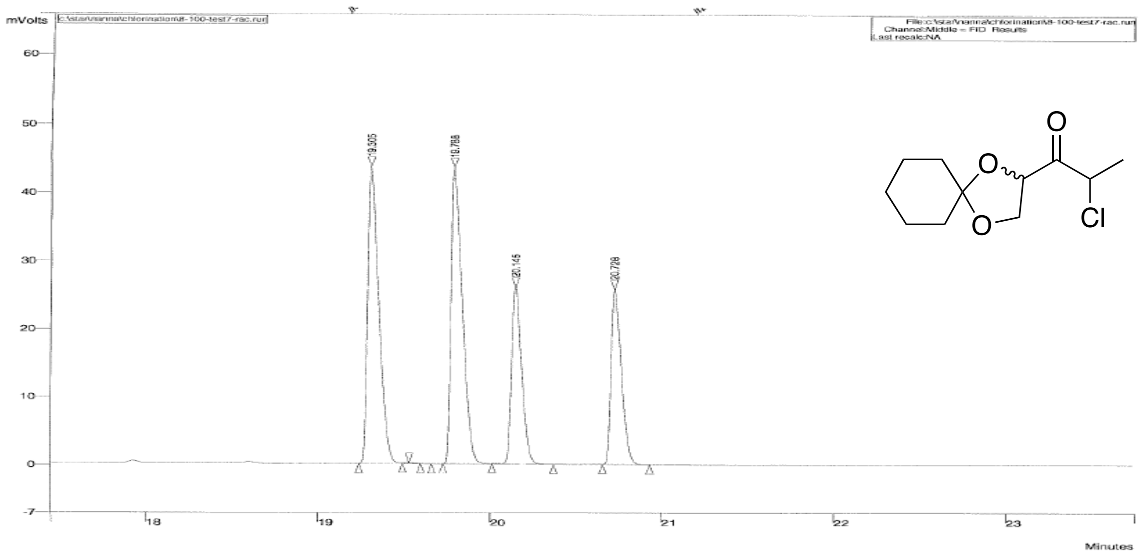

Peak No.	Peak Name	Result ()	Ret. Time (min)
1		32.5020	19.305
2		32.5704	19.788
3		17.5387	20.145
4		17.3889	20.728
	Totals:	100.0000	

2-Chloro-1-((*R*)-1,4-dioxaspiro[4.5]decan-2-yl)propan-1-one (3k)

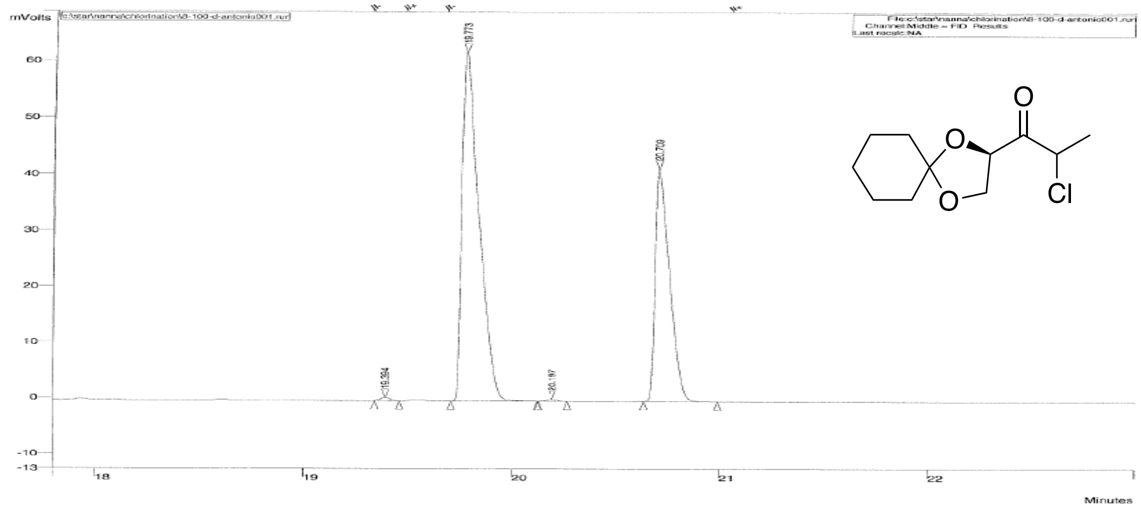

Peak No.	Peak Name	Result ()	Ret. Time (min)
1		0.3236	19.394
2		63.7084	19.773
3		0.1823	20.197
4		35.7858	20.709
	Totals:	100.0001	

5-Benzyl-4-methyl-2-aminothiazole (9)

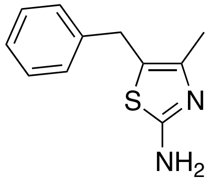

^{1}H NMR (400 MHz, $CDCl_3$)

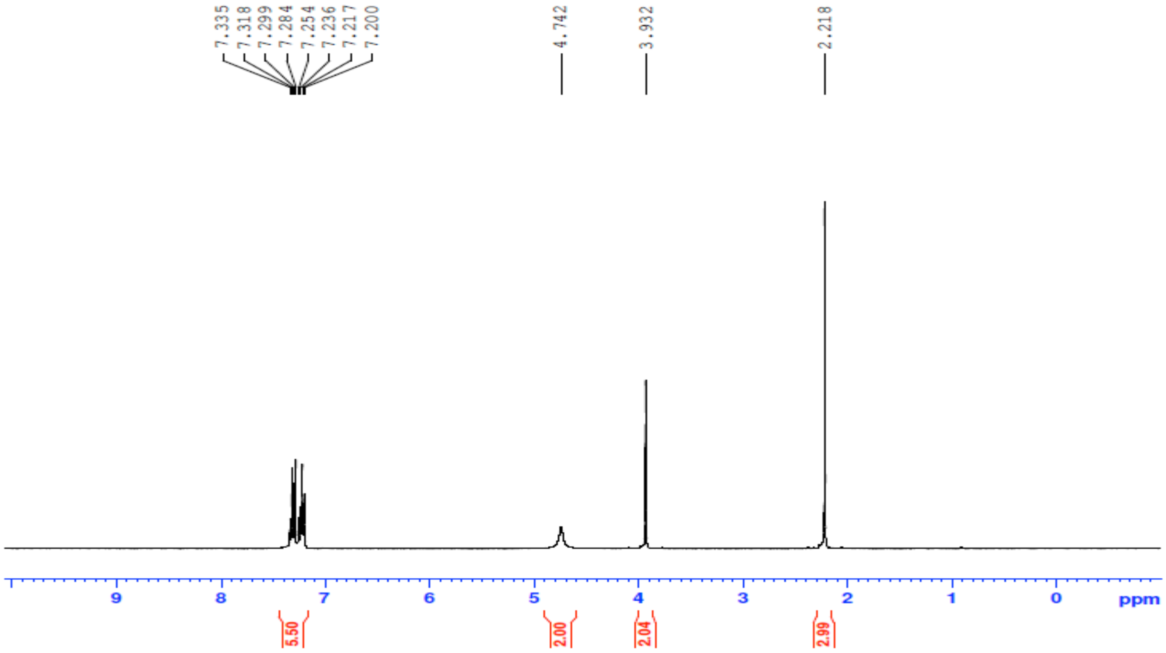

^{13}C NMR (100 MHz, $CDCl_3$)

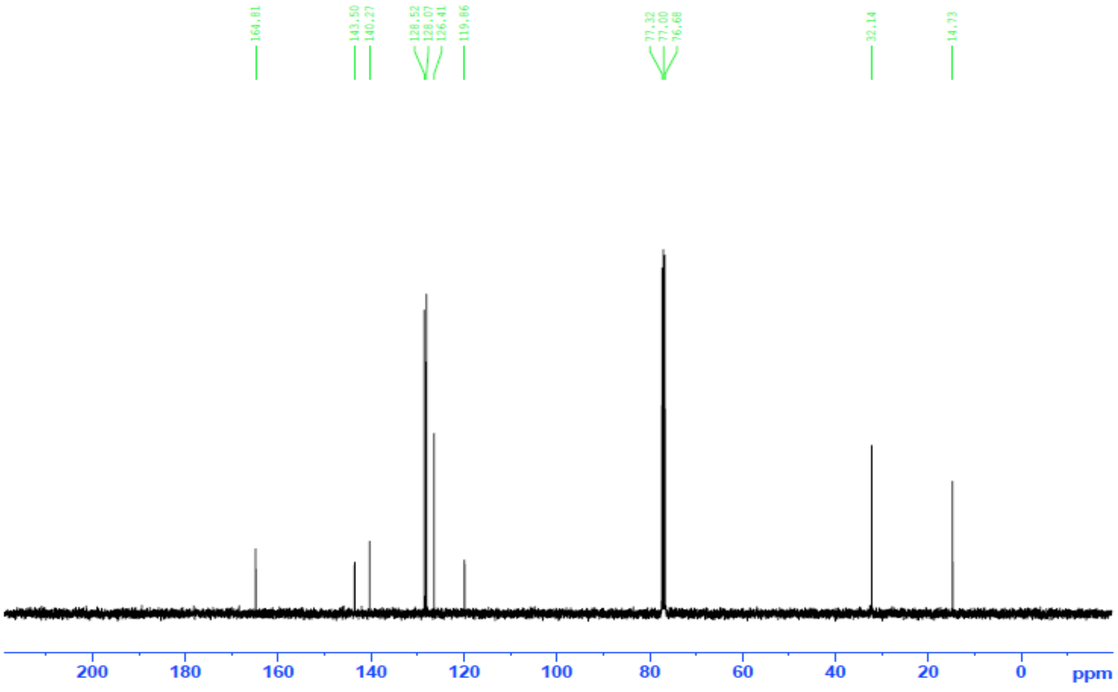

4-Benzyl-5-methyl-2-aminothiazole (10)

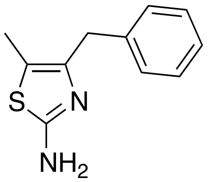

^{1}H NMR (400 MHz, $CDCl_3$)

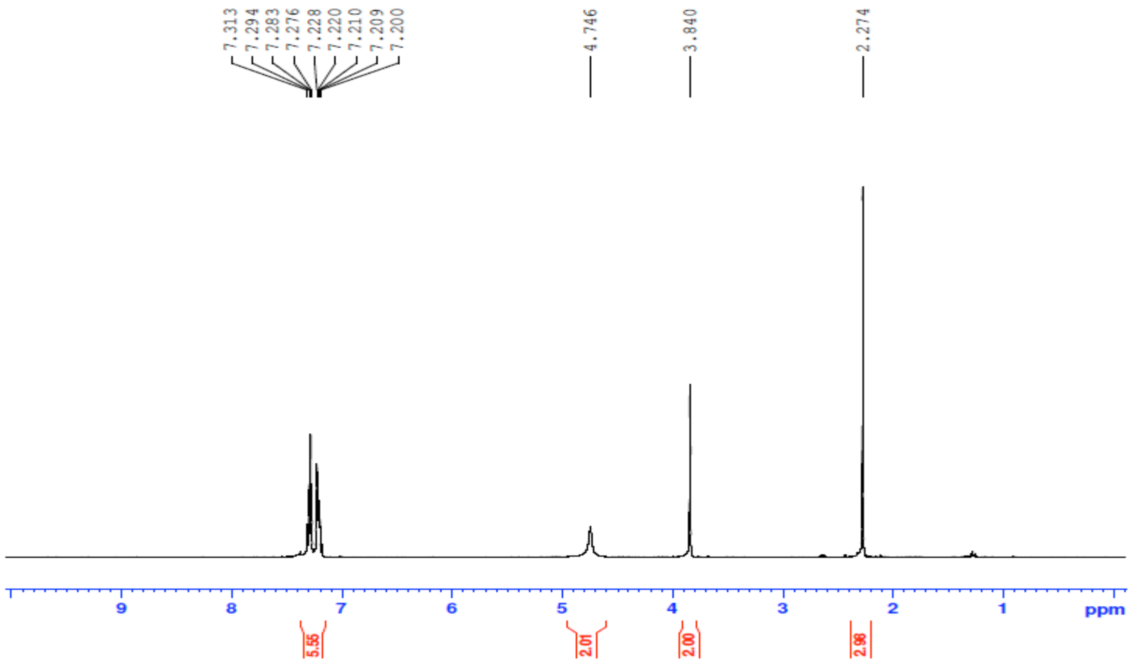

^{13}C NMR (100 MHz, $CDCl_3$)

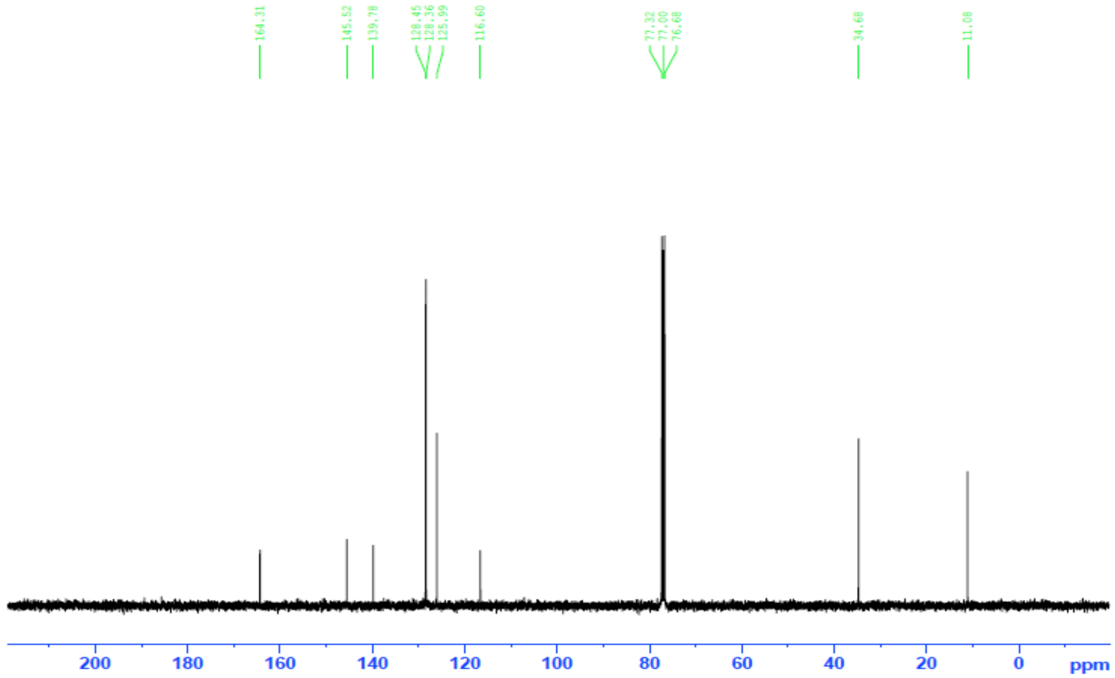

4-(4´-Isobutylphenyl)-5-methyl-2-aminothiazole (11)

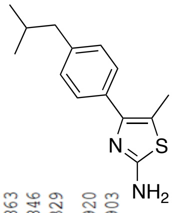

^{1}H NMR (400 MHz, $CDCl_3$)

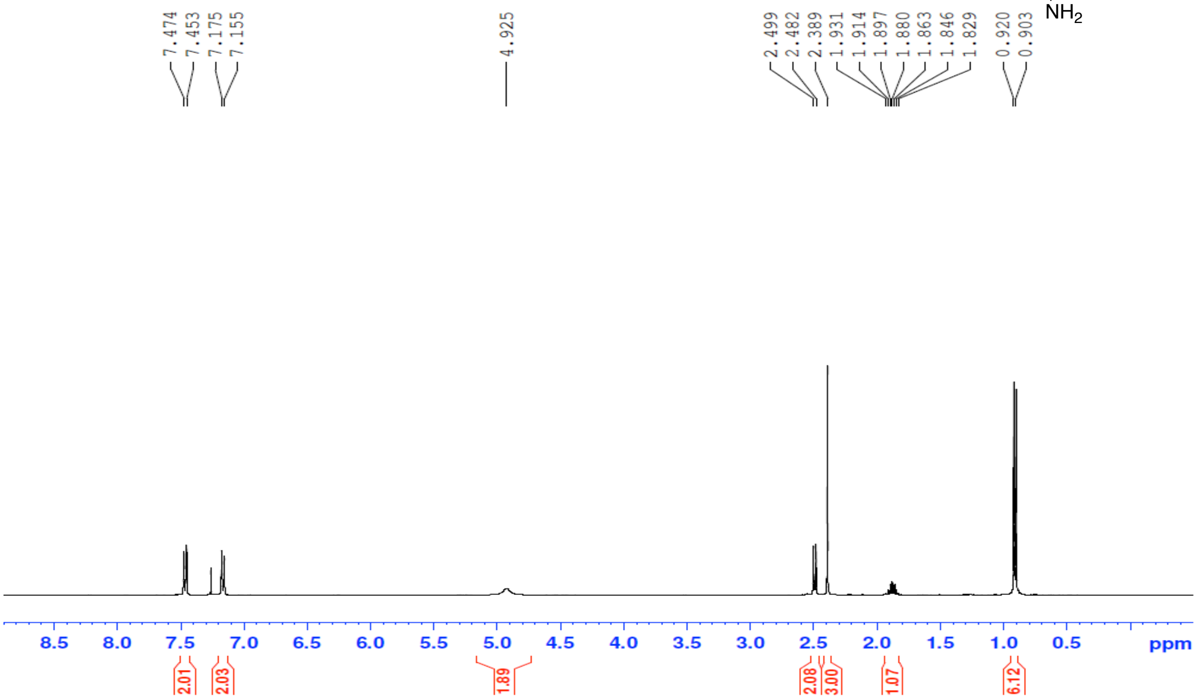

^{13}C NMR (100 MHz, $CDCl_3$)

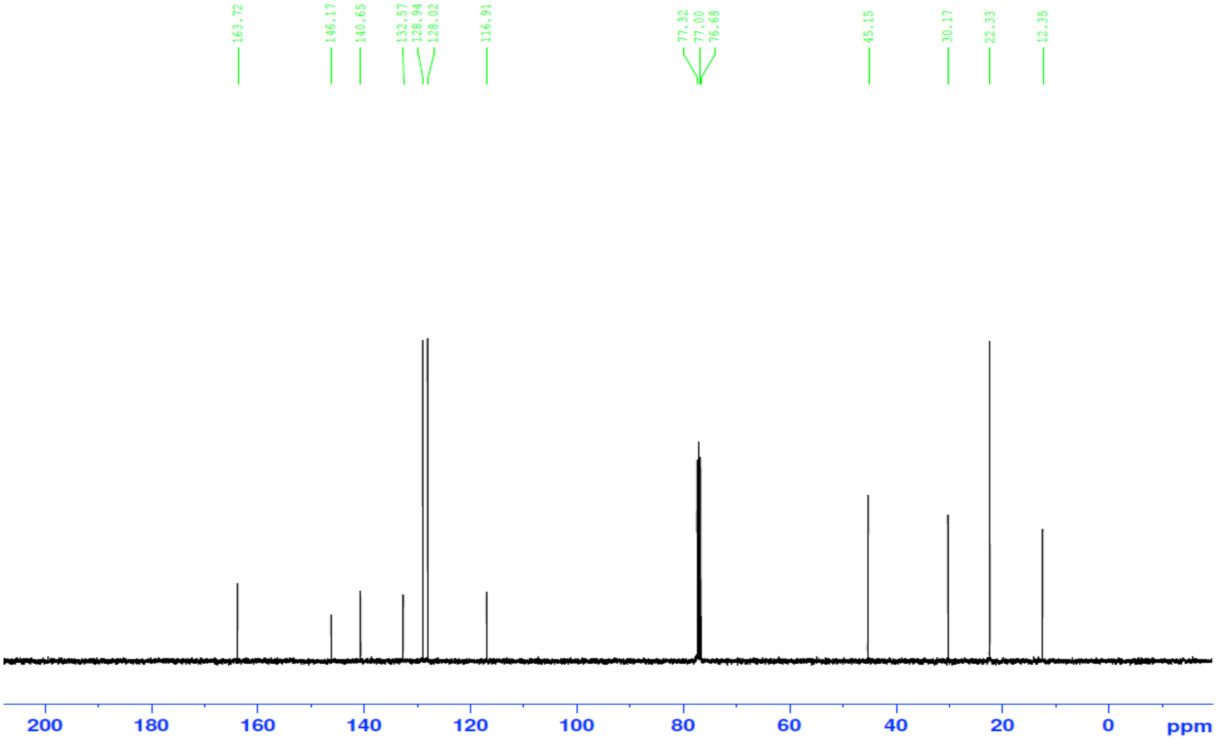

5-Methyl-4-pentyl-2-aminothiazole (12)

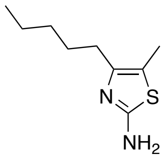

^{1}H NMR (400 MHz, $CDCl_3$)

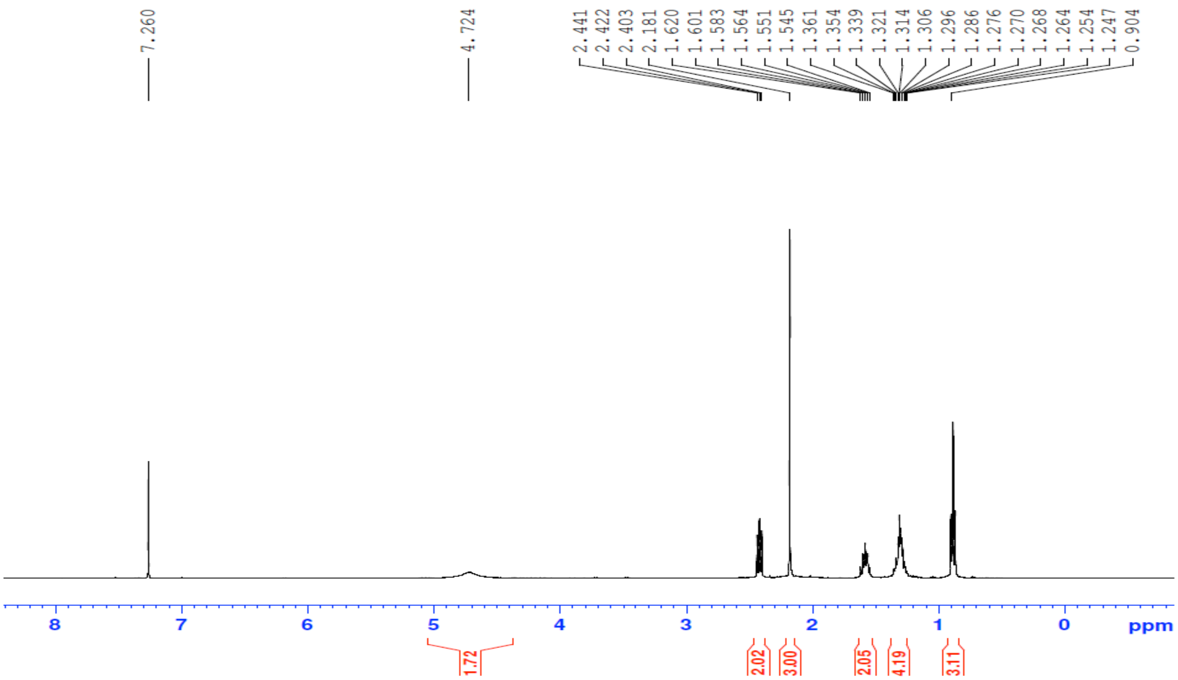

^{13}C NMR (100 MHz, $CDCl_3$)

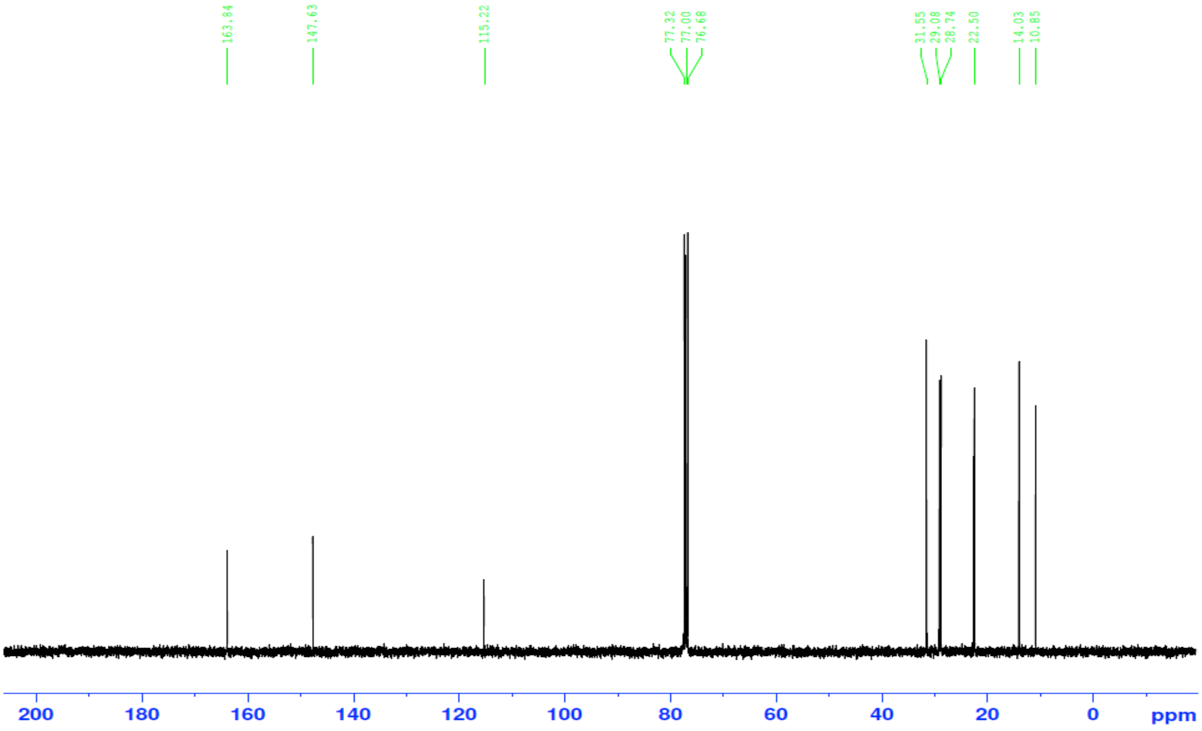

[1] a) N. A. Owston, A. J. Parker, J. M. J. Williams, *Org. Lett.* **2006**, *9*, 73. b) S. Ogo, N. Makihara, Y. Watanabe, *Organometallics* **1999**, *18*, 5470.
[2] M. B. Onaran, C. T. Seto, *J. Org. Chem.* **2003**, *68*, 8136.
[3] B. G. Kelly, D. G. Gilheany, *Tetrahedron Lett.* **2002**, *43*, 887.
[4] T. Borg, J. Danielsson, M. Mohiti, P. Restorp, P. Somfai, *Adv. Synth. Catal.* **2011**, *353*, 2022.
[5] R. Balamurugan, R. B. Kothapalli, G. K. Thota, *Eur. J. Org. Chem.* **2011**, 1557.
[6] M. Radi, G. Casaluce, M. Botta, *Synlett* **2011**, *14*, 1997.
[7] M. Körner, M. Hiersemann, *Org. Lett.* **2007**, *9*, 4979.
[8] L. J. Gazzard, W. B. Motherwell, D. A. Sandham, *J. Chem. Soc., Perkin Trans. 1* **1999**, 979
[9] A. Chattopadhyay, V. R. Mamdapur, *J. Org. Chem.* **1995**, *60*, 585.
